# Co-Catalyzed Asymmetric Hydrogenation. The Same Enantioselection Pattern for Different Mechanisms

**DOI:** 10.3390/ijms24065568

**Published:** 2023-03-14

**Authors:** Ilya D. Gridnev

**Affiliations:** N. D. Zelinsky Institute of Organic Chemistry, Russian Academy of Sciences, Leninsky Prospekt 47, 119911 Moscow, Russia; ilyaiochem@gmail.com

**Keywords:** asymmetric catalysis, asymmetric hydrogenation, DFT computation, mechanism of stereoselection, weak intramolecular interactions

## Abstract

The mechanism of the recently reported catalyzed asymmetric hydrogenation of enyne **1** catalyzed by the Co-(*R*,*R*)-QuinoxP* complex was studied by DFT. Conceivable pathways for the Co(I)-Co(III) mechanism were computed together with a Co(0)-Co(II) catalytic cycle. It is commonly assumed that the exact nature of the chemical transformations taking place along the actually operating catalytic pathway determine the sense and level of enantioselection of the catalytic reaction. In this work, two chemically different mechanisms reproduced the experimentally observed perfect stereoselection of the same handedness. Moreover, the relative stabilities of the transition states of the stereo induction stages were controlled via exactly the same weak disperse interactions between the catalyst and the substrate.

## 1. Introduction

Obtaining a better understanding of the intrinsic mechanisms for generating chirality via asymmetric catalysis is one of the most important current targets of chemical research [1,2]. Although significant progress has been made in the experimental characterization of reactive intermediates, as well as in computational studies of various catalytic cycles, the results accumulated to date lack sufficient generalization and classification, with each particular reaction being considered as a specific case with its own mechanism of chirality generation. Even the simplest conceivable asymmetric catalytic reaction, viz., asymmetric hydrogenation, is a field comprising a lot of different suggestions, mechanistic ideas, reconsiderations, empirical rules, etc. [3,4,5,6,7,8,9,10,11,12,13,14,15,16,17,18,19,20,21,22,23].

This situation has become still more complicated with the recent rapid development of new hydrogenation techniques applying asymmetric catalysis with complexes of cheap and abundant metals [24,25,26]. Numerous mechanistic studies of the catalytic cycles of asymmetric hydrogenations have been performed of catalysis by earth-abundant metals, such as Ni [27,28,29,30,31], Co [32,33,34,35,36,37,38,39,40,41,42,43,44,45,46,47], Fe [48,49,50,51,52], Mn [53,54], etc. It seems that each metal has its own hydrogenation chemistry, which can in turn be subdivided into various catalytic cycles with different metal oxidation states.

**Scheme 1:**
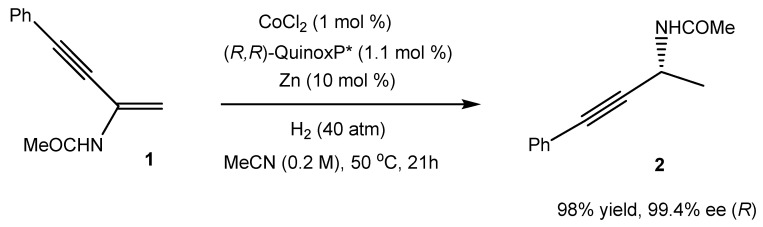
Asymmetric hydrogenation of **1**.

Recently, Zhang et al. reported co-catalyzed asymmetric hydrogenation of enynes and suggested Co(I)-Co(III), with a mechanism based on analogous Rh-catalyzed reactions being suggested (Scheme 1) [46]. Being interested in the mechanisms of stereoselection in various catalytic asymmetric reactions, the author decided to study the mechanism of this new reaction computationally. Unexpectedly it was found that different mechanisms, either Co(I)-Co(III) or Co(0)-Co(II), which are dissimilar in their chemical details and the structures of their intermediates, predict the same sense and level of enantioselectivity.

The author is convinced that this is an important finding for a general theory of asymmetric catalysis, and presents the results in this paper.

## 2. Results and Discussion

The authors suggest a Co(I)-Co(III) mechanism for the reaction based on the analogous synthesis of the corresponding Rh-catalyzed hydrogenation [46]. Hence, the corresponding competing catalytic cycles were first computed.

### 2.1. Formation of Solvate Dihydrides

**Scheme 2:**
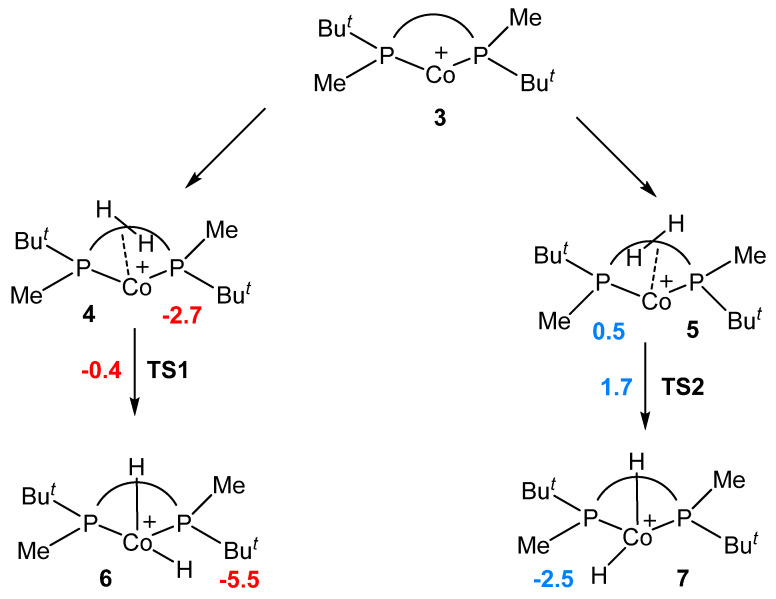
Formation of dihydride complexes **6** and **7**. The solvent molecules were not computed explicitly, and are not shown in this schematic representation. Computed free energies are given relative to **3** + **1** +H_2_ (shown in red and blue for opposite enantiomers).

QuinoxP*-Co(I) complex **3** was computed to undergo facile hydrogenation yielding diastereomeric solvate dihydrides **6** and **7** via initial formation of molecular hydrogen complexes **4** and **5** (Scheme 2).

It was concluded that these hydrogenations are suitable reactions for the dihydrogen activation and can make a start for a Co(I)-Co(III) catalytic cycle.

### 2.2. Oxidative Addition to Catalyst–Substrate Complexes

**Scheme 3:**
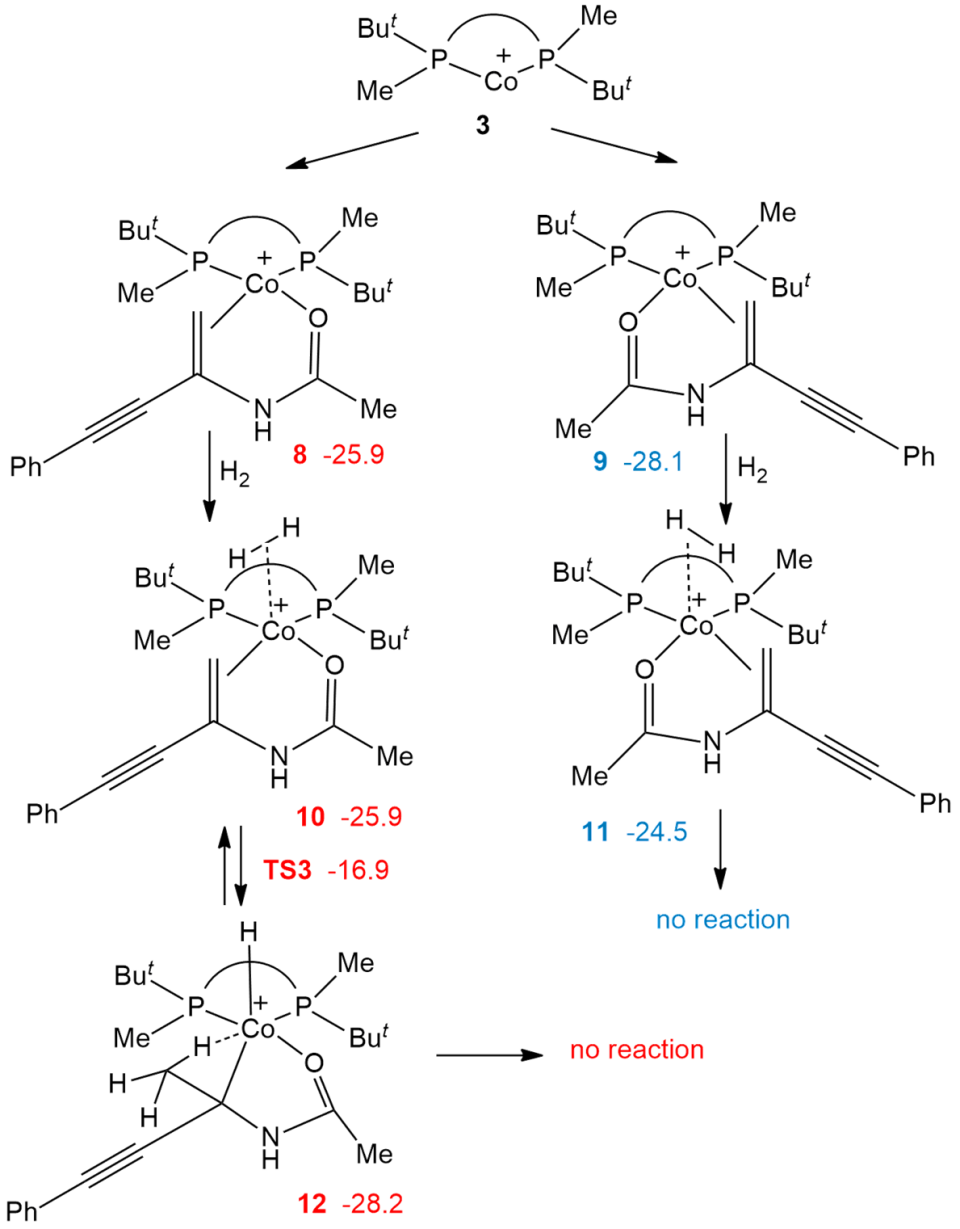
Reaction of the catalyst–substrate complexes **8** and **9** with dihydrogen. Computed free energies are given relative to **3** + **1** +H_2_ (shown in red and blue for *R*- and *S*-pathways, respectively).

Stable catalyst–substrate complexes **8** and **9** are formed upon the reaction of **3** with the substrate **1** (Scheme 3). They give corresponding molecular hydrogen complexes **10** and **11** upon reaction with dihydrogen. However, oxidative addition of H_2_ takes place only in complex **10**, whereas when a hydrogen atom approaches Co in complex **11**, a steady increase in energy results, and this does not lead to any productive transformation.

This could be a basis for a perfectly enantioselective reaction; however, the complex **12** is not capable of undergoing reductive elimination for the completion of the catalytic cycle. An extensive search for possible pathways of the reductive elimination in **12** invariably led to a reverse reaction, an exchange between two hydrides or dissociation of the double bond.

The same situation has previously been described for Rh-catalyzed asymmetric hydrogenations, where it was shown that dissociation of the double bond with its further re-association in the octahedral complexes is the most feasible pathway for reaction products [11,12].

Thus, the reaction of catalyst–substrate complexes with dihydrogen is unlikely to contribute to the flux of catalysis in the reaction under study.

### 2.3. Stereoselective Formation of Chelating Dihydrides—Four Competing Catalytic Cycles

**Scheme 4:**
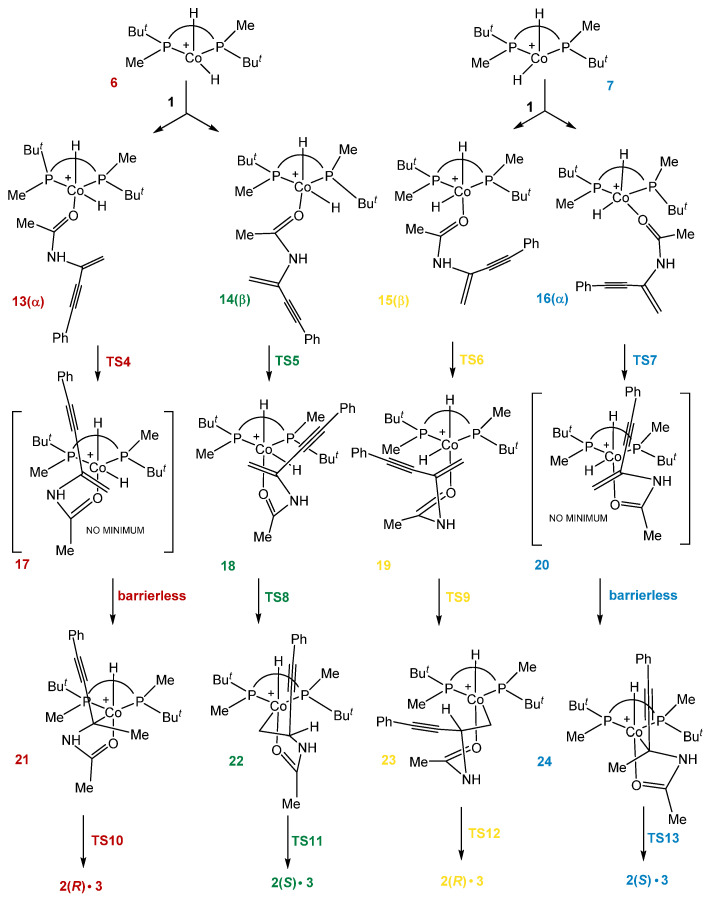
Four competing pathways in the Co(I)-Co(III) mechanism of the asymmetric hydrogenation of **1** with Co complex **3**.

As can be seen from the Scheme 3, the dihydride complexes **6** and **7** are stable and readily available intermediates. Therefore, their reactions with substrate **1** were studied (Scheme 4). Initially, encounter complexes **13**–**16** are formed. In these intermediates, the substrate is bound to the catalyst via coordination of the oxygen atom of the acetamido group to Co and through the non-covalent interactions of the phenylpropinyl group with the *t*-Bu and Me substituents of the ligand. Further approach of the double bond to the Co-H unit can proceed, producing α- or β- dihydride intermediates in each case, but only the β-dihydrides **18** and **19** could be located as definite minima. On the other hand, when the double bond in **13** and **16** achieved a proper orientation for the hydride transfer (coplanar to the Co-H bond *trans*- to phosphorus), immediate barrierless migratory insertion takes place yielding the corresponding monohydride intermediates **21** and **24**. Finally, the reductive elimination yields the product and recovers the catalyst in each of the four pathways (Scheme 4).

**Figure 1:**
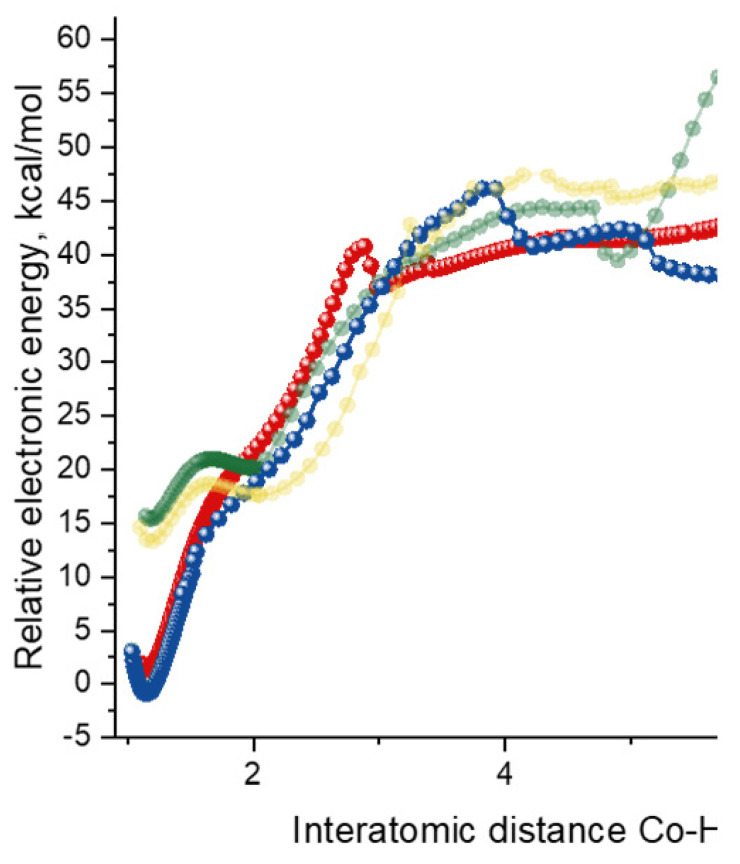
Scans of the electronic energy changes upon approach of the double bond towards the appropriate configuration for the migratory insertion step. Red—*R*(α), blue—*S*(α), yellow—*R*(β), green—*S*(β).

Scanning the approach of the double bond to the reactive site (Figure 1) revealed further details of the process of stereoselection during the formation of chelate cycles. Apparently, the discrimination between the α- and β-pathways takes place at the early stage of this process. Thus, in the range of the interatomic differences 6–8 Å, the interconversion between the different pathways is facile through the rotations around ordinary bonds, and the Boltzmann distribution strongly favors the α-pathways. Since at shorter interatomic distances the direct convergence between different pathways becomes impossible, the β-pathways can be excluded from the consideration.

**Figure 2:**
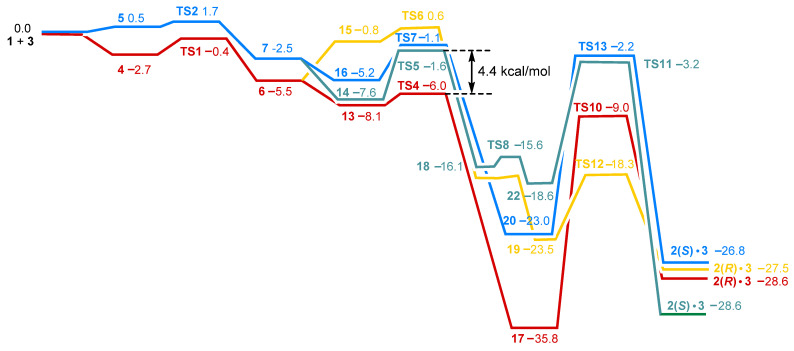
Profile of relative free energies (kcal/mol) in four competing catalytic cycles of the Co(I)-Co(III) route. Red—*R*(α), blue—*S*(α), yellow—*R*(β), green—*S*(β).

Both α-pathways exhibit definite maxima at around 3 Å (*R*(α) pathway) and around 4 Å (*S*(α) pathway), due to the necessity of changing the pattern of the disperse interactions between the substrate and the catalyst. The difference between the free energies of the corresponding transition states **TS4** and **TS7** apparently determines the optical yield of the whole catalytic cycle (Figure 2), since there are no other maxima on the way towards the corresponding monohydride intermediates **21** and **24** and the barrier of the reductive elimination of the *S*(α) pathway is significantly higher than that of the *R*(α) pathway.

**Figure 3:**
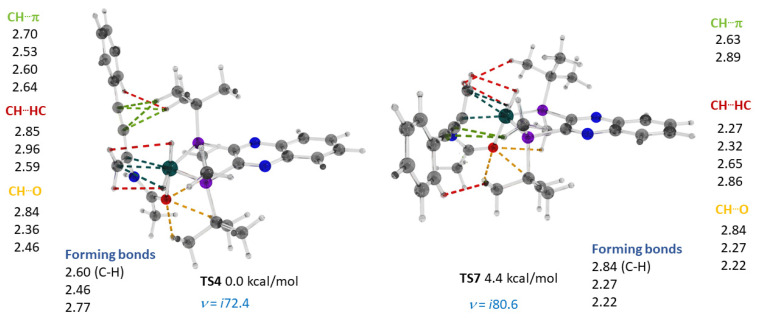
Optimized structures and important interatomic distances of the transition states **TS4** and **TS7**. Black—C, grey—H, blue—N, red—O, violet—P, turquoise—Co.

The optimized structures of **TS4** and **TS7** are shown in Figure 3. Comparing them, it can be concluded that the main contribution to the greater stability of the former originates from much more pronounced C-H^…^π interactions of the acetylenic unit with the *t*-Bu group in the **TS4** compared to the same interactions of the Me group in the **TS7**, since all other stabilizing intramolecular interactions appear to be very similar in both structures. These observations promote the triple bond as an effective stereoregulating group.

### 2.4. Co(0)-Co(II) Mechanism

The reaction conditions applied in the experimental study suggest that a Co(0)-Co(II) mechanism is also conceivable. This consideration prompted computations of the corresponding catalytic cycles.

**Scheme 5:**
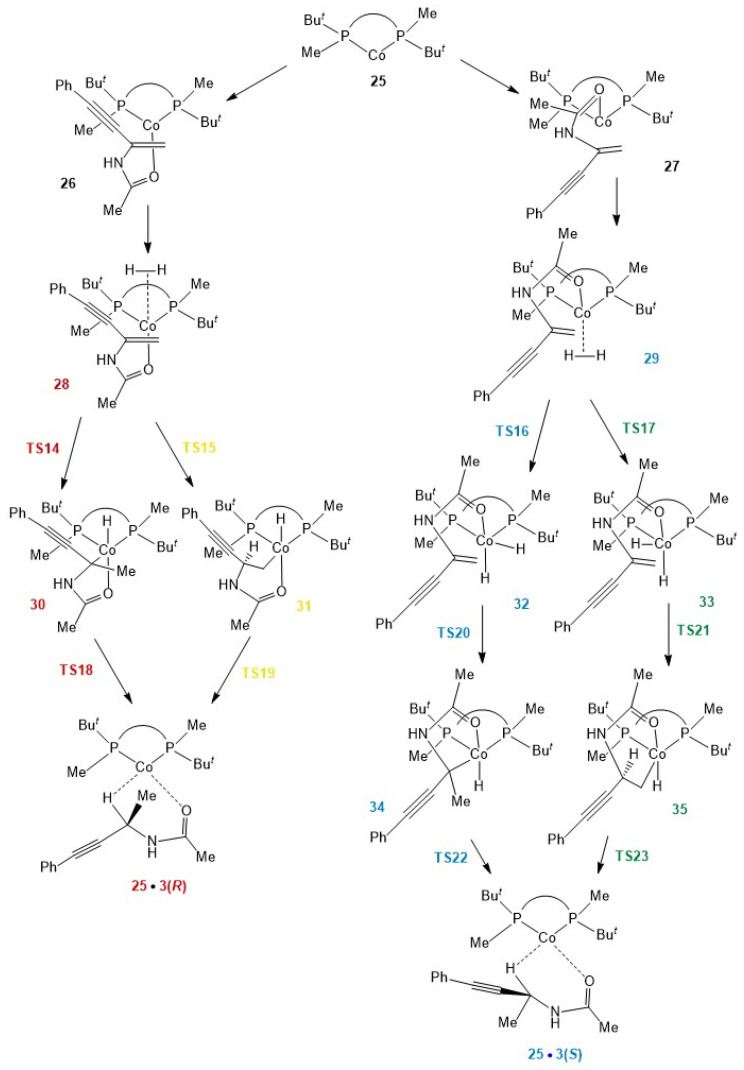
Four competing pathways of Co(0)-Co(II) catalytic cycles proceeding via hydrogenation of catalyst–substrate complexes.

The substrate can coordinate to the neutral solvate complex of Co(0) **25** with either of its prochiral planes exogonically yielding corresponding catalyst–substrate complexes **26** and **27** (Scheme 5). Endogonic coordination of molecular hydrogen giving complexes **28** and **29** is followed by oxidative addition to either the α- or β-carbon atom of the double bond via transition states **TS14-TS17**.

In the case of an *si*-coordinated olefin, we failed to locate intermediate dihydrides, since the migratory insertion proceeded spontaneously, resulting in corresponding monohydrides **30** and **31**, which provided *R*-product coordinated to the catalyst via the corresponding transition states **TS18** and **TS19**. The corresponding dihydrides **32** and **33**, originating from the *re*-coordination of the double bond, were located together with the transition states for migratory insertion **TS20** and **TS21**. Nevertheless, the relative free energies of **TS20** and **TS21** are notably lower than those of **TS16** and **TS17**, and the presence of the additional step in the *S*-catalytic cycles does not affect the process of enantioselection (vide infra).

**Figure 4:**
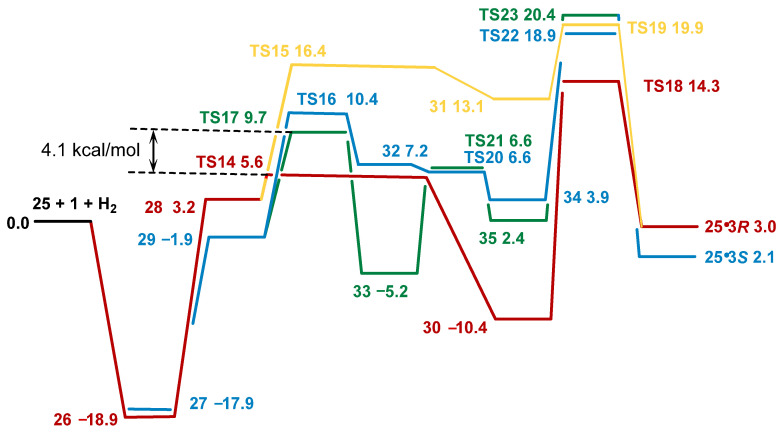
Profile of relative free energies (kcal/mol) in four competing catalytic cycles of the Ni(0)-Ni(II) route. Red—*R*(α), blue—*S*(α), yellow—*R*(β), green—*S*(β)

**Figure 5:**
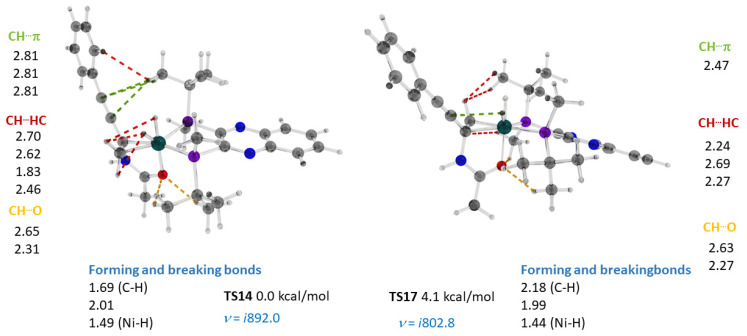
Optimized structures and important interatomic distances of the transition states **TS4** and **TS7**. Black—C, grey—H, blue—N, red—O, violet—P, turquoise—Co.

Inspection of Figure 4 makes it possible to conclude that the chirality is generated in the migratory insertion step via the difference in the free energies of **TS14**(*R*) and **TS17**(*S*) (Figure 5). Although in this case the reductive elimination becomes the rate-limiting stage, it does not affect the stereochemical outcome, since the difference in the free energies of the corresponding transition states (**TS18**(*R*) and either **TS22**(*S*) or **TS23**(*S*)) remains almost the same and for exactly the same reasons.

It is not easy to make a clear statement on which of the computed mechanisms is actually operating. The catalytic cycles are quite similar energetically (Figure 2 and Figure 4). Each of them comprises a final stage with activation barriers around 25 kcal/mol (reductive elimination in the case of the Co(I)-Co(III) mechanism and H_2_ metathesis in the case of Co(0)-Co((II) mechanism). Most probably, these two mechanisms can operate simultaneously in a manner that, in accord with the computational results, does not affect the almost perfect enantioselectivity observed in this reaction.

In either the Co(I)-Co(III) or the Co(0)-Co(II) mechanism, the *R*-enantioselectivity is secured via C-H^…^π interactions between the triple bond of the substrate and the *t*-Bu group of the catalyst. This means that the handedness of the asymmetric hydrogenation can be predicted without the need for detailed studies on the catalytic cycle, with the only reasonable assumption of the hydride coming from the side of the metal.

Using the well-known quadrant diagrams introduced by Knowles [55], but bearing in mind that the *t*-Bu group, which is rich in C-H bonds, actually provides a stabilizing effect, although not repulsion, as previously thought [56], one can predict the handedness of the product by positioning a substrate with groups capable of rendering disperse interactions against the “bulky quadrants” of the catalyst and determining the sign of chirality in a typical way, suggesting that the hydrogen atom will come from the side of the metal.

**Figure 6:**
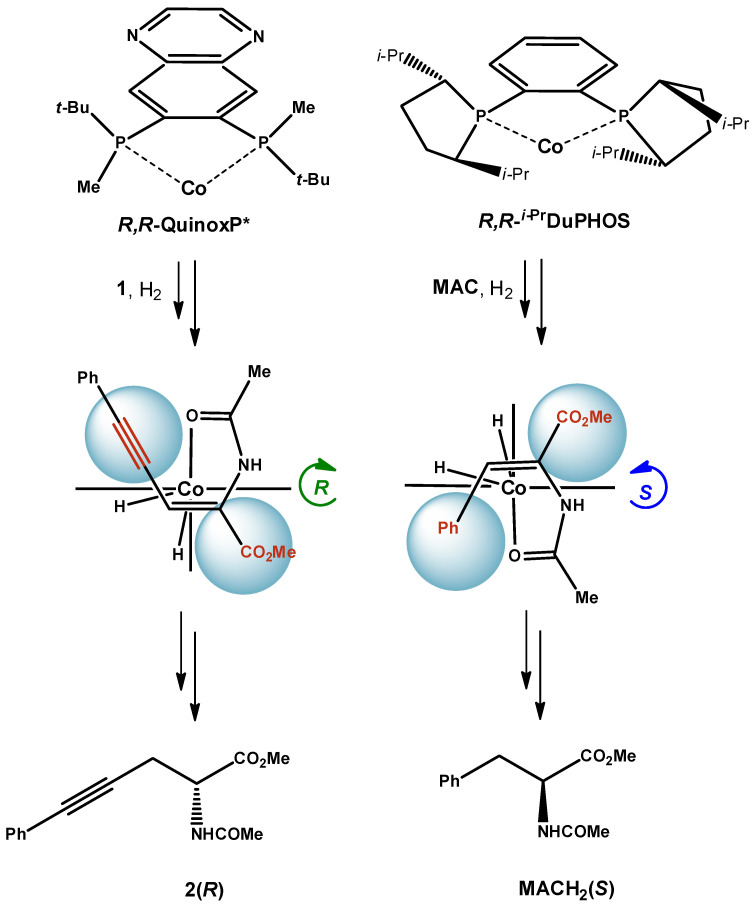
General scheme for predicting the handedness of asymmetric hydrogenation.

This approach is exemplified in Figure 6. *R*,*R*-QuinoxP* has *t*-Bu groups in the upper left and lower right quadrants. The *R*-configuration of the hydrogenation product can be predicted without detailed mechanistic studies, considering only the coordination of the substrate in ***an octahedral complex***, upon which the groups capable for building disperse interactions with *t*-Bu substituents would be positioned in the same quadrants (Figure 6, left). In the same fashion, the *S*-configuration of the **MACH_2_ [35]** can be predicted on the basis of the asymmetric hydrogenation with the *R*,*R^i^*^-Pr^DuPHOS-Rh complex.

The same predictions are valid for numerous Rh-catalyzed asymmetric hydrogenations [7,8,11,12,33], with the notable exception of enamides with *t*-Bu or adamantyl substituent [57,58] that choose a β-coordination pathway [11,12,56,59].

## 3. Conclusions

The results reported in this paper suggest that the focus of the studies of the mechanisms of asymmetric catalytic hydrogenations could be somewhat different from the commonly accepted one. The catalytic activity important for breaking chemical bonds and forming new ones is certainly quite sensitive to the electronic states of transition metal atoms On the other hand, the sense and level of enantioselection seem to be much less susceptible to redox processes. These considerations can provide an efficient and short way of arriving at important conclusions when only stereochemical information is required.

The author plans to study the further implications of the regularities reported in this paper.

## 4. Materials and Methods

### Computational Details

Geometry optimizations were performed without any symmetry constraints (C1 symmetry) using the ωB97XD functional [60] as implemented in the Gaussian09 software package [61]. Frequency calculations were undertaken to confirm the nature of the stationary points, yielding one imaginary frequency for all transition states (TS) and zero for all minima. Constrained energy hypersurface scans were conducted to investigate the molecular reactivity and to locate viable reaction channels. Where low-lying barriers were estimated, frequency calculations were performed at the crude saddle points, and the obtained force constants were used to optimize the transition state structures by employing the Berny algorithm [62]. All atoms were described using the 6-31G** basis set for geometry optimization and frequency calculation [63,64,65,66,67,68]. In the calculation of single-point energies, all atoms were described using the 6-311++G** basis set [69,70,71,72,73]. Non-specific solvation was introduced using the SMD continuum model [74] (acetonitrile). Cartesian coordinates of all optimized intermediates and transition states are given in the Supporting Information.

## Data Availability

Not applicable.

## Supplementary Materials

The following supporting information can be downloaded at: https://www.mdpi.com/article/10.3390/ijms24065568/s1.
