# Supplementary material for: Co-Catalyzed Asymmetric Hydrogenation. The Same Enantioselection Pattern for Different Mechanisms
Source: Int J Mol Sci. 2023 Mar 14;24(6):5568. doi: 10.3390/ijms24065568 (PMC10057697; doi:10.3390/ijms24065568)
Supplement: Supplementary file 1 [file ijms-24-05568-s001.zip › ijms-2260169-supplementary.pdf]

1

E(RwB97XD/6-311G++(d, p)) = -593.785917 a.u.

ΔG(RwB97XD/6-31G(d, p)) = 0.157741 a. u.

Input orientation:

Center Atomic Atomic Coordinates (Angstroms)
Number Number Type X Y Z
----
1 6 0 -0.788950 -0.382334 0.010020
2 6 0 0.397811 -0.622680 0.017765
3 6 0 1.813986 -0.860298 0.026694
4 6 0 2.303842 -2.108968 0.102839
5 1 0 3.366544 -2.299936 0.111282
6 1 0 1.607233 -2.937001 0.157776
7 6 0 -2.191385 -0.092706 0.003660
8 6 0 -2.631303 1.236291 0.100977
9 6 0 -3.132138 -1.128072 -0.100541
10 6 0 -3.991636 1.519942 0.095105
11 1 0 -1.902208 2.036115 0.180471
12 6 0 -4.490562 -0.833359 -0.105202
13 1 0 -2.791538 -2.155547 -0.177087
14 6 0 -4.923031 0.488167 -0.007811
15 1 0 -4.325572 2.549798 0.171976
16 1 0 -5.214765 -1.637832 -0.184743
17 1 0 -5.984972 0.712872 -0.011716
18 7 0 2.563188 0.328571 -0.050006
19 6 0 3.928745 0.460112 -0.031340
20 1 0 2.024934 1.182124 -0.110570
21 8 0 4.693546 -0.490945 0.040080
22 6 0 4.400399 1.894769 -0.085288
23 1 0 3.888410 2.453594 -0.873495
24 1 0 4.191342 2.388836 0.868971
25 1 0 5.475245 1.909341 -0.264725

2

E(RwB97XD/6-311G++(d, p)) = -595.021670 a.u.

ΔG(RwB97XD/6-31G(d, p)) = 0.181601 a. u.

Standard orientation:

Center Atomic Atomic Coordinates (Angstroms)
Number Number Type X Y Z

----
1 8 0 4.056076 -0.852471 -1.046832
2 6 0 3.699607 -0.693011 0.117641
3 7 0 2.633287 0.074935 0.459527
4 6 0 1.862454 0.821699 -0.522553
5 6 0 2.135026 2.326553 -0.422918
6 1 0 1.569426 2.870290 -1.184277
7 1 0 2.407959 0.204849 1.435787
8 6 0 4.434662 -1.331322 1.273836
9 1 0 4.590184 -2.389805 1.053781
10 1 0 5.418904 -0.862281 1.363793
11 1 0 3.910115 -1.232113 2.226493
12 1 0 1.848268 2.705974 0.562236
13 6 0 0.428014 0.523765 -0.383171
14 6 0 -0.754020 0.302743 -0.251475
15 6 0 -2.155660 0.035975 -0.094290
16 6 0 -2.950659 0.880580 0.694510
17 6 0 -2.742223 -1.071804 -0.724040
18 6 0 -4.307028 0.618190 0.849264
19 1 0 -2.498323 1.738609 1.181307
20 6 0 -4.099522 -1.326693 -0.563833
21 1 0 -2.128594 -1.725547 -1.335451
22 6 0 -4.884116 -0.484779 0.222356
23 1 0 -4.915364 1.275544 1.462391
24 1 0 -4.545887 -2.186721 -1.052975
25 1 0 -5.943206 -0.687445 0.346784
26 1 0 2.204496 0.459732 -1.496874
27 1 0 3.201182 2.512769 -0.576993

3

E(RwB97XD/6-311G++(d, p)) = -2877.618265 a.u.

ΔG(RwB97XD/6-31G(d, p)) = 0.377429 a. u.

Standard orientation:

Center Atomic Atomic Coordinates (Angstroms)
Number Number Type X Y Z
----
1 6 0 -0.542431 0.608368 0.258312
2 6 0 -0.541208 -0.697993 -0.312984
3 6 0 -2.818490 -0.692945 -0.325483
4 6 0 -2.820083 0.587800 0.291026
5 15 0 1.111631 1.381733 0.477036
6 15 0 1.112884 -1.454703 -0.574026
7 6 0 1.120934 1.735218 2.269475
8 1 0 1.996702 2.337153 2.527359

9 1 0 0.215787 2.260011 2.585636
10 1 0 1.186147 0.783862 2.804206
11 6 0 0.927631 -2.194371 -2.231783
12 6 0 1.003757 3.032983 -0.384512
13 6 0 0.461120 2.788710 -1.799082
14 1 0 1.041004 2.027896 -2.333979
15 1 0 -0.585637 2.469643 -1.781509
16 1 0 0.516712 3.720467 -2.372969
17 6 0 2.450958 3.544648 -0.462296
18 1 0 2.476369 4.513607 -0.973360
19 1 0 2.885591 3.680021 0.534423
20 1 0 3.087878 2.850527 -1.022010
21 6 0 0.128319 4.055208 0.346797
22 1 0 0.535462 4.303974 1.331521
23 1 0 0.090205 4.981340 -0.239144
24 1 0 -0.893429 3.689263 0.476755
25 6 0 1.176756 -2.876889 0.635166
26 6 0 2.575364 -3.496292 0.492279
27 1 0 2.728555 -3.925112 -0.503687
28 1 0 3.364010 -2.756899 0.673156
29 1 0 2.696069 -4.302709 1.224484
30 6 0 0.108060 -3.945540 0.385987
31 1 0 0.196767 -4.726067 1.150905
32 1 0 -0.902307 -3.531285 0.440105
33 1 0 0.231461 -4.423484 -0.590764
34 6 0 1.010657 -2.283077 2.040970
35 1 0 0.033904 -1.804165 2.168566
36 1 0 1.088952 -3.082077 2.786838
37 1 0 1.787088 -1.541391 2.257852
38 1 0 1.795382 -2.817301 -2.464621
39 1 0 0.020148 -2.798617 -2.303824
40 1 0 0.874714 -1.385257 -2.965219
41 27 0 2.578745 0.069004 -0.313853
42 7 0 -1.652823 1.230787 0.575029
43 7 0 -1.649928 -1.331511 -0.612421
44 6 0 -4.049383 -1.323612 -0.630129
45 1 0 -4.025462 -2.300925 -1.100922
46 6 0 -5.229398 -0.695494 -0.324692
47 1 0 -6.174672 -1.176592 -0.553549
48 6 0 -5.231192 0.580247 0.293963
49 6 0 -4.053184 1.213342 0.597443
50 1 0 -4.033133 2.191097 1.067479
51 1 0 -6.178000 1.057652 0.524146

4

E(RwB97XD/6-311G++(d, p)) = -2878.81170030 a. u.

∆G(RwB97XD/6-31G(d, p)) = 0.395522 a. u.

Standard orientation:

Center Atomic Atomic Coordinates (Angstroms)
Number Number Type X Y Z
----
1 6 0 -0.546793 -0.718937 -0.303356
2 6 0 -0.606236 0.587204 0.266513
3 6 0 -2.883132 0.474465 0.274043
4 6 0 -2.823346 -0.805438 -0.342938
5 15 0 1.127868 -1.431782 -0.557615
6 15 0 0.998330 1.451620 0.523181
7 6 0 1.003786 -2.177411 -2.208552
8 1 0 1.918465 -2.731987 -2.431369
9 1 0 0.142354 -2.845072 -2.281236
10 1 0 0.894094 -1.374441 -2.942138
11 6 0 0.948350 1.842897 2.306776
12 6 0 1.275793 -2.805769 0.689706
13 6 0 1.165243 -2.161763 2.079484
14 1 0 1.937642 -1.398684 2.229510
15 1 0 0.185766 -1.697684 2.235886
16 1 0 1.297345 -2.930558 2.848623
17 6 0 2.672476 -3.418374 0.503677
18 1 0 2.819611 -4.212000 1.244518
19 1 0 2.791838 -3.862064 -0.489918
20 1 0 3.463388 -2.673712 0.643471
21 6 0 0.205721 -3.887888 0.515691
22 1 0 0.281211 -4.378690 -0.459358
23 1 0 0.347302 -4.654314 1.286588
24 1 0 -0.804594 -3.484147 0.621392
25 6 0 0.841605 3.081921 -0.376650
26 6 0 2.257648 3.678705 -0.397951
27 1 0 2.647612 3.832851 0.614224
28 1 0 2.954172 3.029010 -0.939865
29 1 0 2.238800 4.651279 -0.902051
30 6 0 -0.126117 4.055790 0.303078
31 1 0 -0.193060 4.970092 -0.298135
32 1 0 -1.129067 3.631187 0.395169
33 1 0 0.222005 4.341727 1.300340
34 6 0 0.378933 2.787266 -1.809559
35 1 0 -0.647483 2.408109 -1.835334
36 1 0 0.408419 3.712860 -2.394859
37 1 0 1.028912 2.055069 -2.301294
38 1 0 1.772257 2.517256 2.555118
39 1 0 0.001509 2.305776 2.595298
40 1 0 1.078891 0.914313 2.868481
41 27 0 2.580118 0.124988 -0.286865
42 7 0 -1.627969 -1.393964 -0.616752
43 7 0 -1.747049 1.161734 0.569484
44 6 0 -4.143507 1.051102 0.567723

45 1 0 -4.167432 2.028492 1.038235
46 6 0 -5.290754 0.371538 0.250289
47 1 0 -6.258428 0.810602 0.469483
48 6 0 -5.231152 -0.903439 -0.369121
49 6 0 -4.025096 -1.484965 -0.661880
50 1 0 -3.957153 -2.459934 -1.132956
51 1 0 -6.154799 -1.419739 -0.609168
52 1 0 2.989230 0.041654 -1.741155
53 1 0 3.519630 -0.575342 -1.316578

TS1

E(RwB97XD/6-311G++(d, p)) = -2878.808896 a. u.

ΔG(RwB97XD/6-31G(d, p)) = 0.392331 a. u.

ν = *i*695.13

Standard orientation:

Center Atomic Atomic Coordinates (Angstroms)
Number Number Type X Y Z
----
1 6 0 -0.547715 -0.709693 -0.307975
2 6 0 -0.605998 0.592906 0.269053
3 6 0 -2.882909 0.480972 0.278374
4 6 0 -2.824205 -0.795797 -0.345818
5 15 0 1.123226 -1.432053 -0.562818
6 15 0 0.996039 1.456944 0.533519
7 6 0 1.007842 -2.162749 -2.218696
8 1 0 1.911852 -2.738117 -2.430947
9 1 0 0.131034 -2.808524 -2.304514
10 1 0 0.927836 -1.352871 -2.948312
11 6 0 0.935758 1.865838 2.311496
12 6 0 1.265904 -2.808114 0.682011
13 6 0 1.163141 -2.162899 2.072018
14 1 0 1.937328 -1.400325 2.217003
15 1 0 0.184952 -1.697871 2.233172
16 1 0 1.298960 -2.931266 2.840772
17 6 0 2.656625 -3.433424 0.493854
18 1 0 2.794359 -4.230050 1.233136
19 1 0 2.771643 -3.876108 -0.500588
20 1 0 3.454736 -2.697219 0.635799
21 6 0 0.185799 -3.880123 0.506768
22 1 0 0.254420 -4.367018 -0.470740
23 1 0 0.324435 -4.650476 1.274163
24 1 0 -0.820776 -3.468921 0.617990
25 6 0 0.854819 3.073007 -0.392501
26 6 0 2.271100 3.669522 -0.400631

27 1 0 2.644764 3.838560 0.615216
28 1 0 2.976102 3.012660 -0.922296
29 1 0 2.259611 4.634728 -0.918865
30 6 0 -0.123715 4.055179 0.259404
31 1 0 -0.184460 4.959465 -0.357314
32 1 0 -1.127113 3.630089 0.344921
33 1 0 0.210619 4.357715 1.256484
34 6 0 0.412767 2.757333 -1.827590
35 1 0 -0.612697 2.376226 -1.862727
36 1 0 0.448590 3.675298 -2.424411
37 1 0 1.071336 2.020600 -2.300360
38 1 0 1.760848 2.539326 2.558030
39 1 0 -0.011317 2.335837 2.587179
40 1 0 1.058023 0.942392 2.883500
41 27 0 2.589314 0.104849 -0.260180
42 7 0 -1.629485 -1.382322 -0.624862
43 7 0 -1.746682 1.166244 0.575626
44 6 0 -4.142719 1.055795 0.578312
45 1 0 -4.165645 2.030679 1.053980
46 6 0 -5.290279 0.377314 0.260388
47 1 0 -6.257581 0.814651 0.484550
48 6 0 -5.231811 -0.894642 -0.365716
49 6 0 -4.026588 -1.474251 -0.665099
50 1 0 -3.959669 -2.446891 -1.141027
51 1 0 -6.155998 -1.410095 -0.605428
52 1 0 2.853341 0.163312 -1.698262
53 1 0 3.486292 -0.584544 -1.267037

6

E(RwB97XD/6-311G++(d, p)) = -2878.816208 a. u.

∆G(RwB97XD/6-31G(d, p)) = 0.395522 a. u.

Standard orientation:

Center Atomic Atomic Coordinates (Angstroms)
Number Number Type X Y Z
----
1 6 0 -0.518978 -0.732760 -0.313046
2 6 0 -0.608670 0.569468 0.258330
3 6 0 -2.881640 0.395552 0.281328
4 6 0 -2.791547 -0.881924 -0.339195
5 15 0 1.162943 -1.425928 -0.584162
6 15 0 0.962310 1.489157 0.500052
7 6 0 1.039106 -2.201453 -2.214236
8 1 0 1.981762 -2.693240 -2.461529
9 1 0 0.226275 -2.931061 -2.233036
10 1 0 0.838982 -1.422195 -2.953199

11 6 0 0.976360 1.805791 2.295375
12 6 0 1.383281 -2.744704 0.711108
13 6 0 1.454263 -2.019303 2.062826
14 1 0 2.281307 -1.300134 2.090058
15 1 0 0.524316 -1.483451 2.280911
16 1 0 1.616862 -2.751358 2.860875
17 6 0 2.712254 -3.457790 0.421555
18 1 0 2.890965 -4.204279 1.202869
19 1 0 2.690735 -3.979478 -0.540025
20 1 0 3.555177 -2.760592 0.418134
21 6 0 0.236511 -3.760793 0.710410
22 1 0 0.138862 -4.266387 -0.254898
23 1 0 0.446844 -4.523931 1.468131
24 1 0 -0.722191 -3.297624 0.955663
25 6 0 0.738551 3.129015 -0.357482
26 6 0 2.147744 3.729666 -0.476293
27 1 0 2.615921 3.860168 0.505420
28 1 0 2.798741 3.096723 -1.089824
29 1 0 2.085202 4.714761 -0.951425
30 6 0 -0.172839 4.075767 0.431637
31 1 0 -0.288576 5.008548 -0.131848
32 1 0 -1.164903 3.643495 0.586753
33 1 0 0.254219 4.329527 1.406236
34 6 0 0.155718 2.882059 -1.754783
35 1 0 -0.865956 2.493771 -1.705896
36 1 0 0.124592 3.833513 -2.296608
37 1 0 0.765953 2.184205 -2.337004
38 1 0 1.796395 2.485524 2.540827
39 1 0 0.032111 2.238835 2.633870
40 1 0 1.141419 0.857729 2.813103
41 27 0 2.559253 0.152046 -0.359460
42 7 0 -1.583193 -1.435849 -0.622741
43 7 0 -1.763477 1.113227 0.566895
44 6 0 -4.154950 0.937375 0.586110
45 1 0 -4.202201 1.912811 1.058741
46 6 0 -5.284766 0.227025 0.275209
47 1 0 -6.262597 0.638345 0.502745
48 6 0 -5.195069 -1.044799 -0.348156
49 6 0 -3.976701 -1.593285 -0.651872
50 1 0 -3.885899 -2.564973 -1.125665
51 1 0 -6.106625 -1.584413 -0.582924
52 1 0 2.113296 0.551783 -1.664141
53 1 0 3.475313 -0.771387 -1.111279

5

E(RwB97XD/6-311G++(d, p)) = -2878.80909233 a. u.

∆G(RwB97XD/6-31G(d, p)) = 0.394836 a. u.

Standard orientation:

Center Atomic Atomic Coordinates (Angstroms)
Number Number Type X Y Z
----
1 6 0 -0.601120 0.581584 0.267502
2 6 0 -0.520158 -0.726472 -0.295742
3 6 0 -2.796429 -0.850294 -0.327544
4 6 0 -2.876044 0.428751 0.288410
5 15 0 0.989177 1.483653 0.471857
6 15 0 1.167074 -1.418164 -0.560970
7 6 0 1.047975 1.779679 2.271808
8 1 0 1.894337 2.429070 2.510173
9 1 0 0.123499 2.237540 2.632932
10 1 0 1.193609 0.819844 2.773565
11 6 0 1.026567 -2.154482 -2.218836
12 6 0 0.739995 3.142635 -0.343424
13 6 0 0.212410 2.882371 -1.760514
14 1 0 0.846026 2.174452 -2.308067
15 1 0 -0.808584 2.489020 -1.747639
16 1 0 0.201285 3.823125 -2.321766
17 6 0 2.140200 3.772968 -0.418480
18 1 0 2.074248 4.755999 -0.897849
19 1 0 2.574424 3.913988 0.577219
20 1 0 2.826022 3.153306 -1.007520
21 6 0 -0.209414 4.069379 0.421188
22 1 0 0.183737 4.318205 1.411629
23 1 0 -0.321553 5.007286 -0.135219
24 1 0 -1.198439 3.620989 0.543620
25 6 0 1.303494 -2.812406 0.670828
26 6 0 2.688365 -3.456818 0.509654
27 1 0 2.845879 -3.838143 -0.504490
28 1 0 3.495673 -2.760021 0.748139
29 1 0 2.764313 -4.304368 1.199570
30 6 0 0.224575 -3.881994 0.458359
31 1 0 0.343062 -4.656434 1.224788
32 1 0 -0.784944 -3.473218 0.544148
33 1 0 0.319899 -4.364540 -0.519203
34 6 0 1.169385 -2.193738 2.069475
35 1 0 0.193820 -1.717155 2.212892
36 1 0 1.269318 -2.981240 2.824471
37 1 0 1.948630 -1.446506 2.252497
38 1 0 1.913593 -2.755229 -2.433741
39 1 0 0.131672 -2.775438 -2.301423
40 1 0 0.966935 -1.346542 -2.953384
41 27 0 2.573924 0.205246 -0.407431
42 7 0 -1.750603 1.136758 0.574378
43 7 0 -1.592235 -1.418387 -0.603725
44 6 0 -3.987213 -1.549470 -0.645540
45 1 0 -3.903807 -2.523195 -1.116769

46 6 0 -5.202030 -0.987458 -0.351275
47 1 0 -6.117660 -1.518155 -0.590703
48 6 0 -5.281495 0.286504 0.268243
49 6 0 -4.145191 0.984865 0.584223
50 1 0 -4.184790 1.962362 1.053468
51 1 0 -6.255995 0.709834 0.488093
52 1 0 3.646827 -0.618673 0.285059
53 1 0 3.775873 -0.782343 -0.591735

TS2

E(RwB97XD/6-311G++(d, p)) = - -2878.805383 a. u.

ΔG(RwB97XD/6-31G(d, p)) = 0.394836 a. u.

ν = *i*729.32

Standard orientation:

Center Atomic Atomic Coordinates (Angstroms)
Number Number Type X Y Z
----
1 6 0 0.591143 0.674352 -0.337905
2 6 0 0.516304 -0.634785 0.224313
3 6 0 2.795659 -0.731605 0.281359
4 6 0 2.868402 0.544051 -0.341564
5 15 0 -0.992637 1.573773 -0.572687
6 15 0 -1.165183 -1.360826 0.461799
7 6 0 -0.818060 2.261362 -2.251716
8 1 0 -1.640988 2.950264 -2.457901
9 1 0 0.134012 2.784810 -2.368322
10 1 0 -0.862981 1.436446 -2.967363
11 6 0 -1.210480 -1.655494 2.258339
12 6 0 -0.916363 3.009655 0.616100
13 6 0 -0.751107 2.418502 2.022722
14 1 0 -1.552277 1.708454 2.258789
15 1 0 0.209805 1.906811 2.140189
16 1 0 -0.791190 3.224520 2.763631
17 6 0 -2.277603 3.713424 0.504084
18 1 0 -2.321829 4.536909 1.225344
19 1 0 -2.435744 4.134544 -0.494079
20 1 0 -3.104317 3.028606 0.724150
21 6 0 0.211795 3.999397 0.314625
22 1 0 0.087533 4.466435 -0.667232
23 1 0 0.197931 4.796460 1.066889
24 1 0 1.193263 3.519404 0.345329
25 6 0 -1.117770 -3.023535 -0.381780
26 6 0 -2.519338 -3.648162 -0.297667
27 1 0 -2.869661 -3.728102 0.736981

28 1 0 -3.258752 -3.084727 -0.870476
29 1 0 -2.473612 -4.660841 -0.712825
30 6 0 -0.116312 -3.981806 0.279147
31 1 0 -0.114073 -4.922743 -0.282525
32 1 0 0.897827 -3.578637 0.282156
33 1 0 -0.400277 -4.212772 1.310541
34 6 0 -0.733287 -2.782039 -1.848090
35 1 0 0.282179 -2.383935 -1.942652
36 1 0 -0.770967 -3.733595 -2.389112
37 1 0 -1.423072 -2.086929 -2.338204
38 1 0 -2.096775 -2.240418 2.516133
39 1 0 -0.312510 -2.183267 2.588694
40 1 0 -1.264446 -0.691503 2.771037
41 27 0 -2.620386 0.089210 -0.193194
42 7 0 1.738379 1.241629 -0.631989
43 7 0 1.594670 -1.307227 0.552079
44 6 0 3.989889 -1.421720 0.606432
45 1 0 3.911926 -2.393354 1.082879
46 6 0 5.201437 -0.854487 0.309219
47 1 0 6.119936 -1.378616 0.552033
48 6 0 5.274084 0.415720 -0.319247
49 6 0 4.134359 1.105922 -0.640377
50 1 0 4.168547 2.080131 -1.116701
51 1 0 6.246415 0.842383 -0.542183
52 1 0 -3.068959 -0.737287 -1.311619
53 1 0 -3.637071 -0.995645 -0.452618

7

E(RwB97XD/6-311G++(d, p)) = -2878.813067 a. u.

ΔG(RwB97XD/6-31G(d, p)) = 0.394107 a. u.

Standard orientation:

Center Atomic Atomic Coordinates (Angstroms)
Number Number Type X Y Z
----
1 6 0 -0.578223 0.646367 0.306408
2 6 0 -0.521696 -0.654423 -0.272642
3 6 0 -2.799371 -0.730961 -0.322310
4 6 0 -2.856005 0.541456 0.311686
5 15 0 1.013079 1.526513 0.550063
6 15 0 1.144386 -1.407480 -0.515201
7 6 0 0.954092 2.020551 2.298088
8 1 0 1.785908 2.694697 2.517475
9 1 0 0.010848 2.518459 2.535525

10 1 0 1.055181 1.124986 2.915333
11 6 0 1.124885 -1.912091 -2.260208
12 6 0 0.908568 3.063292 -0.492780
13 6 0 0.591336 2.611968 -1.924739
14 1 0 1.312436 1.868455 -2.285110
15 1 0 -0.413294 2.184622 -2.004930
16 1 0 0.640370 3.475383 -2.597094
17 6 0 2.310098 3.692433 -0.437561
18 1 0 2.325876 4.605433 -1.042847
19 1 0 2.589625 3.964277 0.585704
20 1 0 3.071383 3.010316 -0.831948
21 6 0 -0.138218 4.063244 0.005252
22 1 0 0.108334 4.440230 1.002621
23 1 0 -0.166665 4.920023 -0.677789
24 1 0 -1.136589 3.620097 0.041594
25 6 0 1.140218 -2.947658 0.536691
26 6 0 2.558245 -3.533514 0.549616
27 1 0 2.930200 -3.719907 -0.463198
28 1 0 3.263041 -2.873190 1.059909
29 1 0 2.535356 -4.490795 1.081411
30 6 0 0.167497 -3.988768 -0.038217
31 1 0 0.142241 -4.848263 0.640498
32 1 0 -0.847517 -3.595811 -0.131478
33 1 0 0.494108 -4.348269 -1.018547
34 6 0 0.714197 -2.561078 1.959254
35 1 0 -0.314659 -2.189264 1.992462
36 1 0 0.766396 -3.450236 2.596500
37 1 0 1.374532 -1.801097 2.388496
38 1 0 1.970787 -2.574096 -2.459056
39 1 0 0.189741 -2.421797 -2.503506
40 1 0 1.217624 -1.019527 -2.884545
41 27 0 2.603940 0.078376 -0.092409
42 7 0 -1.718898 1.222164 0.610746
43 7 0 -1.605924 -1.317564 -0.600499
44 6 0 -4.002456 -1.404271 -0.650183
45 1 0 -3.937075 -2.373364 -1.133416
46 6 0 -5.206185 -0.824152 -0.347183
47 1 0 -6.131309 -1.335519 -0.591825
48 6 0 -5.262809 0.442956 0.289862
49 6 0 -4.114954 1.116869 0.615029
50 1 0 -4.136533 2.088029 1.098127
51 1 0 -6.229712 0.879918 0.516261
52 1 0 2.615567 -0.435517 1.246354
53 1 0 3.583882 -1.005435 -0.448946

8

E(RwB97XD/6-311G++(d, p)) = - 3471.482457 a. u.

ΔG(RwB97XD/6-31G(d, p)) = 0.569816 a. u.

Standard orientation:

Center Atomic Atomic Coordinates (Angstroms)
Number Number Type X Y Z
----
1 6 0 1.771748 -0.067044 0.554400
2 6 0 1.791513 -0.665915 -0.739995
3 6 0 3.872939 0.192946 -1.099108
4 6 0 3.809581 0.869372 0.150568
5 15 0 0.354295 -0.481516 1.653085
6 15 0 0.235197 -1.468197 -1.302488
7 6 0 0.023682 1.090620 2.502493
8 1 0 -0.819634 0.982577 3.187156
9 1 0 0.904533 1.422848 3.056543
10 1 0 -0.225784 1.839478 1.747848
11 6 0 0.789227 -3.038227 -2.042931
12 6 0 1.091943 -1.681737 2.893553
13 6 0 1.324972 -3.000154 2.139108
14 1 0 0.389624 -3.394295 1.718435
15 1 0 2.049074 -2.881949 1.325720
16 1 0 1.718841 -3.752201 2.831671
17 6 0 0.064499 -1.901576 4.013657
18 1 0 0.484052 -2.604557 4.741949
19 1 0 -0.167691 -0.972746 4.544176
20 1 0 -0.866429 -2.329694 3.634022
21 6 0 2.405703 -1.182880 3.502437
22 1 0 2.283242 -0.220753 4.008615
23 1 0 2.742545 -1.912542 4.247633
24 1 0 3.195758 -1.080984 2.755277
25 6 0 -0.294540 -0.356393 -2.720007
26 6 0 -1.745012 -0.708449 -3.075425
27 1 0 -1.872978 -1.777696 -3.276076
28 1 0 -2.432992 -0.416152 -2.278458
29 1 0 -2.032487 -0.163785 -3.981679
30 6 0 0.595155 -0.540167 -3.954873
31 1 0 0.294750 0.192255 -4.713016
32 1 0 1.652069 -0.379479 -3.726882
33 1 0 0.479536 -1.536012 -4.393406
34 6 0 -0.224156 1.097878 -2.231758
35 1 0 0.808483 1.441619 -2.119158
36 1 0 -0.710664 1.745811 -2.969270
37 1 0 -0.740220 1.231225 -1.275973
38 1 0 -0.038641 -3.484931 -2.599046
39 1 0 1.640506 -2.890737 -2.710923
40 1 0 1.074573 -3.722002 -1.239149
41 27 0 -1.159572 -1.626790 0.487015
42 7 0 2.743359 0.707822 0.978282
43 7 0 2.831693 -0.564223 -1.535708
44 6 0 5.022397 0.337061 -1.915435
45 1 0 5.051989 -0.188345 -2.864301

46 6 0 6.054217 1.136192 -1.497516
47 1 0 6.936006 1.252796 -2.118857
48 6 0 5.985404 1.821523 -0.256798
49 6 0 4.888283 1.695169 0.554535
50 1 0 4.818470 2.203457 1.510531
51 1 0 6.815770 2.449704 0.048454
52 8 0 -2.238095 -2.927920 -0.477684
53 6 0 -3.397064 -2.508231 -0.749941
54 6 0 -4.335565 -3.324396 -1.569483
55 1 0 -5.298874 -2.831080 -1.702002
56 1 0 -3.879732 -3.504333 -2.546625
57 1 0 -4.482993 -4.292105 -1.083508
58 7 0 -3.751137 -1.313119 -0.300160
59 1 0 -4.683650 -0.947997 -0.449455
60 6 0 -2.824962 -0.650969 0.603432
61 6 0 -2.795842 0.773490 0.482997
62 6 0 -2.692546 1.978738 0.402298
63 6 0 -2.556354 -1.334112 1.840390
64 1 0 -3.163001 -2.208510 2.074361
65 6 0 -2.507324 3.392173 0.285615
66 6 0 -2.636227 4.021052 -0.962178
67 6 0 -2.164899 4.154798 1.413005
68 6 0 -2.428425 5.390706 -1.074698
69 1 0 -2.897879 3.429774 -1.833870
70 6 0 -1.953897 5.523083 1.288387
71 1 0 -2.063225 3.667828 2.377526
72 6 0 -2.085934 6.143736 0.047462
73 1 0 -2.532005 5.871879 -2.041975
74 1 0 -1.686717 6.106413 2.163944
75 1 0 -1.921240 7.212459 -0.046146
76 1 0 -2.310638 -0.727953 2.706988

10

E(RwB97XD/6-311G++(d, p)) = --3472.671438 a. u.

ΔG(RwB97XD/6-31G(d, p)) = 0.586973 a. u.

Standard orientation:

Center Atomic Atomic Coordinates (Angstroms)
Number Number Type X Y Z
----
1 6 0 1.893235 0.252341 0.455177
2 6 0 1.937585 -0.315886 -0.850740
3 6 0 3.869504 0.819024 -1.276945
4 6 0 3.776555 1.451771 -0.006771
5 15 0 0.580478 -0.338213 1.607992
6 15 0 0.483442 -1.317410 -1.356671

7 6 0 -0.218581 1.216250 2.113073
8 1 0 -0.968679 1.019094 2.882116
9 1 0 0.526970 1.914846 2.498038
10 1 0 -0.704286 1.660195 1.243884
11 6 0 1.206269 -2.709869 -2.283692
12 6 0 1.550771 -0.940255 3.108194
13 6 0 2.697582 -1.845415 2.635017
14 1 0 2.352824 -2.676578 2.014223
15 1 0 3.451028 -1.285447 2.073774
16 1 0 3.189945 -2.273463 3.514747
17 6 0 0.605643 -1.727538 4.027469
18 1 0 1.159953 -2.024359 4.924760
19 1 0 -0.243406 -1.116805 4.350678
20 1 0 0.220040 -2.635691 3.557077
21 6 0 2.134772 0.236090 3.906500
22 1 0 1.350246 0.842655 4.367653
23 1 0 2.754518 -0.170922 4.713439
24 1 0 2.761020 0.881872 3.287068
25 6 0 -0.335876 -0.210831 -2.631796
26 6 0 -1.712650 -0.806456 -2.953267
27 1 0 -1.645452 -1.858954 -3.250853
28 1 0 -2.394974 -0.726637 -2.104683
29 1 0 -2.154871 -0.253944 -3.789817
30 6 0 0.489560 -0.116247 -3.920265
31 1 0 0.013395 0.609752 -4.589355
32 1 0 1.513134 0.217361 -3.730883
33 1 0 0.529510 -1.074717 -4.446379
34 6 0 -0.503402 1.180440 -2.006250
35 1 0 0.456390 1.694547 -1.894439
36 1 0 -1.137244 1.794701 -2.655469
37 1 0 -0.985750 1.124656 -1.025934
38 1 0 0.410919 -3.239533 -2.813759
39 1 0 1.957176 -2.367658 -2.998744
40 1 0 1.668534 -3.399380 -1.572717
41 27 0 -0.620845 -1.906552 0.532680
42 7 0 2.774350 1.139089 0.855358
43 7 0 2.915608 -0.057507 -1.689046
44 6 0 4.950986 1.134129 -2.137083
45 1 0 5.006510 0.638668 -3.100750
46 6 0 5.885195 2.055070 -1.740568
47 1 0 6.712250 2.304819 -2.396998
48 6 0 5.784122 2.696275 -0.478545
49 6 0 4.753473 2.404194 0.375660
50 1 0 4.660162 2.878470 1.347015
51 1 0 6.536202 3.423547 -0.191529
52 8 0 -1.448924 -3.370842 -0.472304
53 6 0 -2.679083 -3.211699 -0.703448
54 6 0 -3.443196 -4.212672 -1.499822
55 1 0 -4.471721 -3.895722 -1.675153
56 1 0 -2.936852 -4.367650 -2.455350

57 1 0 -3.445854 -5.163965 -0.961061
58 7 0 -3.270531 -2.131443 -0.221708
59 1 0 -4.258863 -1.965562 -0.366203
60 6 0 -2.500704 -1.274764 0.664207
61 6 0 -2.893769 0.097609 0.579167
62 6 0 -3.218528 1.257979 0.450042
63 6 0 -2.053139 -1.901832 1.877818
64 1 0 -2.459951 -2.883256 2.113939
65 6 0 -3.507966 2.649906 0.293032
66 6 0 -4.028573 3.132032 -0.917616
67 6 0 -3.241626 3.548957 1.337317
68 6 0 -4.279690 4.489830 -1.075255
69 1 0 -4.229302 2.436471 -1.726238
70 6 0 -3.491136 4.905824 1.167147
71 1 0 -2.835323 3.176071 2.272225
72 6 0 -4.011188 5.379541 -0.036117
73 1 0 -4.683839 4.856108 -2.013672
74 1 0 -3.278446 5.596425 1.977063
75 1 0 -4.205007 6.439627 -0.165068
76 1 0 -1.901628 -1.266095 2.744144
77 1 0 0.072832 -3.063595 1.423171
78 1 0 0.600440 -2.975746 0.804227

TS3

E(RwB97XD/6-311G++(d, p)) = -3472.655946 a. u.

ΔG(RwB97XD/6-31G(d, p)) = 0.585866 a. u.

ν = *i*729.58

Standard orientation:

Center Atomic Atomic Coordinates (Angstroms)
Number Number Type X Y Z
----
1 6 0 1.879659 0.430330 0.441889
2 6 0 2.055357 -0.391885 -0.706221
3 6 0 3.969918 0.731251 -1.218675
4 6 0 3.739547 1.616946 -0.129425
5 15 0 0.507302 -0.002818 1.593516
6 15 0 0.679288 -1.536366 -1.095551
7 6 0 -0.379021 1.577322 1.758405
8 1 0 -1.149708 1.493937 2.528104
9 1 0 0.317338 2.376957 2.018562
10 1 0 -0.853224 1.811493 0.804549
11 6 0 1.500604 -3.046490 -1.691924
12 6 0 1.386930 -0.283424 3.237869
13 6 0 2.539119 -1.274230 3.016863

14 1 0 2.196878 -2.216147 2.578112
15 1 0 3.318197 -0.856250 2.372119
16 1 0 2.997347 -1.502312 3.985108
17 6 0 0.391739 -0.855218 4.258812
18 1 0 0.893813 -0.919702 5.230235
19 1 0 -0.483526 -0.209406 4.381877
20 1 0 0.054627 -1.862050 4.000984
21 6 0 1.943175 1.034784 3.799811
22 1 0 1.142066 1.724339 4.081020
23 1 0 2.514511 0.806601 4.706540
24 1 0 2.606603 1.538124 3.094065
25 6 0 -0.102872 -0.723213 -2.596221
26 6 0 -1.401874 -1.472096 -2.920775
27 1 0 -1.234576 -2.545587 -3.058436
28 1 0 -2.154127 -1.328300 -2.142315
29 1 0 -1.814276 -1.078789 -3.856577
30 6 0 0.824184 -0.765138 -3.817103
31 1 0 0.347721 -0.215242 -4.636853
32 1 0 1.793643 -0.301788 -3.617606
33 1 0 0.993818 -1.789581 -4.161957
34 6 0 -0.425689 0.731428 -2.226997
35 1 0 0.480982 1.329453 -2.090792
36 1 0 -1.005083 1.186872 -3.037640
37 1 0 -1.026651 0.793018 -1.313940
38 1 0 0.762375 -3.703861 -2.156298
39 1 0 2.285353 -2.807589 -2.412741
40 1 0 1.941260 -3.562618 -0.835678
41 27 0 -0.560684 -1.784440 0.732368
42 7 0 2.680853 1.436242 0.703098
43 7 0 3.088955 -0.263176 -1.505641
44 6 0 5.112662 0.910684 -2.036980
45 1 0 5.272679 0.220430 -2.858657
46 6 0 5.974528 1.944123 -1.778294
47 1 0 6.850709 2.088813 -2.401612
48 6 0 5.735388 2.838178 -0.702363
49 6 0 4.641925 2.683393 0.108854
50 1 0 4.444721 3.351010 0.941125
51 1 0 6.433748 3.648798 -0.522619
52 8 0 -1.243840 -3.423958 -0.100530
53 6 0 -2.470340 -3.390012 -0.403365
54 6 0 -3.115123 -4.534453 -1.105601
55 1 0 -4.175206 -4.355751 -1.287604
56 1 0 -2.602491 -4.694167 -2.057861
57 1 0 -2.995139 -5.438630 -0.503975
58 7 0 -3.161182 -2.304707 -0.097099
59 1 0 -4.147420 -2.232888 -0.314641
60 6 0 -2.496821 -1.286282 0.691892
61 6 0 -2.973352 0.034263 0.442394
62 6 0 -3.360696 1.151177 0.172221
63 6 0 -2.067369 -1.741848 2.001784

64 1 0 -2.488084 -2.688008 2.340966
65 6 0 -3.733581 2.498435 -0.129508
66 6 0 -4.237314 2.831531 -1.396345
67 6 0 -3.571631 3.505509 0.835082
68 6 0 -4.577398 4.147936 -1.685714
69 1 0 -4.355693 2.055405 -2.145798
70 6 0 -3.909685 4.819733 0.533208
71 1 0 -3.177900 3.250535 1.813874
72 6 0 -4.414635 5.144407 -0.724615
73 1 0 -4.969035 4.396781 -2.667042
74 1 0 -3.780617 5.592573 1.284467
75 1 0 -4.678350 6.171672 -0.955397
76 1 0 -2.011372 -0.992522 2.785990
77 1 0 -0.561596 -2.465835 2.085808
78 1 0 0.301615 -2.691820 1.687120

12

E(RwB97XD/6-311G++(d, p)) = -3472.676483 a. u.

ΔG(RwB97XD/6-31G(d, p)) = 0.588259 a. u.

Standard orientation:

Center Atomic Atomic Coordinates (Angstroms)
Number Number Type X Y Z
----
1 6 0 1.919641 0.345605 0.460317
2 6 0 1.959307 -0.230838 -0.838807
3 6 0 3.879018 0.909274 -1.286517
4 6 0 3.795717 1.544359 -0.015908
5 15 0 0.617603 -0.252529 1.608251
6 15 0 0.519018 -1.256422 -1.326095
7 6 0 -0.275804 1.269317 2.062332
8 1 0 -1.017004 1.042865 2.832816
9 1 0 0.415705 2.028958 2.432382
10 1 0 -0.788044 1.648633 1.177125
11 6 0 1.238815 -2.662411 -2.221937
12 6 0 1.565803 -0.792604 3.143141
13 6 0 2.794256 -1.613320 2.727307
14 1 0 2.528301 -2.449006 2.073476
15 1 0 3.543745 -0.999028 2.220832
16 1 0 3.257570 -2.026586 3.629585
17 6 0 0.643108 -1.658547 4.012571
18 1 0 1.156133 -1.879362 4.954716
19 1 0 -0.291766 -1.144813 4.257591
20 1 0 0.407268 -2.613094 3.533097
21 6 0 2.018064 0.427781 3.960288
22 1 0 1.169117 0.970187 4.385562

23 1 0 2.635200 0.073873 4.793543
24 1 0 2.617424 1.118965 3.363267
25 6 0 -0.346749 -0.193885 -2.600960
26 6 0 -1.690663 -0.850680 -2.940782
27 1 0 -1.571801 -1.897388 -3.242433
28 1 0 -2.388047 -0.804756 -2.103326
29 1 0 -2.143423 -0.314137 -3.781772
30 6 0 0.488964 -0.067899 -3.882082
31 1 0 -0.022584 0.625466 -4.559178
32 1 0 1.490295 0.324440 -3.688340
33 1 0 0.585061 -1.026759 -4.399890
34 6 0 -0.575512 1.190496 -1.980201
35 1 0 0.364590 1.734272 -1.843180
36 1 0 -1.209556 1.784069 -2.647851
37 1 0 -1.083973 1.119151 -1.015074
38 1 0 0.449782 -3.201525 -2.750199
39 1 0 1.986609 -2.311869 -2.936298
40 1 0 1.708175 -3.335003 -1.501791
41 27 0 -0.474960 -1.847125 0.527150
42 7 0 2.800027 1.233764 0.855291
43 7 0 2.925774 0.026414 -1.688086
44 6 0 4.953047 1.223206 -2.155612
45 1 0 5.001083 0.726068 -3.118728
46 6 0 5.891080 2.143757 -1.766968
47 1 0 6.713390 2.391884 -2.429846
48 6 0 5.800617 2.785812 -0.504818
49 6 0 4.776290 2.495927 0.357836
50 1 0 4.691169 2.970794 1.329581
51 1 0 6.555973 3.512047 -0.223969
52 8 0 -1.151573 -3.432028 -0.395105
53 6 0 -2.384129 -3.380718 -0.680316
54 6 0 -3.032789 -4.473938 -1.457267
55 1 0 -4.088945 -4.271796 -1.638550
56 1 0 -2.511659 -4.586399 -2.411383
57 1 0 -2.931697 -5.412805 -0.907012
58 7 0 -3.077942 -2.331649 -0.273146
59 1 0 -4.068237 -2.254994 -0.472033
60 6 0 -2.398172 -1.373702 0.577271
61 6 0 -2.945556 -0.058412 0.495807
62 6 0 -3.359601 1.076559 0.384528
63 6 0 -2.107010 -1.945594 1.942237
64 1 0 -2.761002 -2.779696 2.206751
65 6 0 -3.735773 2.447926 0.233541
66 6 0 -4.307768 2.899654 -0.966122
67 6 0 -3.496748 3.364927 1.269497
68 6 0 -4.635080 4.241485 -1.120844
69 1 0 -4.486505 2.193038 -1.770365
70 6 0 -3.824602 4.705590 1.102764
71 1 0 -3.048455 3.019109 2.195727
72 6 0 -4.394474 5.148326 -0.089728

73 1 0 -5.076322 4.582185 -2.052242
74 1 0 -3.632407 5.408683 1.907165
75 1 0 -4.646603 6.196339 -0.216652
76 1 0 -2.091074 -1.197253 2.731867
77 1 0 -1.079083 -2.475947 2.009677
78 1 0 0.833979 -2.530397 0.788965

9

E(RwB97XD/6-311G++(d, p)) = -3471.483946 a. u.

ΔG(RwB97XD/6-31G(d, p)) = 0.567796 a. u.

Standard orientation:

Center Atomic Atomic Coordinates (Angstroms)
Number Number Type X Y Z
----
1 6 0 -2.581369 -0.240169 0.158382
2 6 0 -1.603855 -1.260490 -0.031604
3 6 0 -3.262262 -2.800919 -0.303874
4 6 0 -4.237063 -1.794965 -0.057865
5 15 0 -1.958149 1.482710 0.338735
6 15 0 0.166774 -0.787811 0.142277
7 6 0 -2.677924 2.034168 1.921260
8 1 0 -2.523408 3.110604 2.030945
9 1 0 -3.746223 1.812825 1.976525
10 1 0 -2.163254 1.524668 2.739416
11 6 0 0.977664 -1.821537 -1.114680
12 6 0 -2.837290 2.445013 -1.006674
13 6 0 -2.617208 1.706759 -2.334363
14 1 0 -1.557338 1.514629 -2.526541
15 1 0 -3.150733 0.751464 -2.358508
16 1 0 -3.001745 2.323460 -3.153999
17 6 0 -2.198786 3.840820 -1.064803
18 1 0 -2.711894 4.432518 -1.831116
19 1 0 -2.299034 4.367930 -0.110673
20 1 0 -1.137526 3.796069 -1.317944
21 6 0 -4.339281 2.593334 -0.732506
22 1 0 -4.529817 3.188409 0.165713
23 1 0 -4.799262 3.115321 -1.579632
24 1 0 -4.831627 1.624983 -0.618634
25 6 0 0.654410 -1.463331 1.820638
26 6 0 2.148558 -1.173458 2.021815
27 1 0 2.770742 -1.712752 1.302003
28 1 0 2.370087 -0.105493 1.934662
29 1 0 2.438604 -1.500956 3.026410
30 6 0 0.401987 -2.968953 1.940604
31 1 0 0.721750 -3.301311 2.934975

32 1 0 -0.655314 -3.219343 1.824841
33 1 0 0.974134 -3.536810 1.200703
34 6 0 -0.160591 -0.699206 2.874853
35 1 0 -1.237016 -0.861714 2.757524
36 1 0 0.123521 -1.047349 3.873827
37 1 0 0.033651 0.379344 2.829672
38 1 0 2.054453 -1.847752 -0.940999
39 1 0 0.573645 -2.835501 -1.077336
40 1 0 0.789283 -1.401623 -2.105617
41 8 0 0.444077 3.294157 0.725390
42 6 0 1.549373 3.829436 0.407925
43 7 0 2.435627 3.100675 -0.245783
44 6 0 2.039160 1.761616 -0.649035
45 6 0 0.935325 1.705804 -1.585545
46 1 0 0.883749 0.861900 -2.269473
47 1 0 3.306072 3.506991 -0.567183
48 6 0 1.825884 5.254771 0.741558
49 1 0 1.159998 5.887475 0.147595
50 1 0 1.601293 5.424633 1.796765
51 1 0 2.861142 5.528589 0.535469
52 1 0 0.618446 2.645188 -2.038896
53 6 0 3.125222 0.833173 -0.677051
54 6 0 4.034386 0.030659 -0.678740
55 6 0 5.067462 -0.958028 -0.637441
56 6 0 5.057220 -2.034643 -1.538446
57 6 0 6.087701 -0.875143 0.323137
58 6 0 6.047981 -3.007989 -1.472858
59 1 0 4.272167 -2.098761 -2.285334
60 6 0 7.076944 -1.849954 0.376198
61 1 0 6.094424 -0.047742 1.025506
62 6 0 7.059941 -2.918521 -0.518771
63 1 0 6.031823 -3.836587 -2.173987
64 1 0 7.861409 -1.777819 1.122985
65 1 0 7.832643 -3.679375 -0.473104
66 27 0 0.289435 1.436509 0.198317
67 7 0 -3.867340 -0.507101 0.169565
68 7 0 -1.935774 -2.507326 -0.274805
69 6 0 -3.678184 -4.130387 -0.564233
70 1 0 -2.919077 -4.883003 -0.749566
71 6 0 -5.014782 -4.432619 -0.568532
72 1 0 -5.339385 -5.449255 -0.764305
73 6 0 -5.987261 -3.430265 -0.317150
74 6 0 -5.612502 -2.136304 -0.065032
75 1 0 -6.340906 -1.355147 0.125755
76 1 0 -7.038956 -3.696943 -0.327127

E(RwB97XD/6-311G++(d, p)) = -3472.671073 a. u.

ΔG(RwB97XD/6-31G(d, p)) = 0.588811 a. u.

Standard orientation:

Center Atomic Atomic Coordinates (Angstroms)
Number Number Type X Y Z
----
1 6 0 -2.562417 -0.225256 0.142899
2 6 0 -1.576974 -1.238436 -0.026985
3 6 0 -3.223400 -2.792634 -0.304963
4 6 0 -4.206701 -1.793296 -0.066544
5 15 0 -1.961884 1.504405 0.293272
6 15 0 0.193504 -0.775836 0.194009
7 6 0 -2.775314 2.095768 1.815229
8 1 0 -2.605269 3.170064 1.923103
9 1 0 -3.849281 1.896712 1.803784
10 1 0 -2.323987 1.584416 2.669529
11 6 0 0.982969 -1.644327 -1.198274
12 6 0 -2.792125 2.403997 -1.126520
13 6 0 -2.486535 1.630227 -2.416193
14 1 0 -1.415441 1.450634 -2.543952
15 1 0 -3.003086 0.665428 -2.438506
16 1 0 -2.835596 2.212744 -3.275757
17 6 0 -2.195409 3.817552 -1.195413
18 1 0 -2.691634 4.370369 -2.001009
19 1 0 -2.356127 4.366996 -0.262429
20 1 0 -1.123502 3.803679 -1.399427
21 6 0 -4.310348 2.517462 -0.935827
22 1 0 -4.564695 3.140386 -0.073068
23 1 0 -4.738034 2.995329 -1.824829
24 1 0 -4.783432 1.540716 -0.811094
25 6 0 0.686860 -1.736304 1.737222
26 6 0 2.087930 -1.283996 2.173833
27 1 0 2.827192 -1.444426 1.383807
28 1 0 2.120633 -0.231321 2.466148
29 1 0 2.391559 -1.878256 3.042745
30 6 0 0.730359 -3.247052 1.462860
31 1 0 0.935186 -3.763607 2.407305
32 1 0 -0.215941 -3.623390 1.067240
33 1 0 1.529179 -3.507090 0.762522
34 6 0 -0.338362 -1.451356 2.843856
35 1 0 -1.318749 -1.875680 2.606372
36 1 0 0.007741 -1.917480 3.772492
37 1 0 -0.464993 -0.382503 3.039216
38 1 0 2.065727 -1.663742 -1.064982
39 1 0 0.601029 -2.665811 -1.252524
40 1 0 0.746223 -1.138837 -2.136205

41 8 0 0.325515 3.390286 0.740819
42 6 0 1.433990 3.934177 0.476712
43 7 0 2.385417 3.191545 -0.067639
44 6 0 2.025218 1.848427 -0.485471
45 6 0 0.946485 1.768572 -1.442599
46 1 0 0.941678 0.935396 -2.137070
47 1 0 3.270242 3.599545 -0.343437
48 6 0 1.661824 5.379378 0.759883
49 1 0 0.990755 5.970508 0.130585
50 1 0 1.412759 5.582517 1.803859
51 1 0 2.693024 5.674409 0.563328
52 1 0 0.612822 2.702044 -1.891152
53 6 0 3.124838 0.932940 -0.505655
54 6 0 4.030310 0.127878 -0.536075
55 6 0 5.044744 -0.879481 -0.606967
56 6 0 5.060462 -1.771995 -1.690664
57 6 0 6.008761 -1.006633 0.404549
58 6 0 6.023378 -2.771924 -1.757032
59 1 0 4.314317 -1.674137 -2.472844
60 6 0 6.973012 -2.005130 0.324678
61 1 0 5.995702 -0.319676 1.244702
62 6 0 6.982292 -2.889853 -0.752428
63 1 0 6.026147 -3.460841 -2.595865
64 1 0 7.718750 -2.094495 1.108328
65 1 0 7.735618 -3.669253 -0.808200
66 27 0 0.289694 1.465122 0.365529
67 7 0 -3.847027 -0.501182 0.147388
68 7 0 -1.900571 -2.487529 -0.271533
69 6 0 -3.627551 -4.127317 -0.557918
70 1 0 -2.862142 -4.874883 -0.737888
71 6 0 -4.961375 -4.440853 -0.558540
72 1 0 -5.277327 -5.461628 -0.746742
73 6 0 -5.942596 -3.445270 -0.312423
74 6 0 -5.579410 -2.146433 -0.069920
75 1 0 -6.314293 -1.370131 0.115774
76 1 0 -6.991787 -3.721714 -0.318985
77 1 0 0.939107 1.358765 1.875055
78 1 0 0.145540 1.336280 2.015632

13(α)

E(RwB97XD/6-311G++(d, p)) = -3472.636861 a. u.

ΔG(RwB97XD/6-31G(d, p)) = 0.580706 a. u.

Standard orientation:

Center Atomic Atomic Coordinates (Angstroms)
Number Number Type X Y Z

----
1 6 0 2.677000 0.057517 0.305839
2 6 0 1.602704 0.903975 -0.102361
3 6 0 3.084073 2.591701 -0.491607
4 6 0 4.142682 1.774968 -0.005750
5 15 0 2.263259 -1.709230 0.633629
6 15 0 -0.093701 0.233249 0.098387
7 6 0 3.086849 -2.084002 2.204323
8 1 0 3.056932 -3.162219 2.378612
9 1 0 4.123846 -1.740777 2.195114
10 1 0 2.548880 -1.580893 3.011150
11 6 0 -0.944070 0.695886 -1.441889
12 6 0 3.163038 -2.625343 -0.723516
13 6 0 2.712033 -2.013101 -2.057624
14 1 0 1.620240 -2.004542 -2.155277
15 1 0 3.076051 -0.988218 -2.181135
16 1 0 3.114954 -2.610162 -2.882720
17 6 0 2.744131 -4.100839 -0.655863
18 1 0 3.315247 -4.666719 -1.399971
19 1 0 2.948672 -4.536659 0.327422
20 1 0 1.680529 -4.231863 -0.871504
21 6 0 4.685899 -2.522805 -0.580364
22 1 0 5.039873 -3.023394 0.325888
23 1 0 5.152553 -3.019252 -1.438867
24 1 0 5.028312 -1.485413 -0.559136
25 6 0 -0.787269 1.278882 1.488939
26 6 0 -1.168549 2.679557 0.995919
27 1 0 -0.315428 3.197075 0.547282
28 1 0 -1.978007 2.643881 0.261045
29 1 0 -1.518104 3.273209 1.848179
30 6 0 0.273593 1.380162 2.594246
31 1 0 -0.175192 1.867051 3.466952
32 1 0 0.628885 0.393044 2.909777
33 1 0 1.136151 1.979421 2.288261
34 6 0 -2.024476 0.561086 2.044156
35 1 0 -1.751105 -0.379045 2.531377
36 1 0 -2.507542 1.206090 2.786731
37 1 0 -2.752360 0.334308 1.263042
38 1 0 -1.997873 0.419654 -1.356679
39 1 0 -0.859769 1.763315 -1.655920
40 1 0 -0.501269 0.128372 -2.264682
41 27 0 0.117120 -1.946959 0.536627
42 7 0 3.913278 0.491767 0.381955
43 7 0 1.803682 2.134305 -0.514681
44 6 0 3.361081 3.911547 -0.925791
45 1 0 2.539431 4.517543 -1.293152
46 6 0 4.643180 4.391328 -0.860617
47 1 0 4.860162 5.403458 -1.185744
48 6 0 5.697409 3.580807 -0.365462
49 6 0 5.458294 2.298509 0.054812

50 1 0 6.251466 1.660345 0.430343
51 1 0 6.702305 3.987493 -0.321783
52 8 0 -1.811774 -2.265762 0.322940
53 6 0 -2.203625 -2.956736 -0.645051
54 6 0 -1.241762 -3.664538 -1.548124
55 1 0 -1.730485 -4.143861 -2.396841
56 1 0 -0.508572 -2.943151 -1.926289
57 1 0 -0.709176 -4.419231 -0.961989
58 7 0 -3.508456 -3.103079 -0.894751
59 1 0 -3.783280 -3.716669 -1.651162
60 6 0 -4.552802 -2.448850 -0.165645
61 6 0 -4.607185 -1.023264 -0.288554
62 6 0 -4.638127 0.182787 -0.397737
63 6 0 -5.445272 -3.166824 0.519507
64 1 0 -5.354423 -4.246076 0.580896
65 6 0 -4.613366 1.614054 -0.449830
66 6 0 -4.199078 2.279403 -1.612655
67 6 0 -4.949578 2.356291 0.692466
68 6 0 -4.112670 3.666897 -1.624160
69 1 0 -3.937929 1.705277 -2.495680
70 6 0 -4.862840 3.743031 0.669688
71 1 0 -5.263894 1.838679 1.592691
72 6 0 -4.441119 4.399945 -0.484919
73 1 0 -3.785804 4.176695 -2.524659
74 1 0 -5.118334 4.311219 1.558358
75 1 0 -4.367031 5.482745 -0.497156
76 1 0 -6.279848 -2.685051 1.015676
77 1 0 0.096377 -1.646978 1.936026
78 1 0 0.362553 -3.338136 1.050918

TS4

E(RwB97XD/6-311G++(d, p)) = -3472.631417 a. u.

ΔG(RwB97XD/6-31G(d, p)) = 0.582995 a. u.

ν = *i*72.42

Standard orientation:

Center Number	Atomic Number	Atomic Type	Coordinates (Angstroms) X	Y	Z
1	6	0	-2.502279	0.423795	-0.631816
2	6	0	-1.625693	1.063455	0.286388

3	6	0	-3.233809	2.623897	0.714564
4	6	0	-4.048446	2.068589	-0.309993
5	15	0	-2.008771	-1.263079	-1.184579
6	15	0	0.088176	0.403136	0.478448
7	6	0	-2.378227	-1.224957	-2.962754
8	1	0	-2.288791	-2.226500	-3.388465
9	1	0	-3.383290	-0.834147	-3.133630
10	1	0	-1.652637	-0.567960	-3.447444
11	6	0	0.185261	0.379583	2.305193
12	6	0	-3.302206	-2.344722	-0.370348
13	6	0	-3.379172	-1.958113	1.113537
14	1	0	-2.397286	-1.999666	1.591653
15	1	0	-3.803880	-0.958741	1.254085
16	1	0	-4.035051	-2.669405	1.626856
17	6	0	-2.879264	-3.814862	-0.512406
18	1	0	-3.682203	-4.451048	-0.123531
19	1	0	-2.714162	-4.090709	-1.558966
20	1	0	-1.968724	-4.029696	0.051961
21	6	0	-4.676193	-2.154522	-1.030052
22	1	0	-4.683352	-2.521066	-2.061353
23	1	0	-5.411540	-2.737199	-0.464051
24	1	0	-4.997640	-1.110022	-1.030142
25	6	0	1.198988	1.824179	-0.121514
26	6	0	2.333880	2.009673	0.900070
27	1	0	1.958494	2.454262	1.826077
28	1	0	2.836526	1.074076	1.146899
29	1	0	3.077304	2.696260	0.481282
30	6	0	0.472786	3.169738	-0.269151
31	1	0	1.212091	3.908773	-0.598077
32	1	0	-0.313703	3.130523	-1.027211

33	1	0	0.038091	3.520863	0.668130
34	6	0	1.755442	1.452188	-1.503109
35	1	0	0.952945	1.381358	-2.243812
36	1	0	2.444651	2.239407	-1.830018
37	1	0	2.299451	0.506415	-1.499179
38	1	0	1.179783	0.072456	2.635239
39	1	0	-0.048210	1.361555	2.722854
40	1	0	-0.550544	-0.339847	2.672899
41	27	0	-0.005917	-1.592685	-0.611255
42	7	0	-3.669593	0.932042	-0.953285
43	7	0	-2.008154	2.106681	0.985423
44	6	0	-3.687418	3.756645	1.435390
45	1	0	-3.051981	4.159956	2.217047
46	6	0	-4.895046	4.320593	1.118253
47	1	0	-5.245390	5.193361	1.659302
48	6	0	-5.697746	3.780974	0.079420
49	6	0	-5.289739	2.676898	-0.623045
50	1	0	-5.894425	2.241987	-1.412199
51	1	0	-6.647938	4.250595	-0.152419
52	8	0	-0.195238	-2.657747	1.063246
53	6	0	0.757824	-2.870544	1.838541
54	6	0	0.516196	-3.381297	3.221561
55	1	0	1.442281	-3.518936	3.780226
56	1	0	-0.129978	-2.673320	3.747036
57	1	0	-0.013100	-4.334645	3.153963
58	7	0	2.023266	-2.626220	1.468384
59	1	0	2.760351	-2.715216	2.157600
60	6	0	2.392594	-2.244729	0.148428
61	6	0	3.419462	-1.259797	0.045933
62	6	0	4.365742	-0.517098	-0.087139

63	6	0	2.008944	-2.976782	-0.924017
64	1	0	1.475795	-3.911253	-0.795167
65	6	0	5.412514	0.439773	-0.264156
66	6	0	6.011336	1.046847	0.849304
67	6	0	5.816927	0.801250	-1.558226
68	6	0	7.005426	2.000393	0.665167
69	1	0	5.691561	0.768972	1.848255
70	6	0	6.810064	1.758559	-1.730236
71	1	0	5.346975	0.333332	-2.416878
72	6	0	7.406077	2.357658	-0.621458
73	1	0	7.467549	2.467588	1.528571
74	1	0	7.118919	2.037421	-2.732587
75	1	0	8.181884	3.104234	-0.759220
76	1	0	2.366495	-2.713623	-1.911540
77	1	0	0.217030	-0.818205	-1.833151
78	1	0	-0.687756	-2.649679	-1.478649

21

E(RwB97XD/6-311G++(d, p)) = -3472.683584 a. u.

ΔG(RwB97XD/6-31G(d, p)) = 0.588446 a. u.

Standard orientation:

Center Atomic Atomic Coordinates (Angstroms)
Number Number Type X Y Z
----
1 6 0 -2.476277 0.340460 -0.665338
2 6 0 -1.808870 1.039227 0.383415
3 6 0 -3.527490 2.537466 0.462630
4 6 0 -4.144803 1.894902 -0.645006
5 15 0 -1.765528 -1.267836 -1.178988
6 15 0 -0.175158 0.396420 0.959898
7 6 0 -1.826904 -1.207395 -2.997166
8 1 0 -1.567328 -2.187536 -3.405287
9 1 0 -2.819467 -0.917339 -3.348944
10 1 0 -1.092994 -0.476634 -3.344158

11 6 0 -0.531811 -0.005572 2.701019
12 6 0 -3.013845 -2.544122 -0.631179
13 6 0 -3.283393 -2.330053 0.864940
14 1 0 -2.359024 -2.383970 1.446611
15 1 0 -3.771893 -1.369216 1.057796
16 1 0 -3.953636 -3.119761 1.222312
17 6 0 -2.357365 -3.916084 -0.853314
18 1 0 -3.062186 -4.701174 -0.557789
19 1 0 -2.103754 -4.078041 -1.906518
20 1 0 -1.451074 -4.028858 -0.251443
21 6 0 -4.321416 -2.458885 -1.426662
22 1 0 -4.164228 -2.674690 -2.487716
23 1 0 -5.023260 -3.204646 -1.035916
24 1 0 -4.788475 -1.474056 -1.339960
25 6 0 1.009578 1.845103 0.994345
26 6 0 2.195082 1.415045 1.876806
27 1 0 1.923949 1.435780 2.936751
28 1 0 2.568191 0.418310 1.634644
29 1 0 3.017299 2.123896 1.729723
30 6 0 0.405659 3.122581 1.596663
31 1 0 1.219050 3.846288 1.724527
32 1 0 -0.345776 3.569262 0.943897
33 1 0 -0.048523 2.953710 2.576625
34 6 0 1.462464 2.144513 -0.439786
35 1 0 0.608543 2.381184 -1.084277
36 1 0 2.126814 3.015719 -0.427030
37 1 0 2.007528 1.308803 -0.881477
38 1 0 0.356118 -0.429644 3.174696
39 1 0 -0.843678 0.889163 3.245258
40 1 0 -1.333655 -0.745266 2.732417
41 27 0 0.270257 -1.342727 -0.286713
42 7 0 -3.604832 0.769293 -1.182221
43 7 0 -2.346712 2.087389 0.960507
44 6 0 -4.140836 3.674802 1.044370
45 1 0 -3.656550 4.146412 1.892967
46 6 0 -5.312259 4.154544 0.519444
47 1 0 -5.784066 5.028433 0.956301
48 6 0 -5.919465 3.523386 -0.597346
49 6 0 -5.353351 2.414905 -1.171318
50 1 0 -5.806980 1.914638 -2.020452
51 1 0 -6.844399 3.926975 -0.995886
52 8 0 0.003455 -2.627000 1.211001
53 6 0 1.089156 -2.943797 1.770614
54 6 0 1.099282 -3.799737 2.990997
55 1 0 2.091981 -3.867698 3.437505
56 1 0 0.392497 -3.389889 3.715963
57 1 0 0.758913 -4.803138 2.719721
58 7 0 2.223245 -2.505516 1.238811
59 1 0 3.118979 -2.767153 1.632763
60 6 0 2.127726 -1.805303 -0.029855

61 6 0 3.269601 -0.981183 -0.297413
62 6 0 4.213810 -0.251426 -0.508478
63 6 0 1.672360 -2.694505 -1.168159
64 1 0 1.784612 -3.757653 -0.947631
65 6 0 5.280139 0.678689 -0.723098
66 6 0 5.904645 1.295241 0.372498
67 6 0 5.694223 1.002777 -2.023792
68 6 0 6.922117 2.219511 0.165512
69 1 0 5.585118 1.046839 1.379644
70 6 0 6.710858 1.930601 -2.220022
71 1 0 5.213101 0.528057 -2.872873
72 6 0 7.327272 2.540617 -1.129006
73 1 0 7.398316 2.692072 1.018808
74 1 0 7.022176 2.177607 -3.230151
75 1 0 8.119492 3.265676 -1.286515
76 1 0 2.122661 -2.434471 -2.123178
77 1 0 0.536533 -0.356144 -1.330687
78 1 0 0.537573 -2.636987 -1.379027

TS10

E(RwB97XD/6-311G++(d, p)) = -3472.639817 a. u.

ΔG(RwB97XD/6-31G(d, p)) = 0.582235 a. u.

ν = *i*933.34

Standard orientation:

Center Atomic Atomic Coordinates (Angstroms)
Number Number Type X Y Z
----
1 6 0 -2.307908 0.381705 -0.724687
2 6 0 -2.153068 0.573099 0.677852
3 6 0 -3.847940 2.094786 0.654327
4 6 0 -3.967927 1.941024 -0.754354
5 15 0 -1.262175 -0.928893 -1.471743
6 15 0 -0.787402 -0.367953 1.478861
7 6 0 -0.680817 -0.158059 -3.021450
8 1 0 -0.203349 -0.911108 -3.653808
9 1 0 -1.502212 0.307304 -3.571026
10 1 0 0.060362 0.604107 -2.767856
11 6 0 -1.641788 -1.385280 2.724921
12 6 0 -2.494171 -2.243030 -1.990823
13 6 0 -3.305527 -2.634013 -0.748859
14 1 0 -2.653977 -2.944796 0.074941
15 1 0 -3.939837 -1.813027 -0.399011
16 1 0 -3.958912 -3.478776 -0.994071
17 6 0 -1.683927 -3.453698 -2.476796

18 1 0 -2.370841 -4.222074 -2.848870
19 1 0 -1.004169 -3.191338 -3.294856
20 1 0 -1.095720 -3.896620 -1.666181
21 6 0 -3.433862 -1.771220 -3.106108
22 1 0 -2.893362 -1.572774 -4.036461
23 1 0 -4.166805 -2.559893 -3.311838
24 1 0 -3.979202 -0.866274 -2.824941
25 6 0 0.182952 0.918238 2.436090
26 6 0 1.459489 0.246367 2.964353
27 1 0 1.242834 -0.670914 3.523404
28 1 0 2.156917 0.004035 2.160118
29 1 0 1.966118 0.939618 3.644515
30 6 0 -0.617569 1.465570 3.626554
31 1 0 -0.032406 2.259790 4.104273
32 1 0 -1.577345 1.884450 3.317559
33 1 0 -0.798342 0.692878 4.379755
34 6 0 0.555295 2.059781 1.480720
35 1 0 -0.323303 2.633255 1.169456
36 1 0 1.237703 2.746153 1.993864
37 1 0 1.063955 1.692455 0.582651
38 1 0 -0.902373 -1.844671 3.384412
39 1 0 -2.331977 -0.778081 3.314886
40 1 0 -2.199327 -2.175587 2.218425
41 27 0 0.278736 -1.478065 -0.053391
42 7 0 -3.182757 1.059608 -1.430589
43 7 0 -2.918372 1.394180 1.357144
44 6 0 -4.696799 3.002755 1.334587
45 1 0 -4.586576 3.107659 2.408849
46 6 0 -5.620839 3.726364 0.626959
47 1 0 -6.272256 4.424497 1.142176
48 6 0 -5.738724 3.575411 -0.778777
49 6 0 -4.931108 2.703168 -1.461079
50 1 0 -5.009724 2.569545 -2.534883
51 1 0 -6.479605 4.159535 -1.314661
52 8 0 0.647396 -2.988747 1.277845
53 6 0 1.857217 -3.021833 1.607035
54 6 0 2.355710 -3.952715 2.663869
55 1 0 1.637251 -3.971127 3.485342
56 1 0 2.420232 -4.960965 2.244145
57 1 0 3.339563 -3.662985 3.036415
58 7 0 2.721614 -2.208184 0.996413
59 1 0 3.696638 -2.199009 1.269778
60 6 0 2.285949 -1.394697 -0.126903
61 6 0 3.292758 -0.391785 -0.418383
62 6 0 4.134292 0.454422 -0.618168
63 6 0 1.863378 -2.246853 -1.323540
64 1 0 2.381471 -3.206826 -1.322333
65 6 0 5.103553 1.486837 -0.845906
66 6 0 4.782158 2.824648 -0.571661
67 6 0 6.376182 1.169971 -1.342729

68 6 0 5.722167 3.825130 -0.789758
69 1 0 3.797199 3.069342 -0.186483
70 6 0 7.310335 2.176902 -1.557171
71 1 0 6.623610 0.135315 -1.557147
72 6 0 6.986327 3.503940 -1.281548
73 1 0 5.468279 4.858183 -0.574620
74 1 0 8.293544 1.924769 -1.941644
75 1 0 7.718288 4.287673 -1.449510
76 1 0 1.989251 -1.731314 -2.273646
77 1 0 1.237764 -0.363838 -0.130854
78 1 0 0.771125 -2.588951 -1.300422

14(β)

E(RwB97XD/6-311G++(d, p)) = -3472.636861 a. u.

ΔG(RwB97XD/6-31G(d, p)) = 0.580706 a. u.

Standard orientation:

Center Atomic Atomic Coordinates (Angstroms)
Number Number Type X Y Z
----
1 6 0 -2.842789 -0.911887 -0.282808
2 6 0 -3.611523 0.196577 0.181087
3 6 0 -5.531691 -0.962700 -0.219854
4 6 0 -4.769916 -2.034206 -0.762682
5 15 0 -1.013891 -0.800889 -0.097381
6 15 0 -2.682439 1.715243 0.638245
7 6 0 -0.404221 -1.148865 -1.778634
8 1 0 0.683959 -1.252602 -1.756492
9 1 0 -0.846278 -2.060535 -2.187305
10 1 0 -0.663999 -0.307056 -2.426109
11 6 0 -3.506555 2.297410 2.143716
12 6 0 -0.574574 -2.273347 0.969089
13 6 0 -1.495553 -2.264315 2.197052
14 1 0 -1.459393 -1.305982 2.725277
15 1 0 -2.536173 -2.475316 1.932438
16 1 0 -1.166109 -3.043158 2.893341
17 6 0 0.882929 -2.094430 1.417711
18 1 0 1.195065 -2.984603 1.975446
19 1 0 1.562726 -1.977338 0.567318
20 1 0 0.996326 -1.224408 2.067329
21 6 0 -0.717651 -3.599237 0.212109
22 1 0 -0.000472 -3.671594 -0.611274
23 1 0 -0.510301 -4.422432 0.905462
24 1 0 -1.724344 -3.735914 -0.190209
25 6 0 -3.055599 2.920117 -0.738975
26 6 0 -2.293290 4.220422 -0.442713
27 1 0 -2.590660 4.658375 0.515093

28 1 0 -1.211220 4.062064 -0.427572
29 1 0 -2.518926 4.949655 -1.228434
30 6 0 -4.553691 3.219407 -0.857051
31 1 0 -4.707290 3.919063 -1.686446
32 1 0 -5.139024 2.319405 -1.060537
33 1 0 -4.945620 3.688743 0.050284
34 6 0 -2.531656 2.298671 -2.042223
35 1 0 -3.048078 1.365893 -2.291270
36 1 0 -2.696941 3.000967 -2.866403
37 1 0 -1.455219 2.098464 -1.990334
38 1 0 -3.101994 3.272752 2.422926
39 1 0 -4.586278 2.372745 1.998296
40 1 0 -3.300460 1.586695 2.947549
41 8 0 1.345289 0.993491 0.878071
42 6 0 2.085384 1.410991 -0.037101
43 7 0 3.410043 1.198681 -0.015965
44 6 0 4.165805 0.480944 0.944658
45 6 0 3.733080 0.092631 2.152894
46 1 0 3.932315 1.555775 -0.805793
47 6 0 1.520038 2.166443 -1.200726
48 1 0 0.777988 1.533392 -1.698154
49 1 0 1.012142 3.057862 -0.817611
50 1 0 2.273438 2.467234 -1.928615
51 27 0 -0.592059 1.238317 0.753599
52 7 0 -3.411162 -1.989388 -0.773291
53 7 0 -4.922700 0.164078 0.235960
54 6 0 -6.944755 -1.060675 -0.175386
55 1 0 -7.506495 -0.234427 0.247607
56 6 0 -7.561636 -2.180528 -0.668704
57 1 0 -8.643156 -2.261985 -0.638127
58 6 0 -6.802475 -3.242986 -1.224358
59 6 0 -5.434544 -3.176457 -1.273879
60 1 0 -4.835509 -3.981556 -1.686436
61 1 0 -7.316092 -4.117144 -1.610536
62 1 0 -0.877062 0.734240 2.065061
63 1 0 2.739107 0.314997 2.509987
64 1 0 4.409079 -0.463489 2.791078
65 6 0 5.495830 0.188199 0.499168
66 6 0 6.620747 -0.053757 0.122803
67 6 0 7.957525 -0.360168 -0.288069
68 6 0 8.766124 -1.174507 0.519347
69 6 0 8.466905 0.149948 -1.491364
70 6 0 10.065296 -1.470584 0.125126
71 1 0 8.369000 -1.569538 1.448688
72 6 0 9.768100 -0.152225 -1.875652
73 1 0 7.840847 0.779652 -2.115051
74 6 0 10.568633 -0.960697 -1.070489
75 1 0 10.686767 -2.100482 0.753430
76 1 0 10.159420 0.246626 -2.806041
77 1 0 11.584921 -1.192022 -1.373420

78 1 0 -0.486700 2.564444 1.449362

TS5

E(RwB97XD/6-311G++(d, p)) = -3472.628002 a. u.

ΔG(RwB97XD/6-31G(d, p)) = 0.582277 a. u.

$\nu = i37.98$

Standard orientation:

Center Atomic Atomic Coordinates (Angstroms)
Number Number Type X Y Z
----
1 6 0 -2.939815 0.597615 0.175982
2 6 0 -3.020258 -0.616639 -0.568508
3 6 0 -5.263690 -0.703723 -0.182330
4 6 0 -5.170232 0.465115 0.622473
5 15 0 -1.321577 1.464978 0.198646
6 15 0 -1.435376 -1.371592 -1.130086
7 6 0 -1.027999 1.809937 1.961799
8 1 0 -1.925718 2.204046 2.444876
9 1 0 -0.731276 0.886913 2.462029
10 1 0 -0.216793 2.536259 2.052961
11 6 0 -1.742946 -1.850865 -2.850148
12 6 0 -1.664422 3.107882 -0.624113
13 6 0 -2.399247 2.860768 -1.946707
14 1 0 -1.824478 2.220000 -2.621855
15 1 0 -3.384102 2.411363 -1.795865
16 1 0 -2.549041 3.825471 -2.443518
17 6 0 -0.297884 3.748840 -0.898229
18 1 0 -0.444439 4.727381 -1.370246
19 1 0 0.263792 3.905905 0.030356
20 1 0 0.305118 3.130732 -1.570315
21 6 0 -2.499038 4.031501 0.270470
22 1 0 -1.987825 4.257038 1.211005
23 1 0 -2.661996 4.981694 -0.252673
24 1 0 -3.474408 3.596030 0.499267
25 6 0 -1.332384 -2.944783 -0.121199
26 6 0 0.080475 -3.528104 -0.280420
27 1 0 0.315179 -3.745718 -1.327478
28 1 0 0.843025 -2.856569 0.120340
29 1 0 0.128509 -4.468155 0.280767
30 6 0 -2.356878 -3.980607 -0.606086
31 1 0 -2.316016 -4.851319 0.058721
32 1 0 -3.376075 -3.587064 -0.598364

33 1 0 -2.128618 -4.329118 -1.617669
34 6 0 -1.606674 -2.593147 1.349084
35 1 0 -2.673580 -2.430461 1.529512
36 1 0 -1.285417 -3.428161 1.981409
37 1 0 -1.058631 -1.703607 1.667193
38 1 0 -0.873835 -2.394431 -3.229554
39 1 0 -2.631066 -2.481517 -2.923252
40 1 0 -1.891261 -0.951912 -3.452413
41 8 0 0.889994 -0.584809 0.866404
42 6 0 1.692482 -0.468539 1.797778
43 7 0 2.828162 0.255768 1.735024
44 6 0 3.380702 0.886308 0.587677
45 6 0 2.691071 1.745183 -0.170698
46 1 0 3.443777 0.184290 2.536106
47 6 0 1.416811 -1.168452 3.093899
48 1 0 0.583834 -0.664750 3.594567
49 1 0 1.105711 -2.192519 2.875812
50 1 0 2.277087 -1.180389 3.763891
51 27 0 0.100210 0.058231 -0.820841
52 7 0 -3.984305 1.110872 0.780357
53 7 0 -4.159432 -1.238861 -0.765498
54 6 0 -6.520169 -1.334800 -0.358068
55 1 0 -6.568714 -2.229805 -0.969413
56 6 0 -7.631622 -0.809004 0.246641
57 1 0 -8.596890 -1.285774 0.112628
58 6 0 -7.539673 0.360153 1.046245
59 6 0 -6.335419 0.985933 1.237699
60 1 0 -6.244209 1.883954 1.839735
61 1 0 -8.437759 0.760637 1.505158
62 1 0 -0.456365 0.593103 -2.057784
63 1 0 1.690807 2.055287 0.113916
64 1 0 3.128379 2.163615 -1.068977
65 6 0 4.745483 0.546191 0.327875
66 6 0 5.916860 0.292387 0.154052
67 6 0 7.307822 0.030561 -0.055849
68 6 0 7.999332 0.697572 -1.078594
69 6 0 7.992435 -0.870965 0.773979
70 6 0 9.357500 0.468044 -1.259780
71 1 0 7.466767 1.390391 -1.722340
72 6 0 9.351139 -1.090979 0.585260
73 1 0 7.455228 -1.387756 1.562768
74 6 0 10.036156 -0.420703 -0.425876
75 1 0 9.889458 0.989452 -2.049111
76 1 0 9.878455 -1.784661 1.232911
77 1 0 11.099268 -0.588863 -0.564303
78 1 0 0.882302 -0.866117 -1.712730

18

E(RwB97XD/6-311G++(d, p)) = -3472.653705 a. u.

∆G(RwB97XD/6-31G(d, p)) = 0.584770 a. u.

Standard orientation:

Center Atomic Atomic Coordinates (Angstroms)
Number Number Type X Y Z
----
1 6 0 -2.587720 0.646704 0.047120
2 6 0 -2.319973 -0.543233 -0.688695
3 6 0 -4.541048 -0.758307 -1.150781
4 6 0 -4.817130 0.389715 -0.357382
5 15 0 -1.135531 1.598776 0.663317
6 15 0 -0.577405 -1.142548 -0.698352
7 6 0 -1.664507 2.125029 2.322756
8 1 0 -1.007928 2.919944 2.684089
9 1 0 -2.695350 2.484969 2.305215
10 1 0 -1.588741 1.269721 2.997899
11 6 0 -0.114666 -1.111924 -2.455605
12 6 0 -1.144280 3.122436 -0.428651
13 6 0 -1.192113 2.670002 -1.893219
14 1 0 -0.351588 2.012848 -2.135054
15 1 0 -2.125256 2.152844 -2.137250
16 1 0 -1.122884 3.552332 -2.538518
17 6 0 0.144488 3.917361 -0.184531
18 1 0 0.074319 4.870557 -0.719829
19 1 0 0.298406 4.142172 0.875904
20 1 0 1.020404 3.384613 -0.563540
21 6 0 -2.354652 4.011020 -0.109970
22 1 0 -2.288594 4.437467 0.895234
23 1 0 -2.371781 4.842745 -0.822984
24 1 0 -3.300920 3.471226 -0.196338
25 6 0 -0.688106 -2.949977 -0.202917
26 6 0 0.721788 -3.434776 0.163676
27 1 0 1.437861 -3.261183 -0.646682
28 1 0 1.100848 -2.952451 1.067815
29 1 0 0.683059 -4.513718 0.348895
30 6 0 -1.219906 -3.805514 -1.363483
31 1 0 -1.350704 -4.831228 -1.000742
32 1 0 -2.185682 -3.446649 -1.726874
33 1 0 -0.516563 -3.836687 -2.200459
34 6 0 -1.620892 -3.084773 1.009211
35 1 0 -2.662211 -2.881017 0.744299
36 1 0 -1.567901 -4.114985 1.378454
37 1 0 -1.327603 -2.415475 1.820829
38 1 0 0.858975 -1.593131 -2.577784
39 1 0 -0.860639 -1.626718 -3.064401
40 1 0 -0.037263 -0.074029 -2.783049
41 8 0 0.098317 -0.735036 2.295308
42 6 0 1.108555 -1.034281 2.980097

43 7 0 2.304586 -0.610891 2.578775
44 6 0 2.371899 0.323810 1.478628
45 6 0 1.666565 1.536638 1.624652
46 1 0 3.141049 -0.820382 3.109528
47 6 0 0.985976 -1.854738 4.218441
48 1 0 0.366819 -1.311517 4.937197
49 1 0 0.475474 -2.789059 3.972385
50 1 0 1.956423 -2.070601 4.665777
51 27 0 0.585895 0.199864 0.578976
52 7 0 -3.810136 1.091555 0.225932
53 7 0 -3.269787 -1.215330 -1.295831
54 6 0 -5.607191 -1.447758 -1.780458
55 1 0 -5.374066 -2.320014 -2.382353
56 6 0 -6.892982 -1.007038 -1.607797
57 1 0 -7.714793 -1.530341 -2.085220
58 6 0 -7.169845 0.131234 -0.806390
59 6 0 -6.157272 0.818935 -0.190196
60 1 0 -6.347907 1.694965 0.420909
61 1 0 -8.197856 0.456247 -0.684985
62 1 0 1.012967 0.842921 -0.649661
63 1 0 1.211111 1.753406 2.586516
64 1 0 1.995090 2.392193 1.051467
65 6 0 3.584134 0.251374 0.708723
66 6 0 4.613638 0.206205 0.074699
67 6 0 5.830443 0.159720 -0.680236
68 6 0 6.374120 1.337959 -1.213376
69 6 0 6.483128 -1.063469 -0.893518
70 6 0 7.551922 1.288177 -1.949708
71 1 0 5.868987 2.283969 -1.047452
72 6 0 7.663126 -1.101723 -1.627096
73 1 0 6.060835 -1.974197 -0.481491
74 6 0 8.198655 0.070978 -2.156690
75 1 0 7.967370 2.202231 -2.361729
76 1 0 8.164851 -2.050671 -1.787191
77 1 0 9.120340 0.036512 -2.728938
78 1 0 1.459814 -0.865362 0.104876

TS8

E(RwB97XD/6-311G++(d, p)) = -3472.653705 a. u.

ΔG(RwB97XD/6-31G(d, p)) = 0.58477 a. u.

$\nu = i369.69$

Standard orientation:

Center Atomic Atomic Coordinates (Angstroms)

Number	Number	Type	X	Y	Z
1	6	0	-2.623171	0.643503	0.015572
2	6	0	-2.338878	-0.575448	-0.668517
3	6	0	-4.561267	-0.849005	-1.100237
4	6	0	-4.850653	0.333994	-0.365827
5	15	0	-1.188712	1.636910	0.609737
6	15	0	-0.585569	-1.143757	-0.682133
7	6	0	-1.714251	2.172271	2.267024
8	1	0	-1.036847	2.947963	2.632059
9	1	0	-2.735929	2.557269	2.250511
10	1	0	-1.659010	1.313213	2.939309
11	6	0	-0.148936	-1.135753	-2.447915
12	6	0	-1.209853	3.146545	-0.494832
13	6	0	-1.255804	2.680372	-1.954953
14	1	0	-0.413435	2.023583	-2.191749
15	1	0	-2.187433	2.157061	-2.191956
16	1	0	-1.190961	3.556198	-2.609573
17	6	0	0.078542	3.942637	-0.251121
18	1	0	0.011751	4.894350	-0.789533
19	1	0	0.230421	4.168780	0.809381
20	1	0	0.954574	3.405278	-0.622730
21	6	0	-2.424136	4.032417	-0.184267
22	1	0	-2.359057	4.469554	0.816379
23	1	0	-2.447294	4.856592	-0.905794
24	1	0	-3.367215	3.485502	-0.263369
25	6	0	-0.640549	-2.943189	-0.157907
26	6	0	0.795351	-3.366280	0.186828
27	1	0	1.487771	-3.174594	-0.639944
28	1	0	1.173343	-2.855395	1.076193
29	1	0	0.807394	-4.442872	0.388883

30	6	0	-1.169220	-3.842618	-1.285210
31	1	0	-1.264505	-4.862844	-0.896406
32	1	0	-2.151412	-3.518428	-1.637246
33	1	0	-0.482049	-3.874538	-2.135698
34	6	0	-1.539439	-3.077226	1.079251
35	1	0	-2.593226	-2.918781	0.832631
36	1	0	-1.440262	-4.092646	1.479040
37	1	0	-1.253948	-2.372493	1.863584
38	1	0	0.840569	-1.582999	-2.573970
39	1	0	-0.881179	-1.689654	-3.039049
40	1	0	-0.112838	-0.102467	-2.797131
41	8	0	0.081925	-0.603132	2.319375
42	6	0	1.099630	-0.933886	2.977427
43	7	0	2.301578	-0.585027	2.522202
44	6	0	2.371167	0.304475	1.380165
45	6	0	1.705306	1.561034	1.527081
46	1	0	3.147425	-0.821873	3.025262
47	6	0	0.980750	-1.721277	4.237376
48	1	0	0.357334	-1.163452	4.940520
49	1	0	0.474786	-2.664080	4.013426
50	1	0	1.951045	-1.923850	4.691114
51	27	0	0.544081	0.279759	0.564389
52	7	0	-3.851659	1.077854	0.177958
53	7	0	-3.283405	-1.291569	-1.232635
54	6	0	-5.620006	-1.585829	-1.687034
55	1	0	-5.377322	-2.484271	-2.245047
56	6	0	-6.911550	-1.155856	-1.530066
57	1	0	-7.727511	-1.715110	-1.975672
58	6	0	-7.201498	0.018160	-0.787148
59	6	0	-6.196176	0.751825	-0.213609
60	1	0	-6.396361	1.655792	0.352158

61	1	0	-8.233697	0.333621	-0.676559
62	1	0	0.950651	0.886209	-0.690433
63	1	0	1.316323	1.810451	2.510782
64	1	0	2.077107	2.395975	0.947964
65	6	0	3.617447	0.227400	0.656223
66	6	0	4.663061	0.170855	0.051578
67	6	0	5.902881	0.118517	-0.665085
68	6	0	6.465514	1.294107	-1.183660
69	6	0	6.555521	-1.107848	-0.857729
70	6	0	7.663737	1.238452	-1.885693
71	1	0	5.959319	2.242082	-1.033239
72	6	0	7.755877	-1.151961	-1.557036
73	1	0	6.117728	-2.016124	-0.456730
74	6	0	8.310517	0.018065	-2.072691
75	1	0	8.094343	2.149989	-2.287463
76	1	0	8.258014	-2.103026	-1.702247
77	1	0	9.247590	-0.021192	-2.618991
78	1	0	1.597527	-0.694043	0.263146

22

E(RwB97XD/6-311G++(d, p)) = -3472.660724 a. u.

ΔG(RwB97XD/6-31G(d, p)) = 0.588200 a. u.

Standard orientation:

Center	Atomic	Atomic	Coordinates (Angstroms)		
Number	Number	Type	X	Y	Z
1	6	0	-2.702448	0.693842	0.036387
2	6	0	-2.528795	-0.568079	-0.608706

3	6	0	-4.793406	-0.768106	-0.812154
4	6	0	-4.966406	0.472308	-0.139295
5	15	0	-1.188065	1.640904	0.504635
6	15	0	-0.805248	-1.192579	-0.797968
7	6	0	-1.547846	2.178164	2.203910
8	1	0	-0.805688	2.916960	2.515440
9	1	0	-2.548678	2.608712	2.276867
10	1	0	-1.477086	1.308950	2.861254
11	6	0	-0.573334	-1.181604	-2.605586
12	6	0	-1.223185	3.161216	-0.579898
13	6	0	-1.385085	2.722641	-2.040318
14	1	0	-0.572916	2.060455	-2.353846
15	1	0	-2.339876	2.216126	-2.213335
16	1	0	-1.359036	3.610319	-2.681657
17	6	0	0.107957	3.902767	-0.401665
18	1	0	0.053786	4.857412	-0.936298
19	1	0	0.316743	4.119999	0.651127
20	1	0	0.943490	3.330802	-0.811723
21	6	0	-2.381293	4.086933	-0.180859
22	1	0	-2.243921	4.498581	0.823231
23	1	0	-2.410151	4.927400	-0.883013
24	1	0	-3.347867	3.578544	-0.219136
25	6	0	-0.832311	-2.992960	-0.289761
26	6	0	0.639124	-3.404331	-0.118186
27	1	0	1.218753	-3.224995	-1.030641
28	1	0	1.121345	-2.870006	0.705763
29	1	0	0.690118	-4.476327	0.101973
30	6	0	-1.488450	-3.892874	-1.344782
31	1	0	-1.522572	-4.917917	-0.957831
32	1	0	-2.511137	-3.577194	-1.565017

33	1	0	-0.917914	-3.910749	-2.278058
34	6	0	-1.578650	-3.123365	1.044995
35	1	0	-2.653286	-2.962830	0.917670
36	1	0	-1.436388	-4.137008	1.436410
37	1	0	-1.207400	-2.413938	1.788949
38	1	0	0.391192	-1.637777	-2.843924
39	1	0	-1.372425	-1.722685	-3.117145
40	1	0	-0.563157	-0.146161	-2.951385
41	8	0	0.153202	-0.597780	2.147544
42	6	0	1.176625	-0.995527	2.757474
43	7	0	2.380190	-0.780929	2.228506
44	6	0	2.466866	0.011267	1.003264
45	6	0	1.814683	1.361847	1.144915
46	1	0	3.224355	-1.083061	2.696458
47	6	0	1.058159	-1.735001	4.047528
48	1	0	0.423854	-1.159553	4.725152
49	1	0	0.565091	-2.692013	3.854054
50	1	0	2.027729	-1.913732	4.513053
51	27	0	0.455670	0.268056	0.351465
52	7	0	-3.892389	1.193800	0.276275
53	7	0	-3.551594	-1.273152	-1.034449
54	6	0	-5.930600	-1.494787	-1.244664
55	1	0	-5.776574	-2.440935	-1.753030
56	6	0	-7.183770	-0.991787	-1.010984
57	1	0	-8.059102	-1.542203	-1.339725
58	6	0	-7.356246	0.246970	-0.340257
59	6	0	-6.273652	0.968046	0.091249
60	1	0	-6.384045	1.917936	0.603846
61	1	0	-8.359856	0.623006	-0.171277
62	1	0	0.732492	0.875601	-0.943068

63	1	0	1.688802	1.663894	2.184966
64	1	0	2.278513	2.140767	0.546855
65	6	0	3.809473	0.009130	0.444591
66	6	0	4.933907	0.010646	0.002370
67	6	0	6.267753	0.061467	-0.521629
68	6	0	6.986857	1.265242	-0.485986
69	6	0	6.859057	-1.084659	-1.071816
70	6	0	8.278067	1.318311	-0.997196
71	1	0	6.527487	2.150334	-0.058119
72	6	0	8.151697	-1.021865	-1.579513
73	1	0	6.302384	-2.015759	-1.098193
74	6	0	8.861624	0.176990	-1.544465
75	1	0	8.828981	2.252869	-0.968556
76	1	0	8.605241	-1.910878	-2.005656
77	1	0	9.869436	0.222358	-1.944986
78	1	0	1.878594	-0.640525	0.206516

TS11

E(RwB97XD/6-311G++(d, p)) = -3472.631629 a. u.

ΔG(RwB97XD/6-31G(d, p)) = 0.580023 a. u.

ν = *i*-859.83

Standard orientation:

Center Number	Atomic Number	Atomic Type	Coordinates (Angstroms) X	Y	Z
1	6	0	-2.806919	0.443630	-0.190512
2	6	0	-2.366167	-0.894189	-0.410651

3	6	0	-4.531881	-1.550919	-0.702794
4	6	0	-4.972609	-0.228150	-0.425505
5	15	0	-1.504564	1.723532	0.056755
6	15	0	-0.558816	-1.203609	-0.226375
7	6	0	-2.111485	2.620986	1.519952
8	1	0	-1.498930	3.511056	1.683867
9	1	0	-3.157372	2.910648	1.397526
10	1	0	-2.012549	1.965995	2.387822
11	6	0	-0.116718	-1.886078	-1.861777
12	6	0	-1.681727	2.867745	-1.410075
13	6	0	-1.513158	2.027235	-2.682739
14	1	0	-0.553338	1.500193	-2.693411
15	1	0	-2.314654	1.289368	-2.793373
16	1	0	-1.544578	2.687807	-3.556135
17	6	0	-0.571014	3.925047	-1.327065
18	1	0	-0.731596	4.669899	-2.114267
19	1	0	-0.575718	4.450740	-0.366548
20	1	0	0.419297	3.491038	-1.480497
21	6	0	-3.044165	3.574579	-1.420741
22	1	0	-3.155568	4.248710	-0.566195
23	1	0	-3.116404	4.179106	-2.331832
24	1	0	-3.875956	2.866701	-1.413167
25	6	0	-0.444555	-2.634430	0.979532
26	6	0	1.036224	-2.776717	1.368652
27	1	0	1.684792	-2.878741	0.491193
28	1	0	1.385074	-1.925392	1.961540
29	1	0	1.162084	-3.676636	1.980695
30	6	0	-0.937423	-3.960363	0.386510
31	1	0	-0.906820	-4.730319	1.166130
32	1	0	-1.963162	-3.884805	0.018561

33	1	0	-0.297616	-4.296963	-0.435175
34	6	0	-1.267164	-2.263033	2.221042
35	1	0	-2.339552	-2.234108	2.004274
36	1	0	-1.106379	-3.017441	2.999681
37	1	0	-0.965480	-1.291104	2.625174
38	1	0	0.913476	-2.251783	-1.834910
39	1	0	-0.784545	-2.697944	-2.159656
40	1	0	-0.178634	-1.079063	-2.596234
41	8	0	0.314809	0.756067	2.465441
42	6	0	1.431201	0.838120	3.016555
43	7	0	2.549956	0.811412	2.278641
44	6	0	2.465841	0.766414	0.826140
45	6	0	1.833941	2.005788	0.203456
46	1	0	3.460189	0.886358	2.712372
47	6	0	1.560378	0.955442	4.502140
48	1	0	1.043260	1.861657	4.827381
49	1	0	1.062191	0.099700	4.964224
50	1	0	2.599812	0.993407	4.829746
51	27	0	0.384893	0.701278	0.346608
52	7	0	-4.079268	0.766532	-0.180349
53	7	0	-3.208703	-1.863229	-0.685875
54	6	0	-5.486199	-2.561963	-0.977515
55	1	0	-5.130240	-3.565633	-1.185942
56	6	0	-6.821872	-2.254863	-0.965665
57	1	0	-7.557183	-3.025492	-1.172034
58	6	0	-7.261540	-0.935580	-0.682482
59	6	0	-6.359634	0.061366	-0.417009
60	1	0	-6.672922	1.077693	-0.201868
61	1	0	-8.325079	-0.720657	-0.678652
62	1	0	0.954614	1.460232	-0.772707

63	1	0	1.660979	2.807441	0.925415
64	1	0	2.415038	2.404826	-0.629101
65	6	0	3.737077	0.370998	0.233428
66	6	0	4.808322	0.039717	-0.215757
67	6	0	6.074678	-0.342380	-0.768991
68	6	0	7.062478	0.625652	-1.000539
69	6	0	6.332202	-1.686259	-1.075411
70	6	0	8.290447	0.249199	-1.532110
71	1	0	6.860883	1.665312	-0.764193
72	6	0	7.562388	-2.052143	-1.609090
73	1	0	5.566358	-2.433451	-0.894538
74	6	0	8.542086	-1.087312	-1.836816
75	1	0	9.052451	1.001352	-1.709005
76	1	0	7.757155	-3.093368	-1.844789
77	1	0	9.501639	-1.377073	-2.253221
78	1	0	1.812991	-0.179399	0.582299

15(β)

E(RwB97XD/6-311G++(d, p)) = -3472.625245 a. u.

ΔG(RwB97XD/6-31G(d, p)) = 0.580706 a. u.

Standard orientation:

Center Atomic Atomic Coordinates (Angstroms)
Number Number Type X Y Z
----
1 6 0 3.386730 0.269243 -0.259003
2 6 0 3.039685 -0.986860 0.319677
3 6 0 5.136662 -1.766578 -0.122595
4 6 0 5.450306 -0.547345 -0.783197
5 15 0 2.146244 1.636508 -0.139887
6 15 0 1.303809 -1.201369 0.870245
7 6 0 1.805869 2.022764 -1.886463

8 1 0 1.248817 2.958825 -1.960832
9 1 0 2.740873 2.108442 -2.444934
10 1 0 1.209301 1.213738 -2.312872
11 6 0 1.476383 -2.069849 2.459514
12 6 0 3.106037 3.092369 0.549715
13 6 0 3.893559 2.638443 1.786538
14 1 0 3.242516 2.194966 2.546022
15 1 0 4.673744 1.915521 1.531199
16 1 0 4.381637 3.512000 2.231727
17 6 0 2.126238 4.207936 0.938996
18 1 0 2.704005 5.102777 1.195521
19 1 0 1.452692 4.468650 0.115684
20 1 0 1.519393 3.934727 1.804915
21 6 0 4.079596 3.647923 -0.504725
22 1 0 3.549413 4.079399 -1.358636
23 1 0 4.663795 4.449784 -0.040236
24 1 0 4.771711 2.885992 -0.868117
25 6 0 0.574720 -2.394192 -0.366937
26 6 0 -0.850094 -2.712034 0.111648
27 1 0 -0.845902 -3.246652 1.067093
28 1 0 -1.451127 -1.806385 0.221085
29 1 0 -1.339499 -3.357147 -0.626637
30 6 0 1.370469 -3.695530 -0.509020
31 1 0 0.839969 -4.354676 -1.205946
32 1 0 2.373744 -3.523567 -0.906028
33 1 0 1.464540 -4.226979 0.442646
34 6 0 0.528264 -1.658465 -1.714102
35 1 0 1.531156 -1.389271 -2.063772
36 1 0 0.076505 -2.311567 -2.468734
37 1 0 -0.071280 -0.745173 -1.652741
38 1 0 0.487851 -2.236728 2.894272
39 1 0 1.992246 -3.026115 2.348628
40 1 0 2.049946 -1.429315 3.133985
41 27 0 0.510523 0.893688 0.958963
42 7 0 4.551408 0.471296 -0.827091
43 7 0 3.902601 -1.972707 0.409638
44 6 0 6.113716 -2.789617 -0.041982
45 1 0 5.857805 -3.710602 0.471420
46 6 0 7.343013 -2.598607 -0.617279
47 1 0 8.094813 -3.379000 -0.562317
48 6 0 7.649071 -1.388706 -1.292720
49 6 0 6.725516 -0.379730 -1.377503
50 1 0 6.941981 0.556648 -1.881154
51 1 0 8.628402 -1.265746 -1.743212
52 6 0 -2.093197 1.201241 -0.458591
53 6 0 -1.675658 2.155586 -1.527260
54 1 0 -2.518340 2.680120 -1.977650
55 1 0 -0.979805 2.875166 -1.088204
56 1 0 -1.139288 1.597225 -2.299741
57 7 0 -3.406597 1.001522 -0.285701

58 1 0 -4.021029 1.496813 -0.919693
59 6 0 -4.056967 0.156414 0.649936
60 6 0 -5.454243 0.007416 0.371442
61 6 0 -6.633181 -0.102504 0.119365
62 6 0 -3.483420 -0.452389 1.698221
63 1 0 -2.434912 -0.347299 1.931678
64 6 0 -8.029347 -0.253929 -0.158092
65 6 0 -8.603638 0.380005 -1.270042
66 6 0 -8.829443 -1.042071 0.682776
67 6 0 -9.959320 0.223336 -1.532878
68 1 0 -7.983562 0.990134 -1.918659
69 6 0 -10.183757 -1.192016 0.410279
70 1 0 -8.383212 -1.530263 1.542940
71 6 0 -10.750745 -0.561851 -0.696212
72 1 0 -10.399378 0.714167 -2.394984
73 1 0 -10.798508 -1.803832 1.062570
74 1 0 -11.808761 -0.683040 -0.905846
75 1 0 -4.101799 -1.073619 2.334717
76 1 0 0.196044 2.342448 1.074836
77 1 0 1.522590 1.060378 1.993753
78 8 0 -1.252854 0.586149 0.231740

TS6

E(RwB97XD/6-311G++(d, p)) = -3472.624369 a. u.

ΔG(RwB97XD/6-31G(d, p)) = 0.582098 a. u.

$\nu = i25.01$

Standard orientation:

Center Atomic Atomic Coordinates (Angstroms)
Number Number Type X Y Z
----
1 6 0 3.348272 0.312988 -0.223805
2 6 0 3.046491 -0.962573 0.337769
3 6 0 5.171084 -1.659332 -0.117650
4 6 0 5.439954 -0.420532 -0.760885
5 15 0 2.055506 1.628136 -0.099730
6 15 0 1.310472 -1.266298 0.854944
7 6 0 1.749497 2.052411 -1.843438
8 1 0 1.196052 2.990877 -1.908148
9 1 0 2.697785 2.152512 -2.376945
10 1 0 1.165236 1.251369 -2.302697
11 6 0 1.518432 -2.126422 2.446133
12 6 0 2.937163 3.097495 0.659144
13 6 0 3.683072 2.638588 1.919103
14 1 0 3.012361 2.162826 2.640861

15 1 0 4.491243 1.940012 1.682855
16 1 0 4.130677 3.514408 2.400903
17 6 0 1.904764 4.172641 1.024525
18 1 0 2.440474 5.075457 1.337432
19 1 0 1.272910 4.438440 0.169781
20 1 0 1.257584 3.856304 1.845048
21 6 0 3.935703 3.705416 -0.341262
22 1 0 3.426445 4.152401 -1.199935
23 1 0 4.486130 4.504889 0.167112
24 1 0 4.655794 2.969744 -0.703914
25 6 0 0.681484 -2.509516 -0.390071
26 6 0 -0.787961 -2.790282 -0.043653
27 1 0 -0.902158 -3.221191 0.956507
28 1 0 -1.391260 -1.882175 -0.102420
29 1 0 -1.190891 -3.513850 -0.761546
30 6 0 1.471787 -3.822229 -0.381845
31 1 0 1.064300 -4.485552 -1.153645
32 1 0 2.532583 -3.666286 -0.591735
33 1 0 1.383867 -4.339121 0.578703
34 6 0 0.768268 -1.836279 -1.768210
35 1 0 1.804407 -1.636120 -2.061400
36 1 0 0.332665 -2.500504 -2.522904
37 1 0 0.211177 -0.892937 -1.785497
38 1 0 0.567719 -2.550802 2.777817
39 1 0 2.269564 -2.916108 2.371911
40 1 0 1.850824 -1.391910 3.184275
41 27 0 0.415307 0.790115 0.936370
42 7 0 4.505399 0.565354 -0.787452
43 7 0 3.946784 -1.915118 0.414473
44 6 0 6.180117 -2.652196 -0.059930
45 1 0 5.956478 -3.589275 0.439144
46 6 0 7.397684 -2.413807 -0.642112
47 1 0 8.172730 -3.172241 -0.607270
48 6 0 7.659862 -1.184070 -1.299879
49 6 0 6.704741 -0.203219 -1.361713
50 1 0 6.887549 0.746665 -1.853435
51 1 0 8.630847 -1.024519 -1.757021
52 6 0 -2.061027 1.249828 -0.597901
53 6 0 -1.687066 2.389279 -1.487152
54 1 0 -2.548111 2.972117 -1.812682
55 1 0 -0.995391 3.029267 -0.934336
56 1 0 -1.158820 1.993505 -2.358431
57 7 0 -3.364088 1.043382 -0.366695
58 1 0 -4.011670 1.643405 -0.863641
59 6 0 -3.962890 0.093179 0.502195
60 6 0 -5.371296 -0.039476 0.277603
61 6 0 -6.562392 -0.132494 0.081900
62 6 0 -3.336175 -0.612019 1.455009
63 1 0 -2.278829 -0.520795 1.649177
64 6 0 -7.970881 -0.265136 -0.136391

65 6 0 -8.584605 0.394175 -1.212055
66 6 0 -8.743362 -1.058642 0.724851
67 6 0 -9.951915 0.256868 -1.419454
68 1 0 -7.985714 1.010236 -1.874802
69 6 0 -10.109344 -1.190961 0.506475
70 1 0 -8.266727 -1.566476 1.556901
71 6 0 -10.715551 -0.535664 -0.564020
72 1 0 -10.422746 0.768166 -2.252905
73 1 0 -10.702539 -1.808768 1.172924
74 1 0 -11.782577 -0.642882 -0.731290
75 1 0 -3.919376 -1.297474 2.057696
76 1 0 0.033698 2.210307 1.145974
77 1 0 1.343080 0.961047 2.049870
78 8 0 -1.195058 0.496365 -0.100063

19

E(RwB97XD/6-311G++(d, p)) = -3472.657818 a. u.

ΔG(RwB97XD/6-31G(d, p)) = 0.585438 a. u.

Standard orientation:

Center Number	Atomic Number	Atomic Type	Coordinates (Angstroms) X	Y	Z
1	6	0	2.076697	-0.946870	0.459377
2	6	0	2.448646	-0.155130	-0.664351
3	6	0	4.345131	-1.397402	-0.905292
4	6	0	4.011951	-2.134695	0.264491
5	15	0	0.418838	-0.619507	1.195462
6	15	0	1.380610	1.288914	-1.062576
7	6	0	0.779233	-0.232175	2.933789
8	1	0	-0.158083	-0.172404	3.492263
9	1	0	1.429113	-0.989297	3.377235
10	1	0	1.275049	0.740002	2.975066
11	6	0	1.265710	1.225638	-2.875317
12	6	0	-0.444445	-2.284366	1.142466

13	6	0	-0.262688	-2.894794	-0.254452
14	1	0	-0.616064	-2.222220	-1.042309
15	1	0	0.781050	-3.151711	-0.457248
16	1	0	-0.849857	-3.817424	-0.312565
17	6	0	-1.940337	-2.077777	1.419758
18	1	0	-2.419990	-3.059138	1.503509
19	1	0	-2.114112	-1.541490	2.359109
20	1	0	-2.431242	-1.531735	0.609421
21	6	0	0.138237	-3.231284	2.203314
22	1	0	-0.062026	-2.873825	3.217515
23	1	0	-0.341680	-4.209997	2.094652
24	1	0	1.215800	-3.365070	2.084281
25	6	0	2.463007	2.752289	-0.622651
26	6	0	1.607600	4.022753	-0.726782
27	1	0	1.173041	4.142283	-1.724704
28	1	0	0.803594	4.022341	0.012488
29	1	0	2.243729	4.894011	-0.534960
30	6	0	3.667014	2.870706	-1.565236
31	1	0	4.295726	3.702196	-1.226563
32	1	0	4.277749	1.964223	-1.572051
33	1	0	3.358121	3.086758	-2.592351
34	6	0	2.939053	2.565621	0.825221
35	1	0	3.611727	1.708197	0.928546
36	1	0	3.493114	3.459261	1.133396
37	1	0	2.096734	2.440946	1.511789
38	1	0	0.842501	2.159942	-3.252081
39	1	0	2.249166	1.067116	-3.322967
40	1	0	0.604953	0.400796	-3.151956
41	27	0	-0.492153	0.983387	0.076342
42	7	0	2.851327	-1.895491	0.930606

43	7	0	3.545312	-0.390836	-1.346467
44	6	0	5.539622	-1.698322	-1.605521
45	1	0	5.773452	-1.127943	-2.498429
46	6	0	6.366956	-2.686532	-1.139736
47	1	0	7.285316	-2.918601	-1.668943
48	6	0	6.041223	-3.412988	0.035201
49	6	0	4.888633	-3.147288	0.727376
50	1	0	4.618796	-3.695491	1.624110
51	1	0	6.716515	-4.187625	0.383162
52	6	0	-1.408935	3.023792	1.672064
53	6	0	-1.526261	4.086109	2.712556
54	1	0	-2.565614	4.348430	2.912679
55	1	0	-1.044667	3.742753	3.629742
56	1	0	-0.994770	4.975342	2.360705
57	7	0	-2.463113	2.728991	0.911901
58	1	0	-3.346882	3.207211	1.035493
59	6	0	-2.291930	1.818814	-0.197750
60	6	0	-3.404451	0.940379	-0.436888
61	6	0	-4.325924	0.177041	-0.616790
62	6	0	-1.412693	2.219969	-1.223080
63	1	0	-1.020639	3.231161	-1.193847
64	6	0	-5.386457	-0.770455	-0.791240
65	6	0	-5.771260	-1.585465	0.284397
66	6	0	-6.031697	-0.905207	-2.028837
67	6	0	-6.786725	-2.519789	0.119291
68	1	0	-5.268939	-1.480212	1.240786
69	6	0	-7.043425	-1.845932	-2.185303
70	1	0	-5.733506	-0.273866	-2.859564
71	6	0	-7.422689	-2.652825	-1.113995
72	1	0	-7.080492	-3.148107	0.954061

73	1	0	-7.537274	-1.949328	-3.146147
74	1	0	-8.212533	-3.386596	-1.240026
75	1	0	-1.520417	1.777139	-2.206337
76	1	0	-1.455087	0.353388	0.971309
77	1	0	-0.709922	-0.083703	-0.884116
78	8	0	-0.326563	2.416026	1.484071

TS9

E(RwB97XD/6-311G++(d, p)) = -3472.655638 a. u.

ΔG(RwB97XD/6-31G(d, p)) = 0.583270 a. u.

ν = *i* 436.74

Standard orientation:

Center Number	Atomic Number	Atomic Type	Coordinates (Angstroms) X	Y	Z
1	6	0	2.202368	-0.906593	0.410255
2	6	0	2.501065	-0.085719	-0.716899
3	6	0	4.443262	-1.238380	-1.037038
4	6	0	4.178836	-2.007180	0.129711
5	15	0	0.570116	-0.660366	1.226341
6	15	0	1.347738	1.305240	-1.071178
7	6	0	1.003912	-0.247236	2.943971
8	1	0	0.093175	-0.215930	3.547576
9	1	0	1.701133	-0.976241	3.362308
10	1	0	1.463718	0.743149	2.954687
11	6	0	1.164545	1.247334	-2.877858
12	6	0	-0.219133	-2.358968	1.237703
13	6	0	-0.064274	-2.987841	-0.153958
14	1	0	-0.454801	-2.336152	-0.942033

15	1	0	0.979802	-3.222435	-0.381033
16	1	0	-0.630522	-3.924970	-0.182262
17	6	0	-1.710321	-2.193320	1.565099
18	1	0	-2.155883	-3.185896	1.694286
19	1	0	-1.867765	-1.633811	2.493620
20	1	0	-2.246770	-1.687441	0.756924
21	6	0	0.432422	-3.263790	2.294385
22	1	0	0.249527	-2.896957	3.308591
23	1	0	-0.010221	-4.263042	2.217490
24	1	0	1.510691	-3.355500	2.143979
25	6	0	2.355670	2.828391	-0.665778
26	6	0	1.414441	4.039618	-0.734932
27	1	0	0.940176	4.132803	-1.717511
28	1	0	0.635503	3.982075	0.029114
29	1	0	1.995683	4.951630	-0.559304
30	6	0	3.512704	3.021329	-1.653707
31	1	0	4.095924	3.896233	-1.344060
32	1	0	4.182756	2.157842	-1.677580
33	1	0	3.150172	3.207204	-2.669174
34	6	0	2.895940	2.672226	0.762608
35	1	0	3.618017	1.853027	0.840138
36	1	0	3.411935	3.595406	1.049024
37	1	0	2.088381	2.503497	1.480952
38	1	0	0.673766	2.159495	-3.225547
39	1	0	2.135677	1.146225	-3.367029
40	1	0	0.539504	0.389512	-3.136173
41	27	0	-0.456986	0.904124	0.109581
42	7	0	3.033018	-1.827969	0.837948
43	7	0	3.584679	-0.262727	-1.437089
44	6	0	5.627878	-1.477100	-1.777257

45	1	0	5.810421	-0.884340	-2.667554
46	6	0	6.510458	-2.435610	-1.351897
47	1	0	7.421826	-2.620001	-1.911096
48	6	0	6.252247	-3.193126	-0.179937
49	6	0	5.110893	-2.987954	0.549824
50	1	0	4.891393	-3.559283	1.445774
51	1	0	6.970225	-3.942764	0.135504
52	6	0	-1.450337	2.804391	1.843907
53	6	0	-1.586054	3.819645	2.927967
54	1	0	-2.628770	4.056943	3.140256
55	1	0	-1.100359	3.442088	3.830089
56	1	0	-1.064251	4.729510	2.618257
57	7	0	-2.527840	2.426069	1.156589
58	1	0	-3.440688	2.819693	1.346679
59	6	0	-2.346389	1.552678	0.012802
60	6	0	-3.483969	0.710117	-0.270284
61	6	0	-4.440881	0.004417	-0.490533
62	6	0	-1.549341	2.085174	-1.047328
63	1	0	-1.277421	3.134325	-0.982682
64	6	0	-5.583577	-0.814497	-0.772169
65	6	0	-5.743183	-2.053308	-0.134087
66	6	0	-6.549346	-0.376370	-1.689790
67	6	0	-6.855013	-2.839257	-0.414939
68	1	0	-4.994926	-2.390971	0.576118
69	6	0	-7.658055	-1.168495	-1.962910
70	1	0	-6.422965	0.582342	-2.182094
71	6	0	-7.812511	-2.399270	-1.327345
72	1	0	-6.974921	-3.797475	0.080301
73	1	0	-8.402433	-0.824917	-2.673884
74	1	0	-8.679526	-3.015599	-1.543427

75	1	0	-1.710504	1.703021	-2.049060
76	1	0	-1.571615	0.322307	0.860126
77	1	0	-0.633153	-0.154853	-0.868939
78	8	0	-0.334455	2.305045	1.557859

23

E(RwB97XD/6-311G++(d, p)) = -3472.660724 a. u.

ΔG(RwB97XD/6-31G(d, p)) = 0.588200 a. u.

Standard orientation:

Center Number	Atomic Number	Atomic Type	Coordinates (Angstroms) X	Y	Z
1	6	0	-2.702448	0.693842	0.036387
2	6	0	-2.528795	-0.568079	-0.608706
3	6	0	-4.793406	-0.768106	-0.812154
4	6	0	-4.966406	0.472308	-0.139295
5	15	0	-1.188065	1.640904	0.504635
6	15	0	-0.805248	-1.192579	-0.797968
7	6	0	-1.547846	2.178164	2.203910
8	1	0	-0.805688	2.916960	2.515440
9	1	0	-2.548678	2.608712	2.276867
10	1	0	-1.477086	1.308950	2.861254
11	6	0	-0.573334	-1.181604	-2.605586
12	6	0	-1.223185	3.161216	-0.579898
13	6	0	-1.385085	2.722641	-2.040318
14	1	0	-0.572916	2.060455	-2.353846
15	1	0	-2.339876	2.216126	-2.213335

16	1	0	-1.359036	3.610319	-2.681657
17	6	0	0.107957	3.902767	-0.401665
18	1	0	0.053786	4.857412	-0.936298
19	1	0	0.316743	4.119999	0.651127
20	1	0	0.943490	3.330802	-0.811723
21	6	0	-2.381293	4.086933	-0.180859
22	1	0	-2.243921	4.498581	0.823231
23	1	0	-2.410151	4.927400	-0.883013
24	1	0	-3.347867	3.578544	-0.219136
25	6	0	-0.832311	-2.992960	-0.289761
26	6	0	0.639124	-3.404331	-0.118186
27	1	0	1.218753	-3.224995	-1.030641
28	1	0	1.121345	-2.870006	0.705763
29	1	0	0.690118	-4.476327	0.101973
30	6	0	-1.488450	-3.892874	-1.344782
31	1	0	-1.522572	-4.917917	-0.957831
32	1	0	-2.511137	-3.577194	-1.565017
33	1	0	-0.917914	-3.910749	-2.278058
34	6	0	-1.578650	-3.123365	1.044995
35	1	0	-2.653286	-2.962830	0.917670
36	1	0	-1.436388	-4.137008	1.436410
37	1	0	-1.207400	-2.413938	1.788949
38	1	0	0.391192	-1.637777	-2.843924
39	1	0	-1.372425	-1.722685	-3.117145
40	1	0	-0.563157	-0.146161	-2.951385
41	8	0	0.153202	-0.597780	2.147544
42	6	0	1.176625	-0.995527	2.757474
43	7	0	2.380190	-0.780929	2.228506
44	6	0	2.466866	0.011267	1.003264
45	6	0	1.814683	1.361847	1.144915

46	1	0	3.224355	-1.083061	2.696458
47	6	0	1.058159	-1.735001	4.047528
48	1	0	0.423854	-1.159553	4.725152
49	1	0	0.565091	-2.692013	3.854054
50	1	0	2.027729	-1.913732	4.513053
51	27	0	0.455670	0.268056	0.351465
52	7	0	-3.892389	1.193800	0.276275
53	7	0	-3.551594	-1.273152	-1.034449
54	6	0	-5.930600	-1.494787	-1.244664
55	1	0	-5.776574	-2.440935	-1.753030
56	6	0	-7.183770	-0.991787	-1.010984
57	1	0	-8.059102	-1.542203	-1.339725
58	6	0	-7.356246	0.246970	-0.340257
59	6	0	-6.273652	0.968046	0.091249
60	1	0	-6.384045	1.917936	0.603846
61	1	0	-8.359856	0.623006	-0.171277
62	1	0	0.732492	0.875601	-0.943068
63	1	0	1.688802	1.663894	2.184966
64	1	0	2.278513	2.140767	0.546855
65	6	0	3.809473	0.009130	0.444591
66	6	0	4.933907	0.010646	0.002370
67	6	0	6.267753	0.061467	-0.521629
68	6	0	6.986857	1.265242	-0.485986
69	6	0	6.859057	-1.084659	-1.071816
70	6	0	8.278067	1.318311	-0.997196
71	1	0	6.527487	2.150334	-0.058119
72	6	0	8.151697	-1.021865	-1.579513
73	1	0	6.302384	-2.015759	-1.098193
74	6	0	8.861624	0.176990	-1.544465
75	1	0	8.828981	2.252869	-0.968556

76	1	0	8.605241	-1.910878	-2.005656
77	1	0	9.869436	0.222358	-1.944986
78	1	0	1.878594	-0.640525	0.206516

TS12

E(RwB97XD/6-311G++(d, p))= -3472.631629 a. u.

ΔG(RwB97XD/6-31G(d, p)) = 0.580023 a. u.

$\nu = i859.83$

Standard orientation:

Center Atomic Atomic Coordinates (Angstroms)
Number Number Type X Y Z
----
1 6 0 -3.110991 0.251183 -0.006630
2 6 0 -2.594701 -1.071842 -0.140534
3 6 0 -4.681454 -1.837312 -0.639844
4 6 0 -5.209835 -0.534168 -0.429452
5 15 0 -1.861616 1.579500 0.250626
6 15 0 -0.816261 -1.304205 0.265283
7 6 0 -2.542506 2.530245 1.651713
8 1 0 -1.990809 3.468197 1.754691
9 1 0 -3.604427 2.747721 1.515684
10 1 0 -2.409967 1.948219 2.567494
11 6 0 -0.256110 -2.484960 -1.001246
12 6 0 -2.069479 2.664405 -1.268422
13 6 0 -2.003829 1.757756 -2.505609
14 1 0 -1.093560 1.147656 -2.510480
15 1 0 -2.868545 1.089941 -2.570819
16 1 0 -1.995653 2.379255 -3.408066
17 6 0 -0.896245 3.655157 -1.295212
18 1 0 -1.028847 4.345475 -2.136097
19 1 0 -0.845268 4.249064 -0.376967
20 1 0 0.060908 3.141593 -1.420844
21 6 0 -3.392058 3.439867 -1.252750
22 1 0 -3.420639 4.169986 -0.438356
23 1 0 -3.491695 3.990971 -2.195161
24 1 0 -4.253889 2.775048 -1.153531
25 6 0 -0.866415 -2.229568 1.890697
26 6 0 0.578635 -2.323636 2.402419
27 1 0 1.246940 -2.777282 1.662710
28 1 0 0.976603 -1.338757 2.664252
29 1 0 0.603799 -2.945648 3.304200
30 6 0 -1.453897 -3.636891 1.740715

31 1 0 -1.525535 -4.099923 2.731683
32 1 0 -2.454816 -3.619181 1.301357
33 1 0 -0.817064 -4.275474 1.120756
34 6 0 -1.709400 -1.400547 2.870794
35 1 0 -2.763223 -1.363879 2.576496
36 1 0 -1.656253 -1.856517 3.865472
37 1 0 -1.337411 -0.372940 2.953980
38 1 0 0.713441 -2.901583 -0.717692
39 1 0 -0.975779 -3.296744 -1.127108
40 1 0 -0.146105 -1.953345 -1.949835
41 27 0 0.144807 0.645070 0.408056
42 7 0 -4.393323 0.509792 -0.119925
43 7 0 -3.355427 -2.088669 -0.471299
44 6 0 -5.549939 -2.894549 -1.007781
45 1 0 -5.124363 -3.880092 -1.165785
46 6 0 -6.891739 -2.652499 -1.146962
47 1 0 -7.562326 -3.457859 -1.427939
48 6 0 -7.421176 -1.355044 -0.925805
49 6 0 -6.602175 -0.313890 -0.572909
50 1 0 -6.987661 0.686814 -0.407381
51 1 0 -8.487094 -1.190173 -1.043101
52 6 0 2.061869 2.769437 1.008951
53 6 0 2.330696 4.028851 1.774955
54 1 0 3.394385 4.257126 1.852679
55 1 0 1.819935 4.854456 1.271352
56 1 0 1.902583 3.929380 2.774787
57 7 0 3.088477 2.183125 0.405458
58 1 0 3.984070 2.650885 0.457548
59 6 0 3.021298 0.990648 -0.448307
60 6 0 4.362801 0.401268 -0.511481
61 6 0 5.457278 -0.107958 -0.574209
62 6 0 1.988099 -0.024375 0.060480
63 1 0 2.174071 -0.207729 1.133372
64 6 0 6.750672 -0.720916 -0.671661
65 6 0 7.785560 -0.340350 0.194697
66 6 0 6.987738 -1.705195 -1.642341
67 6 0 9.036495 -0.937927 0.087499
68 1 0 7.603278 0.421059 0.946101
69 6 0 8.241466 -2.297374 -1.740926
70 1 0 6.187554 -1.999424 -2.313675
71 6 0 9.267473 -1.915659 -0.878425
72 1 0 9.832819 -0.638488 0.761410
73 1 0 8.417327 -3.058462 -2.494414
74 1 0 10.245208 -2.379848 -0.959432
75 1 0 2.171127 -0.976286 -0.444204
76 1 0 0.860412 0.334144 -0.798994
77 8 0 0.883896 2.336166 0.964484
78 1 0 2.746776 1.312322 -1.462300

16(α)

E(RwB97XD/6-311G++(d, p)) -3472.631505 a. u.

ΔG(RwB97XD/6-31G(d, p)) = 0.579917 a. u.

Standard orientation:

Center Atomic Atomic Coordinates (Angstroms)
Number Number Type X Y Z
----
1 6 0 2.646152 -0.114997 0.164799
2 6 0 1.757372 0.998543 0.100484
3 6 0 3.532766 2.377682 -0.296483
4 6 0 4.423035 1.278104 -0.157062
5 15 0 1.880515 -1.781670 0.326072
6 15 0 -0.032830 0.690001 0.419949
7 6 0 2.784864 -2.531970 1.714127
8 1 0 2.577027 -3.604118 1.747440
9 1 0 3.860171 -2.367154 1.615934
10 1 0 2.436576 -2.076092 2.644825
11 6 0 -0.807921 1.519605 -1.000833
12 6 0 2.396583 -2.678588 -1.232811
13 6 0 2.046884 -1.798392 -2.440776
14 1 0 0.979176 -1.559319 -2.474996
15 1 0 2.614228 -0.862328 -2.446340
16 1 0 2.296717 -2.342633 -3.357962
17 6 0 1.625062 -4.002876 -1.315034
18 1 0 1.996826 -4.571913 -2.174213
19 1 0 1.770320 -4.613470 -0.417966
20 1 0 0.553285 -3.837609 -1.450689
21 6 0 3.903900 -2.973950 -1.222878
22 1 0 4.174924 -3.669624 -0.423247
23 1 0 4.172114 -3.444125 -2.175422
24 1 0 4.499591 -2.065227 -1.110070
25 6 0 -0.424990 1.700315 1.939016
26 6 0 -1.918030 1.487726 2.235464
27 1 0 -2.549615 1.899505 1.441400
28 1 0 -2.165376 0.429906 2.356686
29 1 0 -2.173631 2.009464 3.164724
30 6 0 -0.147368 3.197462 1.778900
31 1 0 -0.435972 3.710660 2.703715
32 1 0 0.908656 3.401159 1.589841
33 1 0 -0.732072 3.630389 0.960965
34 6 0 0.427638 1.112896 3.073242
35 1 0 1.498288 1.254677 2.890536
36 1 0 0.181412 1.615251 4.015111
37 1 0 0.241064 0.040607 3.206696

38 1 0 -1.864998 1.692010 -0.796191
39 1 0 -0.322153 2.478989 -1.196307
40 1 0 -0.717680 0.880817 -1.882422
41 8 0 -2.071682 -1.646815 1.224007
42 6 0 -2.749625 -2.699526 1.123551
43 7 0 -3.176620 -3.145700 -0.057963
44 6 0 -3.017111 -2.439327 -1.290525
45 6 0 -2.503047 -3.050446 -2.360524
46 1 0 -2.441939 -2.535674 -3.311946
47 1 0 -3.662614 -4.033957 -0.090375
48 6 0 -3.129219 -3.467359 2.346792
49 1 0 -2.215045 -3.745402 2.877747
50 1 0 -3.708800 -2.808065 2.998884
51 1 0 -3.713948 -4.360275 2.123803
52 1 0 -2.152333 -4.075041 -2.297073
53 6 0 -3.509522 -1.096722 -1.287759
54 6 0 -3.965674 0.022357 -1.217079
55 6 0 -4.482260 1.350026 -1.084650
56 6 0 -4.280263 2.303043 -2.093784
57 6 0 -5.165548 1.712157 0.086889
58 6 0 -4.759294 3.597595 -1.929390
59 1 0 -3.743890 2.023944 -2.994846
60 6 0 -5.640113 3.009039 0.240620
61 1 0 -5.312150 0.974349 0.868980
62 6 0 -5.438979 3.952768 -0.765484
63 1 0 -4.599909 4.332552 -2.711791
64 1 0 -6.166277 3.283851 1.149120
65 1 0 -5.810834 4.964913 -0.641557
66 27 0 -0.216048 -1.534725 0.658368
67 7 0 3.948011 0.024473 0.063898
68 7 0 2.191817 2.211836 -0.154055
69 6 0 4.052855 3.668334 -0.566124
70 1 0 3.356092 4.493741 -0.669067
71 6 0 5.407148 3.842212 -0.681562
72 1 0 5.812392 4.827958 -0.884670
73 6 0 6.295926 2.745230 -0.535443
74 6 0 5.819517 1.486493 -0.277499
75 1 0 6.481298 0.633469 -0.168851
76 1 0 7.363497 2.911529 -0.634152
77 1 0 -0.119045 -3.035696 0.710119
78 1 0 -0.300760 -1.573757 -0.766957

TS7

E(RwB97XD/6-311G++(d, p)) -3472.624854 a. u.

ΔG(RwB97XD/6-31G(d, p)) = 0.579917 a. u.

$\nu = i80.63$

Standard orientation:

Center Atomic Atomic Coordinates (Angstroms)
Number Number Type X Y Z

----

1 6 0 2.607448 0.280907 0.119022
2 6 0 1.484591 1.143881 -0.054677
3 6 0 2.881542 2.948417 -0.040854
4 6 0 3.982025 2.096947 0.247317
5 15 0 2.288104 -1.534936 -0.026569
6 15 0 -0.195303 0.387691 -0.004905
7 6 0 2.847686 -2.219857 1.563915
8 1 0 2.901595 -3.308638 1.490298
9 1 0 3.825264 -1.819761 1.841528
10 1 0 2.119666 -1.952172 2.332835
11 6 0 -1.100640 1.366703 -1.242958
12 6 0 3.521380 -2.091766 -1.330273
13 6 0 3.454972 -1.137732 -2.531381
14 1 0 2.445221 -1.071788 -2.947979
15 1 0 3.791044 -0.128730 -2.276607
16 1 0 4.117510 -1.518020 -3.316108
17 6 0 3.185230 -3.515627 -1.792824
18 1 0 3.980995 -3.857560 -2.463941
19 1 0 3.129100 -4.217885 -0.954604
20 1 0 2.239816 -3.554568 -2.339518
21 6 0 4.946181 -2.091682 -0.748569
22 1 0 5.063475 -2.850305 0.030793
23 1 0 5.647511 -2.335600 -1.553902
24 1 0 5.222865 -1.119264 -0.336437
25 6 0 -0.804054 0.920992 1.688881
26 6 0 -2.264030 0.473739 1.831115
27 1 0 -2.917634 1.028106 1.152759
28 1 0 -2.398750 -0.592943 1.641093
29 1 0 -2.598582 0.675893 2.854893
30 6 0 -0.738471 2.437744 1.904080
31 1 0 -1.175548 2.667718 2.882727
32 1 0 0.285748 2.815942 1.896128
33 1 0 -1.312166 2.983416 1.148617
34 6 0 0.063674 0.203587 2.732891
35 1 0 1.120680 0.478226 2.647606
36 1 0 -0.272129 0.488446 3.736234
37 1 0 -0.021779 -0.882876 2.639931
38 1 0 -2.171666 1.172421 -1.159942
39 1 0 -0.914311 2.435552 -1.117397
40 1 0 -0.763600 1.056661 -2.235428
41 8 0 -0.697341 -2.630598 1.097192
42 6 0 -1.808670 -3.113521 1.376597

43 7 0 -2.867917 -3.122077 0.547925
44 6 0 -2.974062 -2.512955 -0.747047
45 6 0 -2.515547 -3.118661 -1.848347
46 1 0 -2.631771 -2.653841 -2.820606
47 1 0 -3.736480 -3.471780 0.935643
48 6 0 -2.011653 -3.710323 2.736950
49 1 0 -1.209069 -4.424784 2.930811
50 1 0 -1.932605 -2.906473 3.474841
51 1 0 -2.976976 -4.205033 2.849432
52 1 0 -2.011936 -4.077300 -1.781228
53 6 0 -3.716827 -1.291299 -0.787349
54 6 0 -4.369890 -0.271735 -0.796806
55 6 0 -5.087950 0.965073 -0.770445
56 6 0 -5.091178 1.808479 -1.891443
57 6 0 -5.766455 1.352733 0.395189
58 6 0 -5.765694 3.022759 -1.840735
59 1 0 -4.562644 1.508179 -2.790252
60 6 0 -6.438995 2.568061 0.433956
61 1 0 -5.756564 0.698924 1.261310
62 6 0 -6.439917 3.403922 -0.681764
63 1 0 -5.764112 3.674711 -2.708353
64 1 0 -6.961966 2.863936 1.337622
65 1 0 -6.963713 4.353840 -0.647107
66 27 0 0.257262 -1.775619 -0.389069
67 7 0 3.818871 0.748071 0.303569
68 7 0 1.625028 2.443704 -0.165706
69 6 0 3.089742 4.344369 -0.162240
70 1 0 2.237351 4.976032 -0.390179
71 6 0 4.346777 4.859737 0.019977
72 1 0 4.512906 5.928133 -0.069594
73 6 0 5.441515 4.011703 0.329538
74 6 0 5.269061 2.656466 0.441636
75 1 0 6.094196 1.989016 0.667882
76 1 0 6.424677 4.447538 0.473121
77 1 0 0.994196 -3.077132 -0.569160
78 1 0 0.742195 -1.255314 -1.668955

24

E(RwB97XD/6-311G++(d, p)) -3472.658666 a. u.

ΔG(RwB97XD/6-31G(d, p)) = 0.579917 a. u.

Standard orientation:

Center Atomic Atomic Coordinates (Angstroms)
Number Number Type X Y Z

----
1 6 0 -2.693047 0.665541 0.045177
2 6 0 -2.485249 -0.571814 -0.632187
3 6 0 -4.733013 -0.755519 -0.982971
4 6 0 -4.945949 0.449340 -0.258151
5 15 0 -1.200197 1.612021 0.576353
6 15 0 -0.759943 -1.211090 -0.706082
7 6 0 -1.609447 2.136804 2.268543
8 1 0 -0.842266 2.826339 2.629045
9 1 0 -2.587897 2.619004 2.310916
10 1 0 -1.614049 1.248308 2.905153
11 6 0 -0.427273 -1.265834 -2.496550
12 6 0 -1.221176 3.121641 -0.528935
13 6 0 -1.402069 2.655790 -1.980509
14 1 0 -0.650366 1.911536 -2.261760
15 1 0 -2.396517 2.237146 -2.162243
16 1 0 -1.279378 3.522281 -2.639858
17 6 0 0.122241 3.849904 -0.389072
18 1 0 0.064693 4.791696 -0.945390
19 1 0 0.355417 4.091058 0.652750
20 1 0 0.943467 3.260857 -0.805426
21 6 0 -2.359272 4.075803 -0.140496
22 1 0 -2.195685 4.523098 0.843906
23 1 0 -2.391675 4.892400 -0.870457
24 1 0 -3.333177 3.580689 -0.141093
25 6 0 -0.849644 -2.985541 -0.116309
26 6 0 0.607109 -3.459563 0.054039
27 1 0 1.177645 -3.361814 -0.875828
28 1 0 1.131362 -2.902706 0.835960
29 1 0 0.610399 -4.517897 0.337549
30 6 0 -1.564571 -3.909851 -1.110632
31 1 0 -1.639310 -4.909408 -0.666694
32 1 0 -2.575177 -3.558129 -1.332418
33 1 0 -1.013478 -4.004858 -2.050932
34 6 0 -1.574158 -3.008253 1.237653
35 1 0 -2.647426 -2.830134 1.120228
36 1 0 -1.449725 -3.996527 1.693966
37 1 0 -1.170843 -2.262303 1.927663
38 1 0 0.560310 -1.703263 -2.666321
39 1 0 -1.184973 -1.845748 -3.028263
40 1 0 -0.422379 -0.242572 -2.880140
41 8 0 0.173534 -0.526467 2.277366
42 6 0 1.219522 -0.901501 2.862820
43 7 0 2.407480 -0.685980 2.295223
44 6 0 2.458748 0.070377 1.046316
45 6 0 1.821345 1.423845 1.170940
46 1 0 3.264355 -0.967205 2.751479
47 6 0 1.151225 -1.618266 4.169998
48 1 0 0.653198 -0.973895 4.898714
49 1 0 0.543467 -2.517940 4.042642

50 1 0 2.138625 -1.894773 4.541168
51 27 0 0.471627 0.257139 0.452794
52 7 0 -3.897305 1.151114 0.247381
53 7 0 -3.480815 -1.255189 -1.149990
54 6 0 -5.842851 -1.452209 -1.524362
55 1 0 -5.659450 -2.370635 -2.072719
56 6 0 -7.107563 -0.958106 -1.339896
57 1 0 -7.961362 -1.485066 -1.753174
58 6 0 -7.320848 0.241453 -0.611419
59 6 0 -6.265761 0.933801 -0.076225
60 1 0 -6.405112 1.855435 0.479236
61 1 0 -8.333215 0.609732 -0.480804
62 1 0 0.703741 0.827988 -0.862959
63 1 0 1.716951 1.732312 2.210355
64 1 0 2.251680 2.193195 0.538644
65 6 0 3.772343 0.020432 0.424584
66 6 0 4.869871 0.003640 -0.080263
67 6 0 6.178969 0.048201 -0.662301
68 6 0 6.926904 1.233471 -0.588548
69 6 0 6.724815 -1.079258 -1.289561
70 6 0 8.199829 1.286959 -1.143300
71 1 0 6.502932 2.103661 -0.097868
72 6 0 7.999256 -1.016282 -1.840652
73 1 0 6.149117 -1.997621 -1.341886
74 6 0 8.736787 0.163818 -1.770188
75 1 0 8.772935 2.206565 -1.084229
76 1 0 8.418110 -1.891854 -2.326210
77 1 0 9.730551 0.207672 -2.204932
78 1 0 1.816424 -0.614141 0.280237

TS13

E(RwB97XD/6-311G++(d, p)) = -3472.673302 a. u.

ΔG(RwB97XD/6-31G(d, p)) = 0.584578 a. u.

ν = *i*933.34

Standard orientation:

Center Atomic Atomic Coordinates (Angstroms)
Number Number Type X Y Z
----
1 6 0 -2.307908 0.381705 -0.724687
2 6 0 -2.153068 0.573099 0.677852
3 6 0 -3.847940 2.094786 0.654327
4 6 0 -3.967927 1.941024 -0.754354
5 15 0 -1.262175 -0.928893 -1.471743
6 15 0 -0.787402 -0.367953 1.478861
7 6 0 -0.680817 -0.158059 -3.021450
8 1 0 -0.203349 -0.911108 -3.653808

9 1 0 -1.502212 0.307304 -3.571026
10 1 0 0.060362 0.604107 -2.767856
11 6 0 -1.641788 -1.385280 2.724921
12 6 0 -2.494171 -2.243030 -1.990823
13 6 0 -3.305527 -2.634013 -0.748859
14 1 0 -2.653977 -2.944796 0.074941
15 1 0 -3.939837 -1.813027 -0.399011
16 1 0 -3.958912 -3.478776 -0.994071
17 6 0 -1.683927 -3.453698 -2.476796
18 1 0 -2.370841 -4.222074 -2.848870
19 1 0 -1.004169 -3.191338 -3.294856
20 1 0 -1.095720 -3.896620 -1.666181
21 6 0 -3.433862 -1.771220 -3.106108
22 1 0 -2.893362 -1.572774 -4.036461
23 1 0 -4.166805 -2.559893 -3.311838
24 1 0 -3.979202 -0.866274 -2.824941
25 6 0 0.182952 0.918238 2.436090
26 6 0 1.459489 0.246367 2.964353
27 1 0 1.242834 -0.670914 3.523404
28 1 0 2.156917 0.004035 2.160118
29 1 0 1.966118 0.939618 3.644515
30 6 0 -0.617569 1.465570 3.626554
31 1 0 -0.032406 2.259790 4.104273
32 1 0 -1.577345 1.884450 3.317559
33 1 0 -0.798342 0.692878 4.379755
34 6 0 0.555295 2.059781 1.480720
35 1 0 -0.323303 2.633255 1.169456
36 1 0 1.237703 2.746153 1.993864
37 1 0 1.063955 1.692455 0.582651
38 1 0 -0.902373 -1.844671 3.384412
39 1 0 -2.331977 -0.778081 3.314886
40 1 0 -2.199327 -2.175587 2.218425
41 27 0 0.278736 -1.478065 -0.053391
42 7 0 -3.182757 1.059608 -1.430589
43 7 0 -2.918372 1.394180 1.357144
44 6 0 -4.696799 3.002755 1.334587
45 1 0 -4.586576 3.107659 2.408849
46 6 0 -5.620839 3.726364 0.626959
47 1 0 -6.272256 4.424497 1.142176
48 6 0 -5.738724 3.575411 -0.778777
49 6 0 -4.931108 2.703168 -1.461079
50 1 0 -5.009724 2.569545 -2.534883
51 1 0 -6.479605 4.159535 -1.314661
52 8 0 0.647396 -2.988747 1.277845
53 6 0 1.857217 -3.021833 1.607035
54 6 0 2.355710 -3.952715 2.663869
55 1 0 1.637251 -3.971127 3.485342
56 1 0 2.420232 -4.960965 2.244145
57 1 0 3.339563 -3.662985 3.036415
58 7 0 2.721614 -2.208184 0.996413

59 1 0 3.696638 -2.199009 1.269778
60 6 0 2.285949 -1.394697 -0.126903
61 6 0 3.292758 -0.391785 -0.418383
62 6 0 4.134292 0.454422 -0.618168
63 6 0 1.863378 -2.246853 -1.323540
64 1 0 2.381471 -3.206826 -1.322333
65 6 0 5.103553 1.486837 -0.845906
66 6 0 4.782158 2.824648 -0.571661
67 6 0 6.376182 1.169971 -1.342729
68 6 0 5.722167 3.825130 -0.789758
69 1 0 3.797199 3.069342 -0.186483
70 6 0 7.310335 2.176902 -1.557171
71 1 0 6.623610 0.135315 -1.557147
72 6 0 6.986327 3.503940 -1.281548
73 1 0 5.468279 4.858183 -0.574620
74 1 0 8.293544 1.924769 -1.941644
75 1 0 7.718288 4.287673 -1.449510
76 1 0 1.989251 -1.731314 -2.273646
77 1 0 1.237764 -0.363838 -0.130854
78 1 0 0.771125 -2.588951 -1.300422

25

E(UwB97XD/6-311G++(d, p)) -2877.618265 a. u.

∆G(RwB97XD/6-31G(d, p)) = 0.377429 a. u.

Standard orientation:

Center Atomic Atomic Coordinates (Angstroms)
Number Number Type X Y Z
----
1 6 0 -0.542431 0.608368 0.258312
2 6 0 -0.541208 -0.697993 -0.312984
3 6 0 -2.818490 -0.692946 -0.325483
4 6 0 -2.820083 0.587799 0.291026
5 15 0 1.111631 1.381734 0.477036
6 15 0 1.112885 -1.454702 -0.574026
7 6 0 1.120933 1.735219 2.269475
8 1 0 1.996701 2.337154 2.527359
9 1 0 0.215786 2.260011 2.585636
10 1 0 1.186147 0.783863 2.804206
11 6 0 0.927632 -2.194371 -2.231783
12 6 0 1.003756 3.032984 -0.384512
13 6 0 0.461119 2.788710 -1.799082
14 1 0 1.041003 2.027897 -2.333979
15 1 0 -0.585638 2.469643 -1.781509

16 1 0 0.516711 3.720467 -2.372969
17 6 0 2.450957 3.544649 -0.462296
18 1 0 2.476367 4.513608 -0.973360
19 1 0 2.885590 3.680022 0.534423
20 1 0 3.087877 2.850528 -1.022010
21 6 0 0.128318 4.055208 0.346797
22 1 0 0.535460 4.303974 1.331521
23 1 0 0.090203 4.981340 -0.239144
24 1 0 -0.893430 3.689263 0.476755
25 6 0 1.176757 -2.876888 0.635166
26 6 0 2.575365 -3.496291 0.492279
27 1 0 2.728557 -3.925111 -0.503687
28 1 0 3.364011 -2.756898 0.673156
29 1 0 2.696071 -4.302708 1.224484
30 6 0 0.108062 -3.945540 0.385987
31 1 0 0.196769 -4.726067 1.150905
32 1 0 -0.902306 -3.531285 0.440105
33 1 0 0.231463 -4.423484 -0.590764
34 6 0 1.010658 -2.283076 2.040970
35 1 0 0.033905 -1.804165 2.168566
36 1 0 1.088953 -3.082076 2.786838
37 1 0 1.787089 -1.541390 2.257852
38 1 0 1.795383 -2.817300 -2.464621
39 1 0 0.020149 -2.798617 -2.303824
40 1 0 0.874715 -1.385257 -2.965219
41 27 0 2.578745 0.069005 -0.313853
42 7 0 -1.652823 1.230786 0.575029
43 7 0 -1.649927 -1.331512 -0.612421
44 6 0 -4.049382 -1.323613 -0.630129
45 1 0 -4.025461 -2.300926 -1.100922
46 6 0 -5.229398 -0.695496 -0.324692
47 1 0 -6.174672 -1.176594 -0.553549
48 6 0 -5.231192 0.580245 0.293963
49 6 0 -4.053184 1.213341 0.597443
50 1 0 -4.033134 2.191096 1.067479
51 1 0 -6.178000 1.057650 0.524146

26

E(UwB97XD/6-311G++(d, p)) -3471.612939 a. u.

ΔG(RwB97XD/6-31G(d, p)) = 0.558695 a. u.

Standard orientation:

Center Atomic Atomic Coordinates (Angstroms)
Number Number Type X Y Z

----

1 6 0 1.980305 -0.999240 0.416767

2 6 0 2.523164 -0.171442 -0.619023
3 6 0 4.486770 -1.334235 -0.510972
4 6 0 3.984546 -2.085072 0.583740
5 15 0 0.182039 -0.770141 0.815397
6 15 0 1.462011 1.237693 -1.189916
7 6 0 0.194142 -0.930281 2.635207
8 1 0 -0.832568 -1.023429 2.999879
9 1 0 0.782343 -1.785030 2.977459
10 1 0 0.617470 -0.009495 3.044685
11 6 0 1.448547 0.980380 -3.001729
12 6 0 -0.562945 -2.361590 0.153849
13 6 0 -0.328220 -2.361885 -1.363531
14 1 0 -0.739276 -1.460085 -1.832351
15 1 0 0.736938 -2.424387 -1.611608
16 1 0 -0.825124 -3.229221 -1.813219
17 6 0 -2.070390 -2.299874 0.436864
18 1 0 -2.550997 -3.207774 0.053743
19 1 0 -2.281303 -2.240203 1.510219
20 1 0 -2.535905 -1.439342 -0.048784
21 6 0 0.015586 -3.634634 0.778777
22 1 0 -0.164555 -3.671774 1.857868
23 1 0 -0.476096 -4.507119 0.331557
24 1 0 1.090584 -3.729903 0.609826
25 6 0 2.569736 2.729241 -0.903069
26 6 0 1.686569 3.975986 -1.054727
27 1 0 1.189963 4.007752 -2.031087
28 1 0 0.918247 4.008226 -0.278039
29 1 0 2.307053 4.875585 -0.965252
30 6 0 3.741964 2.812200 -1.887582
31 1 0 4.376057 3.665360 -1.616884
32 1 0 4.356093 1.908354 -1.863955
33 1 0 3.397374 2.971021 -2.914358
34 6 0 3.094631 2.657727 0.538335
35 1 0 3.809391 1.839696 0.672462
36 1 0 3.616117 3.592532 0.776534
37 1 0 2.277232 2.534705 1.255614
38 1 0 1.013829 1.859527 -3.485633
39 1 0 2.447487 0.799639 -3.406340
40 1 0 0.812108 0.118955 -3.223728
41 8 0 -0.000687 2.273171 1.750129
42 6 0 -1.084972 2.671625 2.221770
43 7 0 -2.240160 2.426854 1.594858
44 6 0 -2.252493 1.765651 0.301287
45 6 0 -1.621965 2.500854 -0.809072
46 1 0 -2.057871 2.325218 -1.796543
47 1 0 -3.094748 2.815872 1.972166
48 6 0 -1.128629 3.455215 3.503286
49 1 0 -0.578030 2.907836 4.272327
50 1 0 -0.624891 4.414025 3.351610
51 1 0 -2.146931 3.640599 3.850316

52 1 0 -1.422652 3.560685 -0.633775
53 6 0 -3.437460 1.003426 0.071938
54 6 0 -4.420307 0.325068 -0.163989
55 6 0 -5.532371 -0.530610 -0.441744
56 6 0 -6.191892 -0.464867 -1.680456
57 6 0 -5.971284 -1.465203 0.511581
58 6 0 -7.257909 -1.313428 -1.955892
59 1 0 -5.859604 0.254555 -2.422217
60 6 0 -7.044810 -2.303305 0.231165
61 1 0 -5.462711 -1.528171 1.468623
62 6 0 -7.690879 -2.233005 -1.002043
63 1 0 -7.755710 -1.252545 -2.918803
64 1 0 -7.374866 -3.019170 0.977743
65 1 0 -8.525479 -2.892219 -1.219358
66 27 0 -0.437678 1.128999 -0.118483
67 7 0 2.707298 -1.910388 1.022315
68 7 0 3.732634 -0.362110 -1.093923
69 6 0 5.801015 -1.576242 -0.981714
70 1 0 6.164422 -0.993937 -1.822362
71 6 0 6.579657 -2.521839 -0.365602
72 1 0 7.588267 -2.708197 -0.719868
73 6 0 6.083830 -3.261102 0.738414
74 6 0 4.812640 -3.050923 1.207708
75 1 0 4.411311 -3.610355 2.046583
76 1 0 6.721237 -4.001485 1.210745

28

E(UwB97XD/6-311G++(d, p)) = -3472.774527 a. u.

ΔG(RwB97XD/6-31G(d, p)) = 0.583639 a. u.

Standard orientation:

Center Atomic Atomic Coordinates (Angstroms)
Number Number Type X Y Z
----
1 6 0 1.945658 -1.025817 0.349344
2 6 0 2.560439 -0.112061 -0.506088
3 6 0 4.556716 -1.242412 -0.306363
4 6 0 3.934789 -2.158191 0.599840
5 15 0 0.146023 -0.891645 0.604438

6 15 0 1.577695 1.285784 -1.143058
7 6 0 -0.084410 -1.112204 2.401953
8 1 0 -1.148843 -1.212250 2.630055
9 1 0 0.454013 -1.992011 2.760485
10 1 0 0.305782 -0.230523 2.914470
11 6 0 1.599221 1.047797 -2.957310
12 6 0 -0.582068 -2.434572 -0.181722
13 6 0 -0.052466 -2.527481 -1.618779
14 1 0 -0.273805 -1.622755 -2.195600
15 1 0 1.029199 -2.692155 -1.640715
16 1 0 -0.532753 -3.370812 -2.127978
17 6 0 -2.109352 -2.286140 -0.200050
18 1 0 -2.559953 -3.208956 -0.583544
19 1 0 -2.515066 -2.108961 0.801731
20 1 0 -2.432611 -1.464735 -0.846189
21 6 0 -0.206747 -3.705961 0.591093
22 1 0 -0.668344 -3.723788 1.583102
23 1 0 -0.574837 -4.580541 0.041597
24 1 0 0.875435 -3.797426 0.712147
25 6 0 2.588414 2.837204 -0.826829
26 6 0 1.637585 4.035628 -0.946825
27 1 0 1.109040 4.043177 -1.906907
28 1 0 0.894122 4.028558 -0.146114
29 1 0 2.211538 4.966873 -0.873773
30 6 0 3.734478 2.990870 -1.836394
31 1 0 4.331721 3.869470 -1.563292
32 1 0 4.387218 2.114491 -1.833247
33 1 0 3.362665 3.149659 -2.853282
34 6 0 3.160468 2.775947 0.596082
35 1 0 3.909316 1.984221 0.687964
36 1 0 3.648561 3.730835 0.825049
37 1 0 2.376119 2.606343 1.338274
38 1 0 1.154198 1.915684 -3.451901
39 1 0 2.614585 0.893990 -3.329998
40 1 0 0.999951 0.164701 -3.196138
41 8 0 0.136567 1.919795 1.452816
42 6 0 -0.798426 2.264043 2.232232
43 7 0 -2.045668 2.112969 1.819873
44 6 0 -1.662423 2.668450 -0.533693
45 6 0 -2.240402 1.744785 0.429637
46 1 0 -2.822659 2.417302 2.393647
47 6 0 -0.494625 2.832345 3.575361
48 1 0 0.189970 2.163078 4.101346
49 1 0 0.008139 3.794675 3.444711
50 1 0 -1.399181 2.976034 4.166895
51 27 0 -0.483712 1.137766 -0.210165
52 7 0 2.602294 -2.055825 0.932393
53 7 0 3.861741 -0.196211 -0.870389
54 6 0 5.916893 -1.416880 -0.617991
55 1 0 6.373140 -0.713041 -1.309380

56 6 0 6.657612 -2.452667 -0.059323
57 1 0 7.707340 -2.566865 -0.313725
58 6 0 6.051168 -3.343963 0.830344
59 6 0 4.706649 -3.195238 1.152600
60 1 0 4.217502 -3.881284 1.839395
61 1 0 6.626407 -4.153748 1.269582
62 1 0 -2.142411 2.733963 -1.506947
63 6 0 -3.461225 1.041725 0.182903
64 6 0 -4.474394 0.409620 -0.029798
65 6 0 -5.648431 -0.363762 -0.299228
66 6 0 -6.636020 -0.528806 0.684252
67 6 0 -5.814750 -0.978488 -1.550295
68 6 0 -7.766551 -1.292105 0.415983
69 1 0 -6.509873 -0.055519 1.652754
70 6 0 -6.950285 -1.737320 -1.809484
71 1 0 -5.050966 -0.855973 -2.311731
72 6 0 -7.928188 -1.897094 -0.829233
73 1 0 -8.525516 -1.412934 1.182710
74 1 0 -7.071001 -2.207952 -2.780154
75 1 0 -8.814067 -2.489391 -1.035610
76 1 0 -1.320358 3.628489 -0.152088
77 1 0 -1.178753 0.229623 -1.311247
78 1 0 -0.897572 0.890286 -1.711554

TS14

E(UwB97XD/6-311G++(d, p)) -3472.765982 a. u.

ΔG(RwB97XD/6-31G(d, p)) = 0.580132 a. u.

$\nu = i891.96$

Standard orientation:

Center Atomic Atomic Coordinates (Angstroms)
Number Number Type X Y Z
----
1 6 0 1.825313 -1.046863 0.368074
2 6 0 2.534878 -0.201177 -0.478610
3 6 0 4.437476 -1.455889 -0.175694
4 6 0 3.719971 -2.298440 0.731135
5 15 0 0.020286 -0.798296 0.505199
6 15 0 1.668683 1.250554 -1.157123
7 6 0 -0.364707 -1.056521 2.270432
8 1 0 -1.449115 -1.064266 2.405097
9 1 0 0.058002 -1.990838 2.641522
10 1 0 0.054807 -0.224548 2.839047
11 6 0 1.739122 1.030032 -2.968541
12 6 0 -0.731796 -2.271374 -0.391550
13 6 0 -0.091228 -2.360164 -1.780424
14 1 0 -0.231359 -1.434936 -2.347378

15 1 0 0.981107 -2.568194 -1.720895
16 1 0 -0.564083 -3.172717 -2.342304
17 6 0 -2.237139 -2.023981 -0.538540
18 1 0 -2.696892 -2.885138 -1.036481
19 1 0 -2.727664 -1.899850 0.431273
20 1 0 -2.439581 -1.134739 -1.138642
21 6 0 -0.499054 -3.582964 0.367479
22 1 0 -1.020052 -3.595216 1.328194
23 1 0 -0.894629 -4.411101 -0.231733
24 1 0 0.562620 -3.764797 0.547390
25 6 0 2.748832 2.748095 -0.794373
26 6 0 1.859173 3.992512 -0.923070
27 1 0 1.375462 4.049926 -1.903704
28 1 0 1.084083 4.006657 -0.154833
29 1 0 2.476630 4.889931 -0.803859
30 6 0 3.921212 2.855310 -1.776552
31 1 0 4.550335 3.703216 -1.481207
32 1 0 4.540234 1.955476 -1.768732
33 1 0 3.582261 3.037786 -2.799511
34 6 0 3.285505 2.638594 0.637481
35 1 0 4.002600 1.818739 0.730492
36 1 0 3.802136 3.570072 0.895517
37 1 0 2.479953 2.482321 1.358099
38 1 0 1.363375 1.931204 -3.459131
39 1 0 2.757590 0.828232 -3.303358
40 1 0 1.095099 0.190621 -3.236816
41 8 0 0.213519 2.059930 1.420885
42 6 0 -0.723083 2.419573 2.169928
43 7 0 -1.974722 2.286111 1.743964
44 6 0 -1.673412 2.779474 -0.653466
45 6 0 -2.204411 1.897352 0.372050
46 1 0 -2.746726 2.596494 2.319592
47 6 0 -0.456117 2.989872 3.522694
48 1 0 0.188204 2.304337 4.076700
49 1 0 0.076681 3.936897 3.407509
50 1 0 -1.377717 3.161242 4.078485
51 27 0 -0.415229 1.258644 -0.288479
52 7 0 2.386056 -2.100094 0.995106
53 7 0 3.835932 -0.385225 -0.789942
54 6 0 5.792028 -1.735106 -0.430829
55 1 0 6.326753 -1.092663 -1.124597
56 6 0 6.436703 -2.798252 0.188705
57 1 0 7.483309 -2.992950 -0.021959
58 6 0 5.738867 -3.612533 1.084996
59 6 0 4.398398 -3.362462 1.349905
60 1 0 3.843127 -3.993157 2.038143
61 1 0 6.240760 -4.442272 1.572046
62 1 0 -2.263407 2.881026 -1.559696
63 6 0 -3.407352 1.159073 0.171717
64 6 0 -4.410494 0.503198 0.012895

65 6 0 -5.527139 -0.361535 -0.210410
66 6 0 -6.493727 -0.558761 0.785956
67 6 0 -5.654747 -1.039098 -1.431591
68 6 0 -7.563439 -1.416127 0.560932
69 1 0 -6.399995 -0.038019 1.732684
70 6 0 -6.729075 -1.892540 -1.648622
71 1 0 -4.906822 -0.894562 -2.203525
72 6 0 -7.686083 -2.083788 -0.655133
73 1 0 -8.306029 -1.561045 1.338300
74 1 0 -6.818418 -2.411610 -2.596816
75 1 0 -8.523674 -2.750792 -0.828011
76 1 0 -1.214896 3.710274 -0.330784
77 1 0 -0.905173 0.635351 -1.567479
78 1 0 -0.908113 1.648315 -1.643671

30

E(UwB97XD/6-311G++(d, p)) = -3472.797984 a. u.

∆G(RwB97XD/6-31G(d, p)) = 0.585465 a. u.

Standard orientation:

Center Atomic Atomic Coordinates (Angstroms)
Number Number Type X Y Z
----
1 6 0 1.925360 -1.021159 0.303139
2 6 0 2.585298 -0.130742 -0.538750
3 6 0 4.531537 -1.332130 -0.317202
4 6 0 3.862078 -2.225913 0.577813
5 15 0 0.124227 -0.828538 0.497167
6 15 0 1.638057 1.296897 -1.135778
7 6 0 -0.176641 -1.110111 2.272640
8 1 0 -1.249160 -1.169134 2.473969
9 1 0 0.311986 -2.029197 2.602068
10 1 0 0.247457 -0.272414 2.829837
11 6 0 1.653528 1.156159 -2.955871
12 6 0 -0.608701 -2.303973 -0.397956
13 6 0 -0.004811 -2.362867 -1.806385
14 1 0 -0.184802 -1.436528 -2.360497
15 1 0 1.073042 -2.549426 -1.780555
16 1 0 -0.475760 -3.182132 -2.361036
17 6 0 -2.126103 -2.127415 -0.494653
18 1 0 -2.563608 -3.019040 -0.958527
19 1 0 -2.585279 -2.005717 0.491480
20 1 0 -2.392143 -1.260380 -1.102963
21 6 0 -0.300291 -3.610527 0.349009

22 1 0 -0.817205 -3.655854 1.312420
23 1 0 -0.657879 -4.451944 -0.255911
24 1 0 0.771131 -3.736069 0.523208
25 6 0 2.661770 2.821785 -0.739257
26 6 0 1.728301 4.039131 -0.811149
27 1 0 1.218619 4.106916 -1.779271
28 1 0 0.971287 4.007467 -0.021746
29 1 0 2.313255 4.956758 -0.680234
30 6 0 3.816934 3.002561 -1.733503
31 1 0 4.427533 3.857242 -1.417919
32 1 0 4.455365 2.115855 -1.764484
33 1 0 3.456238 3.211371 -2.745215
34 6 0 3.222512 2.683298 0.682646
35 1 0 3.968582 1.885465 0.735671
36 1 0 3.711978 3.622618 0.966307
37 1 0 2.432076 2.476858 1.408626
38 1 0 1.219034 2.054887 -3.402457
39 1 0 2.667806 1.013660 -3.336039
40 1 0 1.043111 0.296272 -3.240997
41 8 0 0.173030 2.004644 1.545887
42 6 0 -0.802199 2.236880 2.311576
43 7 0 -2.024460 1.981694 1.867941
44 6 0 -2.161687 1.594538 0.472546
45 6 0 -1.833561 2.730370 -0.479563
46 1 0 -2.450499 2.729015 -1.375773
47 1 0 -2.836124 2.195667 2.434698
48 6 0 -0.589446 2.789450 3.680735
49 1 0 0.075324 2.122008 4.234736
50 1 0 -0.094798 3.760395 3.595024
51 1 0 -1.527345 2.907590 4.224313
52 1 0 -1.842149 3.707471 0.007220
53 6 0 -3.397693 0.909157 0.232601
54 6 0 -4.436906 0.317290 0.035210
55 6 0 -5.634100 -0.420103 -0.232927
56 6 0 -6.325741 -0.227846 -1.439025
57 6 0 -6.121353 -1.351480 0.697324
58 6 0 -7.479966 -0.954944 -1.705716
59 1 0 -5.951408 0.491197 -2.160642
60 6 0 -7.279311 -2.070770 0.423322
61 1 0 -5.588707 -1.504690 1.630573
62 6 0 -7.960866 -1.876197 -0.776804
63 1 0 -8.005687 -0.800912 -2.642851
64 1 0 -7.648634 -2.788406 1.149278
65 1 0 -8.864347 -2.439864 -0.987340
66 27 0 -0.388099 1.174854 -0.184972
67 7 0 2.527819 -2.077490 0.891466
68 7 0 3.886083 -0.257214 -0.889359
69 6 0 5.888772 -1.557447 -0.607934
70 1 0 6.382709 -0.870082 -1.289839
71 6 0 6.581208 -2.620780 -0.039417

72 1 0 7.629881 -2.773097 -0.277500
73 6 0 5.928218 -3.490037 0.838989
74 6 0 4.585587 -3.291469 1.140378
75 1 0 4.059675 -3.959925 1.817067
76 1 0 6.465504 -4.321502 1.285547
77 1 0 -0.861187 0.535607 -1.412951
78 1 0 -0.761170 2.691221 -0.902195

TS18

E(UwB97XD/6-311G++(d, p)) -3472.752066 a. u.

ΔG(RwB97XD/6-31G(d, p)) = 0.578953 a. u.

$\nu = i726.34$

Standard orientation:

Center Atomic Atomic Coordinates (Angstroms)
Number Number Type X Y Z
----
1 6 0 -2.219177 0.475669 0.682180
2 6 0 -2.277672 0.407033 -0.703979
3 6 0 -4.027443 1.890064 -0.728528
4 6 0 -3.983447 1.943475 0.701893
5 15 0 -0.899550 -0.495830 1.469750
6 15 0 -1.142144 -0.771875 -1.490557
7 6 0 -1.767693 -1.630523 2.606351
8 1 0 -1.055558 -2.103824 3.285966
9 1 0 -2.523923 -1.090536 3.181354
10 1 0 -2.259903 -2.407802 2.016690
11 6 0 -0.425418 0.182514 -2.878349
12 6 0 0.022810 0.697470 2.587746
13 6 0 0.409963 1.927582 1.756773
14 1 0 0.988914 1.654174 0.867263
15 1 0 -0.472840 2.487561 1.433622
16 1 0 1.029398 2.596138 2.365745
17 6 0 1.291007 0.005945 3.106784
18 1 0 1.778390 0.655966 3.842571
19 1 0 1.065246 -0.944638 3.602922
20 1 0 2.010183 -0.185399 2.307188
21 6 0 -0.834213 1.128697 3.787243
22 1 0 -1.041978 0.288716 4.457654
23 1 0 -0.284143 1.881678 4.364597
24 1 0 -1.784686 1.558679 3.463553
25 6 0 -2.246009 -2.057263 -2.307601
26 6 0 -1.369737 -3.235570 -2.759771
27 1 0 -0.516994 -2.909036 -3.365643
28 1 0 -0.987116 -3.806111 -1.906826

29 1 0 -1.967414 -3.918745 -3.374384
30 6 0 -2.995869 -1.489881 -3.520514
31 1 0 -3.713533 -2.236789 -3.881159
32 1 0 -3.545024 -0.580549 -3.263919
33 1 0 -2.313157 -1.263205 -4.345696
34 6 0 -3.251350 -2.545024 -1.258063
35 1 0 -3.977008 -1.766110 -1.005776
36 1 0 -3.801684 -3.406225 -1.654456
37 1 0 -2.751465 -2.860851 -0.334867
38 1 0 0.122499 -0.474341 -3.559149
39 1 0 -1.202858 0.712207 -3.434154
40 1 0 0.275120 0.911767 -2.462408
41 8 0 0.725435 -2.992099 1.245558
42 6 0 1.941112 -3.031195 1.556469
43 7 0 2.791758 -2.201794 0.950904
44 6 0 2.326122 -1.361011 -0.137002
45 6 0 1.897214 -2.184070 -1.351539
46 1 0 2.004012 -1.638478 -2.287461
47 1 0 3.770257 -2.189972 1.210319
48 6 0 2.453309 -3.983254 2.587726
49 1 0 1.840918 -3.895348 3.488001
50 1 0 2.350659 -5.004002 2.208849
51 1 0 3.499679 -3.798574 2.834554
52 1 0 2.436500 -3.132410 -1.390423
53 6 0 3.298411 -0.322738 -0.404284
54 6 0 4.120361 0.549855 -0.575756
55 6 0 5.077655 1.602394 -0.759592
56 6 0 6.231927 1.388787 -1.527179
57 6 0 4.869671 2.855009 -0.162018
58 6 0 7.160103 2.410540 -1.691380
59 1 0 6.392887 0.420366 -1.989820
60 6 0 5.803309 3.871080 -0.330465
61 1 0 3.974661 3.021397 0.429322
62 6 0 6.949137 3.651668 -1.093307
63 1 0 8.052405 2.236810 -2.284402
64 1 0 5.636967 4.837290 0.135199
65 1 0 7.676754 4.446808 -1.222504
66 27 0 0.305901 -1.479404 -0.043633
67 7 0 -3.062609 1.222514 1.431055
68 7 0 -3.160540 1.101605 -1.455260
69 6 0 -4.991041 2.658721 -1.405233
70 1 0 -5.012696 2.602991 -2.490588
71 6 0 -5.891932 3.460676 -0.713288
72 1 0 -6.627000 4.044474 -1.259665
73 6 0 -5.849566 3.512315 0.682703
74 6 0 -4.906543 2.762389 1.376597
75 1 0 -4.856886 2.794258 2.462002
76 1 0 -6.550240 4.137803 1.228128
77 1 0 1.221554 -0.337428 -0.055623
78 1 0 0.811401 -2.546835 -1.325434

TS15

E(UwB97XD/6-311G++(d, p)) -3472.751368 a. u.

ΔG(RwB97XD/6-31G(d, p)) = 0.581568 a. u.

ν = *i*829.01

Standard orientation:

Center Atomic Atomic Coordinates (Angstroms)
Number Number Type X Y Z
----
1 6 0 2.188397 -0.923697 0.379590
2 6 0 2.568088 -0.077373 -0.661449
3 6 0 4.599802 -1.148660 -0.818560
4 6 0 4.226452 -1.990574 0.276202
5 15 0 0.503785 -0.760679 1.055033
6 15 0 1.436752 1.278852 -1.111722
7 6 0 0.752396 -0.536561 2.848817
8 1 0 -0.199980 -0.640956 3.376084
9 1 0 1.468335 -1.264539 3.237029
10 1 0 1.135738 0.471751 3.017819
11 6 0 1.296038 1.180736 -2.929803
12 6 0 -0.299177 -2.444150 0.859503
13 6 0 -0.088837 -2.909139 -0.586783
14 1 0 -0.451036 -2.172727 -1.312419
15 1 0 0.967935 -3.103650 -0.792817
16 1 0 -0.641153 -3.841174 -0.752142
17 6 0 -1.800449 -2.298284 1.150148
18 1 0 -2.256265 -3.293115 1.212287
19 1 0 -1.985981 -1.788296 2.102080
20 1 0 -2.321614 -1.749749 0.360086
21 6 0 0.299825 -3.475378 1.827300
22 1 0 0.071436 -3.231660 2.869782
23 1 0 -0.138480 -4.458237 1.616804
24 1 0 1.384499 -3.543222 1.715372
25 6 0 2.389383 2.859010 -0.764844
26 6 0 1.403018 4.033191 -0.819327
27 1 0 0.870503 4.073973 -1.776019
28 1 0 0.664419 3.970423 -0.016382
29 1 0 1.953534 4.974469 -0.704955
30 6 0 3.510070 3.086508 -1.788008
31 1 0 4.085444 3.973534 -1.495829
32 1 0 4.188152 2.230645 -1.833677
33 1 0 3.110572 3.269089 -2.790442
34 6 0 2.985904 2.749130 0.644994
35 1 0 3.756189 1.973566 0.693156
36 1 0 3.454130 3.703310 0.914055
37 1 0 2.216984 2.527418 1.391015

38 1 0 0.784149 2.068092 -3.311467
39 1 0 2.278074 1.092597 -3.400532
40 1 0 0.701744 0.298862 -3.182820
41 8 0 -0.169342 2.111344 1.555766
42 6 0 -1.261607 2.436486 2.090154
43 7 0 -2.397425 2.048609 1.516418
44 6 0 -1.651508 2.289270 -0.823148
45 6 0 -2.340080 1.473575 0.181509
46 1 0 -3.291246 2.318524 1.909155
47 6 0 -1.286112 3.236819 3.348768
48 1 0 -0.669628 2.734493 4.097925
49 1 0 -0.841774 4.215687 3.149588
50 1 0 -2.297280 3.370993 3.734251
51 27 0 -0.462700 0.964604 -0.018150
52 7 0 2.997200 -1.883820 0.886900
53 7 0 3.759509 -0.166220 -1.295591
54 6 0 5.862509 -1.326932 -1.410570
55 1 0 6.130293 -0.680383 -2.242392
56 6 0 6.747022 -2.294181 -0.945394
57 1 0 7.716960 -2.413276 -1.419401
58 6 0 6.386219 -3.109978 0.130754
59 6 0 5.141481 -2.956769 0.731216
60 1 0 4.842914 -3.585943 1.565821
61 1 0 7.076041 -3.864218 0.498185
62 1 0 -1.959429 2.149822 -1.855962
63 6 0 -3.550143 0.754854 -0.137788
64 6 0 -4.557120 0.135238 -0.398663
65 6 0 -5.741820 -0.608239 -0.708202
66 6 0 -6.743664 -0.772382 0.260119
67 6 0 -5.903730 -1.186735 -1.975897
68 6 0 -7.887567 -1.502709 -0.040332
69 1 0 -6.618416 -0.324736 1.240749
70 6 0 -7.051012 -1.916343 -2.265449
71 1 0 -5.128318 -1.061945 -2.724900
72 6 0 -8.044201 -2.076401 -1.300808
73 1 0 -8.658997 -1.624211 0.713533
74 1 0 -7.169408 -2.363017 -3.247517
75 1 0 -8.939233 -2.645641 -1.531215
76 1 0 -1.465940 3.325959 -0.555451
77 1 0 -1.596400 0.111510 -0.350044
78 1 0 -0.900326 0.016337 -1.102508

31

E(UwB97XD/6-311G++(d, p)) = -3472.776592 a. u.

ΔG(RwB97XD/6-31G(d, p)) = 0.585192 a. u.

Standard orientation:

Center Number	Atomic Number	Atomic Type	Coordinates (Angstroms) X	Y	Z
1	6	0	2.375684	-0.871938	0.376438
2	6	0	2.615863	-0.046782	-0.719215
3	6	0	4.691509	-0.992972	-1.003443
4	6	0	4.459448	-1.814697	0.145219
5	15	0	0.754882	-0.734839	1.188132
6	15	0	1.370746	1.227591	-1.103564
7	6	0	1.145959	-0.297082	2.917189
8	1	0	0.241097	-0.340311	3.529724
9	1	0	1.903914	-0.963725	3.334863
10	1	0	1.523203	0.728383	2.930055
11	6	0	1.174559	1.159114	-2.912793
12	6	0	0.034116	-2.464409	1.241344
13	6	0	0.219228	-3.110147	-0.137867
14	1	0	-0.195117	-2.487638	-0.937725
15	1	0	1.276394	-3.291508	-0.352883
16	1	0	-0.301565	-4.074238	-0.158158
17	6	0	-1.463698	-2.341628	1.558372
18	1	0	-1.890696	-3.340843	1.702556
19	1	0	-1.642974	-1.768784	2.475347
20	1	0	-2.010726	-1.864035	0.738941
21	6	0	0.710642	-3.332124	2.311657
22	1	0	0.521739	-2.952086	3.320444
23	1	0	0.301558	-4.348176	2.259145
24	1	0	1.790869	-3.384412	2.155070
25	6	0	2.198792	2.869689	-0.741932
26	6	0	1.140868	3.977583	-0.841540
27	1	0	0.652365	3.986173	-1.821641

28	1	0	0.373009	3.867500	-0.072147
29	1	0	1.625725	4.951177	-0.703674
30	6	0	3.331995	3.158874	-1.736019
31	1	0	3.834807	4.087192	-1.439518
32	1	0	4.069330	2.352702	-1.751897
33	1	0	2.950872	3.300745	-2.752132
34	6	0	2.758367	2.809422	0.685642
35	1	0	3.550377	2.059644	0.776371
36	1	0	3.189019	3.783697	0.944429
37	1	0	1.974464	2.582712	1.414118
38	1	0	0.615578	2.029298	-3.265501
39	1	0	2.150762	1.127373	-3.401869
40	1	0	0.619046	0.254469	-3.169765
41	8	0	-0.397282	2.141994	1.522678
42	6	0	-1.521361	2.541800	1.908798
43	7	0	-2.625091	2.075589	1.322950
44	6	0	-1.699550	1.798786	-0.944277
45	6	0	-2.470968	1.172057	0.186404
46	1	0	-3.544958	2.390960	1.599636
47	6	0	-1.645635	3.528128	3.022717
48	1	0	-1.142004	3.126625	3.905220
49	1	0	-1.133968	4.449398	2.732625
50	1	0	-2.685842	3.749597	3.263143
51	27	0	-0.395960	0.773685	0.031671
52	7	0	3.276609	-1.763261	0.849839
53	7	0	3.754675	-0.085195	-1.448172
54	6	0	5.915993	-1.111804	-1.684389
55	1	0	6.075216	-0.481907	-2.555835
56	6	0	6.896326	-2.000696	-1.256620
57	1	0	7.835312	-2.073439	-1.797595

58	6	0	6.672734	-2.796588	-0.129747
59	6	0	5.468602	-2.701858	0.558861
60	1	0	5.275925	-3.315163	1.435355
61	1	0	7.436168	-3.491179	0.208393
62	1	0	-1.994109	1.455583	-1.933731
63	6	0	-3.715454	0.498236	-0.152486
64	6	0	-4.747017	-0.073939	-0.414119
65	6	0	-5.965725	-0.753525	-0.743544
66	6	0	-7.144551	-0.469473	-0.039355
67	6	0	-5.983254	-1.706691	-1.771952
68	6	0	-8.322152	-1.133451	-0.363042
69	1	0	-7.130092	0.269774	0.754878
70	6	0	-7.165650	-2.366683	-2.085708
71	1	0	-5.069444	-1.924942	-2.315018
72	6	0	-8.335746	-2.081610	-1.384310
73	1	0	-9.232623	-0.909664	0.183731
74	1	0	-7.172899	-3.106098	-2.880072
75	1	0	-9.257433	-2.598654	-1.631941
76	1	0	-1.677441	2.885878	-0.875329
77	1	0	-1.856670	0.266741	0.653746
78	1	0	-0.486308	-0.269023	-0.980451

TS19

E(UwB97XD/6-311G++(d, p)) - 3472.146894 a. u.

ΔG(RwB97XD/6-31G(d, p)) = 0.562897 a. u.

ν = *i*763.69

Standard orientation:

Center Atomic Atomic Coordinates (Angstroms)
Number Number Type X Y Z

----
1 6 0 2.243499 -0.862508 0.392052
2 6 0 2.597225 -0.071217 -0.750044
3 6 0 4.562934 -1.220797 -0.933968
4 6 0 4.244153 -1.957816 0.236310
5 15 0 0.560278 -0.588048 1.121548
6 15 0 1.436548 1.293295 -1.214018
7 6 0 0.977866 -0.144713 2.846236
8 1 0 0.065101 -0.155780 3.449013
9 1 0 1.707894 -0.831872 3.280681
10 1 0 1.376038 0.870933 2.854750
11 6 0 1.449365 1.213744 -3.038348
12 6 0 -0.174374 -2.313674 1.233452
13 6 0 -0.017791 -3.003573 -0.128654
14 1 0 -0.414727 -2.391281 -0.944267
15 1 0 1.030212 -3.232088 -0.346521
16 1 0 -0.569364 -3.950876 -0.120039
17 6 0 -1.665724 -2.125127 1.554554
18 1 0 -2.127200 -3.103510 1.731589
19 1 0 -1.812883 -1.518205 2.455195
20 1 0 -2.201516 -1.648515 0.729160
21 6 0 0.469082 -3.175950 2.326938
22 1 0 0.291378 -2.763447 3.324805
23 1 0 0.022453 -4.177151 2.299692
24 1 0 1.546965 -3.276903 2.180326
25 6 0 2.426703 2.828367 -0.774690
26 6 0 1.470020 4.023732 -0.887355
27 1 0 1.030160 4.102844 -1.887818
28 1 0 0.662060 3.944881 -0.155217
29 1 0 2.022420 4.950899 -0.693363
30 6 0 3.633314 3.041593 -1.694585
31 1 0 4.197409 3.915597 -1.347385
32 1 0 4.308238 2.180867 -1.692958
33 1 0 3.327670 3.236208 -2.727290
34 6 0 2.889542 2.690025 0.682364
35 1 0 3.616019 1.880255 0.807870
36 1 0 3.377093 3.620933 0.995235
37 1 0 2.040786 2.514170 1.349686
38 1 0 0.944484 2.097460 -3.437969
39 1 0 2.460892 1.153481 -3.446254
40 1 0 0.886487 0.328198 -3.344992
41 8 0 -0.312159 2.425300 1.688097
42 6 0 -1.454462 2.413840 2.176557
43 7 0 -2.494918 1.900660 1.505596
44 6 0 -1.728421 2.244466 -0.853786
45 6 0 -2.359435 1.357134 0.154249
46 1 0 -3.414526 1.937630 1.923430
47 6 0 -1.724951 2.980377 3.544401
48 1 0 -1.019856 2.537097 4.252242
49 1 0 -1.543797 4.058793 3.525866

50 1 0 -2.745340 2.797792 3.886958
51 27 0 -0.435231 0.927347 -0.188597
52 7 0 3.063035 -1.762941 0.883994
53 7 0 3.719027 -0.262075 -1.405474
54 6 0 5.785069 -1.469695 -1.605966
55 1 0 6.008320 -0.898545 -2.501378
56 6 0 6.652336 -2.410599 -1.113138
57 1 0 7.591616 -2.601570 -1.621890
58 6 0 6.338751 -3.139537 0.062441
59 6 0 5.159590 -2.921400 0.727358
60 1 0 4.898497 -3.471276 1.625945
61 1 0 7.043038 -3.877011 0.433415
62 1 0 -2.138008 2.170907 -1.861298
63 6 0 -3.575802 0.664263 -0.195031
64 6 0 -4.573058 0.038153 -0.488221
65 6 0 -5.755071 -0.697539 -0.823969
66 6 0 -6.768568 -0.879929 0.130199
67 6 0 -5.911208 -1.252212 -2.103884
68 6 0 -7.911673 -1.601741 -0.193631
69 1 0 -6.651619 -0.452194 1.120854
70 6 0 -7.057934 -1.973058 -2.417842
71 1 0 -5.129628 -1.115810 -2.844586
72 6 0 -8.060547 -2.150763 -1.465985
73 1 0 -8.689147 -1.735871 0.552090
74 1 0 -7.168232 -2.399821 -3.409900
75 1 0 -8.954647 -2.713708 -1.714916
76 1 0 -1.587169 3.277656 -0.540153
77 1 0 -1.724601 0.119473 -0.462775
78 1 0 -1.239439 -0.272707 -1.180322

27

E(UwB97XD/6-311G++(d, p)) = -3471.614362 a. u.

ΔG(RwB97XD/6-31G(d, p)) = 0.561692 a. u.

Standard orientation:

Center Atomic Atomic Coordinates (Angstroms)
Number Number Type X Y Z
----
1 6 0 2.580716 -0.174213 -0.276888
2 6 0 1.828085 0.996165 0.068675
3 6 0 3.748853 2.237001 0.004936
4 6 0 4.500811 1.067948 -0.277182
5 15 0 1.629864 -1.748935 -0.529504
6 15 0 0.015637 0.753753 0.391345
7 6 0 2.692308 -2.932775 0.372993
8 1 0 2.382295 -3.956150 0.145889

9 1 0 3.749646 -2.807265 0.126775
10 1 0 2.551735 -2.761658 1.443392
11 6 0 -0.712140 1.766850 -0.952505
12 6 0 1.888424 -2.110550 -2.352604
13 6 0 1.226722 -0.962537 -3.127160
14 1 0 0.167184 -0.861273 -2.864960
15 1 0 1.720457 -0.002996 -2.937599
16 1 0 1.287416 -1.160343 -4.203515
17 6 0 1.157704 -3.425135 -2.663080
18 1 0 1.279499 -3.661679 -3.726558
19 1 0 1.565835 -4.263092 -2.088130
20 1 0 0.087435 -3.353510 -2.452451
21 6 0 3.358714 -2.247323 -2.760825
22 1 0 3.843567 -3.082835 -2.245782
23 1 0 3.415337 -2.445528 -3.837997
24 1 0 3.931085 -1.340945 -2.551614
25 6 0 -0.324062 1.720286 1.961851
26 6 0 -1.784906 1.429146 2.338539
27 1 0 -2.478185 1.752878 1.554521
28 1 0 -1.957012 0.365984 2.522860
29 1 0 -2.038313 1.978164 3.253285
30 6 0 -0.143408 3.236449 1.823063
31 1 0 -0.330614 3.709605 2.794945
32 1 0 0.867235 3.495809 1.502020
33 1 0 -0.854247 3.662590 1.107728
34 6 0 0.610905 1.166250 3.045153
35 1 0 1.657743 1.414469 2.841096
36 1 0 0.349823 1.606150 4.014905
37 1 0 0.521356 0.078072 3.125952
38 1 0 -1.784605 1.889841 -0.775758
39 1 0 -0.242559 2.750846 -1.029905
40 1 0 -0.582034 1.238098 -1.900817
41 8 0 -0.202814 -2.035894 2.313786
42 6 0 -1.350665 -2.228205 2.763357
43 7 0 -2.427392 -2.106138 1.977924
44 6 0 -1.624770 -2.858337 -0.243574
45 6 0 -2.269370 -1.815300 0.566025
46 1 0 -3.343047 -2.304459 2.359585
47 6 0 -1.570071 -2.583835 4.206009
48 1 0 -2.623207 -2.745760 4.442760
49 1 0 -1.004671 -3.487802 4.446123
50 1 0 -1.182749 -1.772695 4.828892
51 27 0 -0.398081 -1.403160 0.186713
52 7 0 3.885542 -0.137148 -0.422861
53 7 0 2.400553 2.173121 0.179146
54 6 0 4.410961 3.484109 0.121439
55 1 0 3.817695 4.367108 0.336705
56 6 0 5.772024 3.547469 -0.033010
57 1 0 6.284039 4.499907 0.057304
58 6 0 6.524876 2.377362 -0.308356

59 6 0 5.906884 1.159113 -0.427322
60 1 0 6.462689 0.251806 -0.641197
61 1 0 7.600643 2.454776 -0.427676
62 1 0 -1.944759 -2.912730 -1.287242
63 6 0 -3.282147 -0.965062 0.031912
64 6 0 -4.096666 -0.191755 -0.439567
65 6 0 -5.030490 0.747418 -0.975635
66 6 0 -5.186414 0.893070 -2.364860
67 6 0 -5.800519 1.553514 -0.119252
68 6 0 -6.087651 1.819527 -2.877065
69 1 0 -4.594732 0.276516 -3.034375
70 6 0 -6.700558 2.475076 -0.641499
71 1 0 -5.684179 1.448919 0.954967
72 6 0 -6.848934 2.613394 -2.020760
73 1 0 -6.196668 1.922191 -3.952462
74 1 0 -7.289211 3.089897 0.032613
75 1 0 -7.552472 3.334706 -2.424434
76 1 0 -1.553472 -3.846980 0.214686

29

E(UwB97XD/6-311G++(d, p)) = -3472.773799 a. u.

ΔG(RwB97XD/6-31G(d, p)) = 0.580551 a. u.

Standard orientation:

Center Atomic Atomic Coordinates (Angstroms)
Number Number Type X Y Z
----
1 6 0 2.589130 -0.161582 -0.271186
2 6 0 1.832760 1.006500 0.074282
3 6 0 3.748385 2.254582 0.008256
4 6 0 4.504205 1.087398 -0.272454
5 15 0 1.646581 -1.743775 -0.515330
6 15 0 0.021423 0.751617 0.394637
7 6 0 2.715244 -2.910660 0.401380
8 1 0 2.420096 -3.939138 0.175963
9 1 0 3.772335 -2.772468 0.160763
10 1 0 2.565751 -2.736572 1.470032
11 6 0 -0.699004 1.743123 -0.968346
12 6 0 1.905186 -2.118714 -2.334211
13 6 0 1.227799 -0.979308 -3.108493
14 1 0 0.169427 -0.882138 -2.839795
15 1 0 1.717048 -0.015732 -2.925837
16 1 0 1.282451 -1.180018 -4.184669
17 6 0 1.186047 -3.443131 -2.630589

18 1 0 1.291120 -3.678827 -3.696006
19 1 0 1.617325 -4.274599 -2.063096
20 1 0 0.119870 -3.385199 -2.396575
21 6 0 3.373013 -2.242312 -2.754199
22 1 0 3.874401 -3.063510 -2.232011
23 1 0 3.421659 -2.456168 -3.828578
24 1 0 3.935981 -1.325610 -2.564384
25 6 0 -0.329145 1.728736 1.953095
26 6 0 -1.785589 1.423902 2.339529
27 1 0 -2.484347 1.674485 1.533519
28 1 0 -1.930277 0.371476 2.594668
29 1 0 -2.056890 2.026946 3.213919
30 6 0 -0.169805 3.245004 1.795845
31 1 0 -0.348137 3.727105 2.764770
32 1 0 0.831638 3.513152 1.452966
33 1 0 -0.900036 3.651024 1.088309
34 6 0 0.616442 1.197099 3.039894
35 1 0 1.658336 1.467197 2.838582
36 1 0 0.343161 1.628415 4.010121
37 1 0 0.551015 0.106528 3.120935
38 1 0 -1.779758 1.832486 -0.821993
39 1 0 -0.258162 2.741281 -1.029735
40 1 0 -0.523084 1.217610 -1.911681
41 8 0 -0.180121 -2.067533 2.300643
42 6 0 -1.327269 -2.256809 2.753787
43 7 0 -2.407220 -2.108078 1.977122
44 6 0 -2.256080 -1.787926 0.569232
45 6 0 -1.631674 -2.823790 -0.266257
46 1 0 -1.948463 -2.839307 -1.312349
47 1 0 -3.322167 -2.309911 2.358627
48 6 0 -1.536605 -2.632913 4.193413
49 1 0 -0.906113 -3.490292 4.439627
50 1 0 -1.219180 -1.792952 4.819107
51 1 0 -2.576665 -2.872974 4.422581
52 1 0 -1.587793 -3.827985 0.161727
53 6 0 -3.274799 -0.928815 0.060075
54 6 0 -4.100021 -0.159464 -0.398656
55 6 0 -5.048274 0.762321 -0.939804
56 6 0 -5.203576 0.894093 -2.330573
57 6 0 -5.837107 1.558062 -0.091243
58 6 0 -6.124647 1.795256 -2.852153
59 1 0 -4.596581 0.285019 -2.993116
60 6 0 -6.756802 2.454697 -0.622762
61 1 0 -5.719447 1.466226 0.983925
62 6 0 -6.906508 2.577350 -2.003432
63 1 0 -6.235430 1.884732 -3.928456
64 1 0 -7.360871 3.061765 0.044700
65 1 0 -7.626958 3.277943 -2.413708
66 27 0 -0.383326 -1.400149 0.193604
67 7 0 3.894020 -0.119581 -0.418211

68 7 0 2.400236 2.186319 0.181748
69 6 0 4.407198 3.503527 0.125250
70 1 0 3.811007 4.384954 0.339444
71 6 0 5.768156 3.570414 -0.028448
72 1 0 6.278162 4.523959 0.061330
73 6 0 6.524798 2.402194 -0.302517
74 6 0 5.910326 1.182412 -0.421317
75 1 0 6.468075 0.275833 -0.633783
76 1 0 7.600330 2.483655 -0.420957
77 1 0 -0.457240 -0.932562 -1.187518
78 1 0 -0.260835 0.042736 -0.982411

TS16

E(UwB97XD/6-311G++(d, p)) -3472.758687 a. u.

ΔG(RwB97XD/6-31G(d, p)) = 0.579266 a. u.

ν = *i*797.14

Standard orientation:

Center Atomic Atomic Coordinates (Angstroms)
Number Number Type X Y Z
----
1 6 0 -2.641708 0.583662 -0.044383
2 6 0 -2.322546 -0.687580 -0.515636
3 6 0 -4.549602 -1.160643 -0.823954
4 6 0 -4.881213 0.130065 -0.303151
5 15 0 -1.255620 1.714658 0.313689
6 15 0 -0.564509 -1.147228 -0.564513
7 6 0 -1.736246 2.520904 1.879914
8 1 0 -1.138454 3.420597 2.043967
9 1 0 -2.796598 2.782795 1.864130
10 1 0 -1.552656 1.820431 2.697967
11 6 0 -0.186179 -1.336122 -2.340412
12 6 0 -1.353868 3.059847 -0.988960
13 6 0 -1.333548 2.394778 -2.369930
14 1 0 -0.413385 1.820303 -2.517248
15 1 0 -2.188598 1.728209 -2.519413
16 1 0 -1.368924 3.169421 -3.144121
17 6 0 -0.128199 3.972902 -0.854015
18 1 0 -0.219358 4.800040 -1.567488
19 1 0 -0.042143 4.405695 0.148363
20 1 0 0.797006 3.435971 -1.079677
21 6 0 -2.630345 3.897644 -0.831269
22 1 0 -2.635111 4.451614 0.112728
23 1 0 -2.677228 4.631418 -1.644466
24 1 0 -3.528873 3.276712 -0.867220
25 6 0 -0.405298 -2.876931 0.147935
26 6 0 1.078365 -3.107894 0.468586

27 1 0 1.709564 -2.951201 -0.413352
28 1 0 1.434596 -2.444405 1.261620
29 1 0 1.221411 -4.141936 0.802527
30 6 0 -0.875716 -3.939180 -0.857829
31 1 0 -0.838919 -4.921903 -0.372924
32 1 0 -1.902352 -3.751438 -1.180895
33 1 0 -0.230678 -3.983540 -1.740324
34 6 0 -1.251716 -2.981567 1.423631
35 1 0 -2.318562 -2.905459 1.194524
36 1 0 -1.075430 -3.957140 1.891950
37 1 0 -0.997060 -2.202259 2.143845
38 1 0 0.787201 -1.817723 -2.466482
39 1 0 -0.956862 -1.927438 -2.839556
40 1 0 -0.146241 -0.347207 -2.800582
41 8 0 0.057036 -0.209088 2.234668
42 6 0 1.031419 -0.377996 3.011260
43 7 0 2.243755 -0.046783 2.582834
44 6 0 1.663330 1.929670 1.256736
45 6 0 2.350986 0.683230 1.337023
46 1 0 3.058151 -0.160035 3.173967
47 6 0 0.839067 -0.953926 4.373000
48 1 0 1.784315 -1.084835 4.900342
49 1 0 0.189388 -0.290994 4.950286
50 1 0 0.335042 -1.919000 4.275856
51 27 0 0.595743 0.464946 0.432092
52 7 0 -3.911121 1.028237 0.088330
53 7 0 -3.243955 -1.585758 -0.934339
54 6 0 -5.589876 -2.016334 -1.227516
55 1 0 -5.320128 -2.991116 -1.625564
56 6 0 -6.921998 -1.633520 -1.118256
57 1 0 -7.707531 -2.313256 -1.435249
58 6 0 -7.246429 -0.376868 -0.599509
59 6 0 -6.236120 0.490351 -0.198735
60 1 0 -6.469790 1.472827 0.203116
61 1 0 -8.286052 -0.076002 -0.508579
62 1 0 2.044540 2.678654 0.573107
63 6 0 3.600318 0.459186 0.659144
64 6 0 4.652481 0.249632 0.097228
65 6 0 5.892898 -0.008967 -0.574162
66 6 0 6.139489 -1.274443 -1.127928
67 6 0 6.865700 0.995278 -0.687022
68 6 0 7.340234 -1.526999 -1.780947
69 1 0 5.385602 -2.050694 -1.043005
70 6 0 8.062856 0.734419 -1.343909
71 1 0 6.677404 1.973171 -0.255767
72 6 0 8.302527 -0.524794 -1.891387
73 1 0 7.523251 -2.507690 -2.208454
74 1 0 8.811891 1.515526 -1.426507
75 1 0 9.239053 -0.724616 -2.402548
76 1 0 1.206737 2.320715 2.162516

77 1 0 1.509799 -0.206029 -0.456821
78 1 0 1.061441 0.902830 -0.877100

32

E(UwB97XD/6-311G++(d, p)) -3471.614362 a. u.

∆G(RwB97XD/6-31G(d, p)) = 0.581918 a. u.

Standard orientation:

Center Atomic Atomic Coordinates (Angstroms)
Number Number Type X Y Z
----
1 6 0 -2.589712 0.636368 0.045455
2 6 0 -2.345555 -0.560260 -0.620437
3 6 0 -4.584285 -0.787045 -1.080600
4 6 0 -4.841505 0.419761 -0.356185
5 15 0 -1.136745 1.599955 0.572642
6 15 0 -0.630806 -1.163185 -0.635385
7 6 0 -1.593839 2.232664 2.221247
8 1 0 -0.945429 3.065035 2.505752
9 1 0 -2.634784 2.563582 2.223677
10 1 0 -1.472470 1.425154 2.947281
11 6 0 -0.160829 -1.247038 -2.394149
12 6 0 -1.131441 3.093288 -0.567421
13 6 0 -1.240373 2.593182 -2.012572
14 1 0 -0.426727 1.904498 -2.259992
15 1 0 -2.192650 2.087764 -2.197951
16 1 0 -1.174649 3.447785 -2.695353
17 6 0 0.177726 3.871616 -0.395788
18 1 0 0.117835 4.801836 -0.972466
19 1 0 0.360190 4.141603 0.649588
20 1 0 1.034883 3.303016 -0.766358
21 6 0 -2.313002 4.021371 -0.248327
22 1 0 -2.202618 4.490927 0.734043
23 1 0 -2.343851 4.822686 -0.995751
24 1 0 -3.264673 3.484848 -0.271636
25 6 0 -0.704058 -2.952151 -0.057311
26 6 0 0.708562 -3.420938 0.316408
27 1 0 1.418506 -3.276787 -0.505428
28 1 0 1.094613 -2.902186 1.197193
29 1 0 0.677877 -4.492665 0.544029
30 6 0 -1.246421 -3.864617 -1.169901
31 1 0 -1.377418 -4.872738 -0.759166
32 1 0 -2.212474 -3.512768 -1.539082
33 1 0 -0.550707 -3.938931 -2.011164
34 6 0 -1.625926 -3.040112 1.167030
35 1 0 -2.665707 -2.836435 0.896215

36 1 0 -1.576582 -4.055531 1.577358
37 1 0 -1.323292 -2.340003 1.948570
38 1 0 0.792122 -1.771665 -2.501489
39 1 0 -0.931629 -1.759718 -2.973649
40 1 0 -0.046146 -0.231651 -2.777702
41 8 0 0.119562 -0.645425 2.311894
42 6 0 1.134059 -0.922655 2.997710
43 7 0 2.327898 -0.521058 2.568329
44 6 0 1.677355 1.588444 1.534375
45 6 0 2.382401 0.368136 1.428368
46 1 0 3.168050 -0.705898 3.101878
47 6 0 1.019197 -1.696271 4.267578
48 1 0 1.990716 -1.886700 4.724455
49 1 0 0.393422 -1.133071 4.964715
50 1 0 0.518202 -2.644403 4.056717
51 27 0 0.601438 0.213822 0.548426
52 7 0 -3.824137 1.158724 0.210623
53 7 0 -3.311072 -1.296141 -1.217090
54 6 0 -5.667041 -1.471467 -1.661305
55 1 0 -5.454876 -2.385193 -2.210459
56 6 0 -6.969301 -1.002264 -1.532224
57 1 0 -7.789350 -1.548182 -1.989499
58 6 0 -7.221403 0.170641 -0.815007
59 6 0 -6.167938 0.868724 -0.235303
60 1 0 -6.344773 1.786830 0.318946
61 1 0 -8.237509 0.539584 -0.710141
62 1 0 2.007940 2.423610 0.932820
63 6 0 3.594799 0.269344 0.660392
64 6 0 4.627867 0.197701 0.033894
65 6 0 5.855205 0.117855 -0.702822
66 6 0 6.183576 -1.051128 -1.405453
67 6 0 6.736017 1.209379 -0.726933
68 6 0 7.376925 -1.124823 -2.114567
69 1 0 5.499486 -1.893555 -1.389918
70 6 0 7.925954 1.127423 -1.440617
71 1 0 6.481927 2.114282 -0.184496
72 6 0 8.249137 -0.037876 -2.133774
73 1 0 7.625441 -2.033030 -2.654365
74 1 0 8.604644 1.974333 -1.453971
75 1 0 9.181007 -0.099289 -2.686989
76 1 0 1.229631 1.841511 2.491167
77 1 0 1.439126 -0.891592 0.100640
78 1 0 1.024924 0.797573 -0.713146

TS20

E(UwB97XD/6-311G++(d, p)) - 3472.764991 a. u.

∆G(RwB97XD/6-31G(d, p)) = 0.579478 a. u.

$\nu = i\ 535.25$

Standard orientation:

Center Atomic Atomic Coordinates (Angstroms)
Number Number Type X Y Z

----

1 6 0 -2.636596 0.618763 -0.005625
2 6 0 -2.360306 -0.614057 -0.591543
3 6 0 -4.596623 -0.936120 -1.013549
4 6 0 -4.883935 0.311752 -0.375006
5 15 0 -1.216298 1.650721 0.479595
6 15 0 -0.627269 -1.168234 -0.589765
7 6 0 -1.681529 2.323518 2.108975
8 1 0 -1.018408 3.148093 2.382651
9 1 0 -2.716612 2.671921 2.099160
10 1 0 -1.578039 1.527978 2.850448
11 6 0 -0.178149 -1.319612 -2.351783
12 6 0 -1.241748 3.103838 -0.704483
13 6 0 -1.348553 2.557809 -2.133119
14 1 0 -0.534478 1.861990 -2.359629
15 1 0 -2.300304 2.044837 -2.300597
16 1 0 -1.285783 3.389737 -2.843664
17 6 0 0.060631 3.898112 -0.552740
18 1 0 -0.007621 4.817196 -1.146103
19 1 0 0.243928 4.186706 0.487644
20 1 0 0.921513 3.327856 -0.911036
21 6 0 -2.433913 4.026325 -0.410274
22 1 0 -2.330152 4.521012 0.560412
23 1 0 -2.473749 4.808701 -1.177055
24 1 0 -3.379438 3.478246 -0.421044
25 6 0 -0.630006 -2.927356 0.068379
26 6 0 0.811892 -3.312093 0.429464
27 1 0 1.497200 -3.168608 -0.413277
28 1 0 1.190204 -2.736466 1.278472
29 1 0 0.842857 -4.372482 0.705225
30 6 0 -1.161397 -3.916718 -0.980730
31 1 0 -1.244009 -4.908191 -0.519728
32 1 0 -2.148584 -3.619331 -1.342140
33 1 0 -0.484388 -4.006307 -1.835785
34 6 0 -1.515758 -2.983210 1.320662
35 1 0 -2.568646 -2.826223 1.069718
36 1 0 -1.421734 -3.973312 1.782145
37 1 0 -1.217878 -2.233365 2.057015
38 1 0 0.794784 -1.808663 -2.450258
39 1 0 -0.931647 -1.889613 -2.899956
40 1 0 -0.108912 -0.318296 -2.781629
41 8 0 0.115582 -0.412682 2.350007
42 6 0 1.142814 -0.697254 3.012373
43 7 0 2.338312 -0.396442 2.507376

44 6 0 1.728394 1.674150 1.366661
45 6 0 2.385544 0.401426 1.295519
46 1 0 3.191996 -0.588468 3.015593
47 6 0 1.042463 -1.380784 4.334104
48 1 0 2.017254 -1.530115 4.799264
49 1 0 0.410869 -0.779225 4.992236
50 1 0 0.552975 -2.347706 4.189616
51 27 0 0.550221 0.335319 0.520513
52 7 0 -3.885245 1.117761 0.128997
53 7 0 -3.309517 -1.414684 -1.128681
54 6 0 -5.663292 -1.695750 -1.526098
55 1 0 -5.428383 -2.639953 -2.010596
56 6 0 -6.978741 -1.259505 -1.414089
57 1 0 -7.785644 -1.865254 -1.816117
58 6 0 -7.259818 -0.044998 -0.782062
59 6 0 -6.222749 0.727758 -0.271632
60 1 0 -6.422170 1.676963 0.218845
61 1 0 -8.286243 0.297800 -0.689513
62 1 0 2.097770 2.463814 0.725077
63 6 0 3.630229 0.273169 0.573445
64 6 0 4.681757 0.164791 -0.014040
65 6 0 5.935779 0.048300 -0.699403
66 6 0 6.258648 -1.132280 -1.384392
67 6 0 6.846403 1.115075 -0.689064
68 6 0 7.477342 -1.241754 -2.043896
69 1 0 5.550914 -1.954915 -1.393996
70 6 0 8.062438 0.996523 -1.351794
71 1 0 6.595338 2.028555 -0.159849
72 6 0 8.379942 -0.179797 -2.028749
73 1 0 7.722176 -2.158104 -2.571331
74 1 0 8.765282 1.823416 -1.339192
75 1 0 9.331467 -0.269843 -2.543133
76 1 0 1.368449 1.997288 2.340271
77 1 0 1.604398 -0.660484 0.288300
78 1 0 0.934490 0.846787 -0.784759

34

E(UwB97XD/6-311G++(d, p)) = -3472.773728 a. u.

ΔG(RwB97XD/6-31G(d, p)) = 0.583951 a. u.

Standard orientation:

\---

Center Atomic Atomic Coordinates (Angstroms)
Number Number Type X Y Z

\---

1 6 0 -2.724980 0.608165 -0.004870
2 6 0 -2.476069 -0.646807 -0.556749
3 6 0 -4.732250 -0.982641 -0.850639
4 6 0 -4.988198 0.292296 -0.253439
5 15 0 -1.293680 1.657447 0.407898
6 15 0 -0.741156 -1.191814 -0.644858
7 6 0 -1.673729 2.314227 2.064838
8 1 0 -0.984242 3.123674 2.317463
9 1 0 -2.701942 2.680146 2.107102
10 1 0 -1.549856 1.507262 2.790361
11 6 0 -0.419420 -1.373572 -2.434796
12 6 0 -1.371479 3.119205 -0.757857
13 6 0 -1.529620 2.589704 -2.187934
14 1 0 -0.719921 1.903847 -2.456267
15 1 0 -2.483629 2.071080 -2.323580
16 1 0 -1.502348 3.430942 -2.889781
17 6 0 -0.068330 3.918636 -0.637066
18 1 0 -0.163593 4.849157 -1.208303
19 1 0 0.149326 4.185812 0.402671
20 1 0 0.783521 3.360806 -1.033297
21 6 0 -2.558920 4.029535 -0.409181
22 1 0 -2.422443 4.517581 0.560713
23 1 0 -2.633817 4.817704 -1.167400
24 1 0 -3.499801 3.473851 -0.388814
25 6 0 -0.674824 -2.935144 0.043654
26 6 0 0.802724 -3.264385 0.306820
27 1 0 1.412231 -3.142280 -0.595554
28 1 0 1.229767 -2.634912 1.092979
29 1 0 0.894281 -4.307504 0.630728
30 6 0 -1.254493 -3.967808 -0.933142
31 1 0 -1.257819 -4.952009 -0.449340
32 1 0 -2.280442 -3.715098 -1.211955
33 1 0 -0.653175 -4.051186 -1.843635
34 6 0 -1.465533 -2.970473 1.358216
35 1 0 -2.535238 -2.829642 1.177886
36 1 0 -1.329244 -3.947649 1.836785
37 1 0 -1.127114 -2.198758 2.054266
38 1 0 0.564806 -1.822442 -2.595676
39 1 0 -1.183099 -1.988623 -2.916257
40 1 0 -0.426761 -0.379824 -2.888217
41 8 0 0.189606 -0.306403 2.245038
42 6 0 1.227868 -0.613393 2.878287
43 7 0 2.421432 -0.417830 2.317602
44 6 0 1.797834 1.606532 1.055198
45 6 0 2.475479 0.262077 1.023760
46 1 0 3.277492 -0.643412 2.806045
47 6 0 1.141252 -1.230650 4.233985
48 1 0 2.120517 -1.355429 4.697023
49 1 0 0.510233 -0.603496 4.867603
50 1 0 0.657735 -2.206878 4.136757

51 27 0 0.459845 0.406694 0.374635
52 7 0 -3.967012 1.114188 0.170834
53 7 0 -3.451721 -1.467201 -1.009452
54 6 0 -5.822402 -1.761780 -1.276762
55 1 0 -5.611091 -2.726246 -1.731282
56 6 0 -7.131145 -1.318283 -1.120799
57 1 0 -7.956326 -1.939599 -1.456548
58 6 0 -7.381241 -0.076504 -0.530271
59 6 0 -6.320753 0.715553 -0.104473
60 1 0 -6.496448 1.684779 0.354933
61 1 0 -8.402001 0.272477 -0.403909
62 1 0 2.236914 2.338823 0.383230
63 6 0 3.807155 0.208930 0.440331
64 6 0 4.918179 0.133476 -0.028721
65 6 0 6.228029 0.034007 -0.603922
66 6 0 6.568228 -1.091855 -1.368248
67 6 0 7.174306 1.049710 -0.405818
68 6 0 7.836228 -1.195398 -1.927299
69 1 0 5.834179 -1.876805 -1.518665
70 6 0 8.440862 0.936301 -0.967618
71 1 0 6.910285 1.920393 0.185655
72 6 0 8.773722 -0.183420 -1.728077
73 1 0 8.093621 -2.068543 -2.518239
74 1 0 9.170563 1.724402 -0.811969
75 1 0 9.763152 -0.267583 -2.166280
76 1 0 1.686516 1.997053 2.067183
77 1 0 1.888809 -0.472788 0.295152
78 1 0 0.716504 0.915376 -0.967977

TS22

E(UwB97XD/6-311G++(d, p)) - 3472.744477 a. u.

ΔG(RwB97XD/6-31G(d, p)) = 0.578658 a. u.

$\nu = i$ 893.79

Standard orientation:

Center Atomic Atomic Coordinates (Angstroms)
Number Number Type X Y Z
----
1 6 0 -2.780614 0.260286 -0.291872
2 6 0 -2.247769 -1.019166 -0.174092
3 6 0 -4.316656 -1.938988 -0.568380
4 6 0 -4.872226 -0.622425 -0.651659
5 15 0 -1.610965 1.652359 -0.167653

6 15 0 -0.485522 -1.123275 0.265712
7 6 0 -2.302117 2.675269 1.178211
8 1 0 -1.795863 3.642989 1.223585
9 1 0 -3.374239 2.827696 1.033509
10 1 0 -2.137909 2.154960 2.124195
11 6 0 0.197664 -2.283186 -0.977371
12 6 0 -1.838234 2.657393 -1.733461
13 6 0 -1.691787 1.699271 -2.922064
14 1 0 -0.739477 1.158264 -2.887912
15 1 0 -2.502908 0.965206 -2.947510
16 1 0 -1.721935 2.268302 -3.858334
17 6 0 -0.750103 3.739607 -1.796791
18 1 0 -0.974004 4.423764 -2.623255
19 1 0 -0.709074 4.332608 -0.876232
20 1 0 0.239921 3.315154 -1.976072
21 6 0 -3.213008 3.341472 -1.778067
22 1 0 -3.300982 4.113950 -1.007367
23 1 0 -3.333650 3.832856 -2.750994
24 1 0 -4.023139 2.621544 -1.643753
25 6 0 -0.421533 -2.064843 1.889095
26 6 0 1.026676 -2.023367 2.401625
27 1 0 1.737050 -2.410100 1.661985
28 1 0 1.331275 -1.007793 2.673118
29 1 0 1.114323 -2.644219 3.300876
30 6 0 -0.865153 -3.526592 1.750908
31 1 0 -0.904033 -3.988882 2.744938
32 1 0 -1.853382 -3.601104 1.290488
33 1 0 -0.160033 -4.106381 1.146543
34 6 0 -1.333515 -1.328478 2.878843
35 1 0 -2.385560 -1.394378 2.583361
36 1 0 -1.235827 -1.782333 3.872204
37 1 0 -1.061032 -0.270935 2.964188
38 1 0 1.233782 -2.535863 -0.734508
39 1 0 -0.395325 -3.198805 -1.044718
40 1 0 0.184718 -1.778325 -1.946921
41 8 0 0.476605 1.623956 2.277355
42 6 0 1.634466 1.811997 2.696525
43 7 0 2.688413 1.509195 1.922718
44 6 0 1.827417 2.066437 -0.353424
45 6 0 2.470819 1.030585 0.564975
46 1 0 3.633953 1.667848 2.242782
47 6 0 1.892158 2.353712 4.068111
48 1 0 1.496816 1.645126 4.800569
49 1 0 2.952964 2.519336 4.260910
50 1 0 1.348232 3.294115 4.182279
51 27 0 0.373475 0.883644 0.266188
52 7 0 -4.092754 0.504610 -0.515263
53 7 0 -2.977616 -2.148416 -0.320201
54 6 0 -5.169034 -3.045018 -0.734353
55 1 0 -4.729429 -4.037017 -0.668758

56 6 0 -6.529653 -2.879876 -0.969263
57 1 0 -7.167175 -3.750464 -1.093114
58 6 0 -7.073323 -1.594529 -1.043997
59 6 0 -6.251740 -0.483357 -0.887196
60 1 0 -6.658488 0.522927 -0.946355
61 1 0 -8.135658 -1.460221 -1.225723
62 1 0 2.371736 2.198631 -1.289728
63 6 0 3.670776 0.409692 0.018746
64 6 0 4.684483 -0.097571 -0.399023
65 6 0 5.880872 -0.687461 -0.925018
66 6 0 6.136902 -2.052965 -0.734989
67 6 0 6.801531 0.100731 -1.630613
68 6 0 7.299081 -2.618261 -1.246730
69 1 0 5.423379 -2.660966 -0.188316
70 6 0 7.961151 -0.473578 -2.137695
71 1 0 6.600752 1.156954 -1.777408
72 6 0 8.211545 -1.831322 -1.947215
73 1 0 7.493074 -3.675522 -1.097544
74 1 0 8.670376 0.140375 -2.683419
75 1 0 9.117657 -2.276489 -2.345881
76 1 0 1.710236 3.040984 0.125319
77 1 0 1.775852 0.087934 0.689785
78 1 0 0.852900 1.351440 -1.056968

TS17

E(UwB97XD/6-311G++(d, p)) -3472.758847 a. u.

ΔG(RwB97XD/6-31G(d, p)) = 0.578303 a. u.

ν = *i*802.77

Standard orientation:

Center Atomic Atomic Coordinates (Angstroms)
Number Number Type X Y Z
----
1 6 0 -2.647371 0.592409 -0.013055
2 6 0 -2.342867 -0.648919 -0.567462
3 6 0 -4.577090 -1.087871 -0.875146
4 6 0 -4.892830 0.171038 -0.272782
5 15 0 -1.249943 1.683237 0.417115
6 15 0 -0.588705 -1.115361 -0.671380
7 6 0 -1.719538 2.371921 2.041759
8 1 0 -1.093287 3.232833 2.288634
9 1 0 -2.770275 2.670655 2.041166
10 1 0 -1.569683 1.599524 2.799795

11 6 0 -0.233957 -1.165924 -2.461558
12 6 0 -1.336845 3.127786 -0.775244
13 6 0 -1.346948 2.574234 -2.203928
14 1 0 -0.451328 1.977149 -2.402794
15 1 0 -2.229067 1.956526 -2.398877
16 1 0 -1.355074 3.406915 -2.916388
17 6 0 -0.090598 4.000505 -0.578154
18 1 0 -0.180202 4.901035 -1.196573
19 1 0 0.026552 4.322520 0.461903
20 1 0 0.818014 3.472242 -0.879576
21 6 0 -2.595227 3.972792 -0.533369
22 1 0 -2.568594 4.464925 0.443695
23 1 0 -2.649279 4.757979 -1.296582
24 1 0 -3.504265 3.368578 -0.589164
25 6 0 -0.428333 -2.897426 -0.105058
26 6 0 1.059385 -3.152108 0.178858
27 1 0 1.684109 -2.907927 -0.687946
28 1 0 1.418905 -2.567511 1.030496
29 1 0 1.209162 -4.213182 0.408987
30 6 0 -0.912850 -3.876616 -1.185644
31 1 0 -0.872818 -4.893922 -0.778264
32 1 0 -1.943304 -3.662893 -1.479386
33 1 0 -0.278736 -3.854948 -2.076846
34 6 0 -1.257859 -3.109251 1.168408
35 1 0 -2.327956 -3.027354 0.957921
36 1 0 -1.065001 -4.115868 1.557494
37 1 0 -1.002380 -2.385468 1.944071
38 1 0 0.738028 -1.634699 -2.636733
39 1 0 -1.010855 -1.720007 -2.992991
40 1 0 -0.204099 -0.145121 -2.847061
41 8 0 0.042624 -0.386623 2.183461
42 6 0 1.012982 -0.623643 2.947434
43 7 0 2.232320 -0.293480 2.537345
44 6 0 2.348061 0.531641 1.353670
45 6 0 1.672146 1.789973 1.373971
46 1 0 2.065334 2.587835 0.755293
47 1 0 3.043430 -0.457098 3.121461
48 6 0 0.808379 -1.248448 4.285492
49 1 0 0.384118 -0.498817 4.960228
50 1 0 0.091336 -2.066559 4.193867
51 1 0 1.742781 -1.617339 4.710022
52 1 0 1.222810 2.112591 2.310094
53 6 0 3.600934 0.360860 0.669362
54 6 0 4.661363 0.208768 0.104288
55 6 0 5.924417 0.039082 -0.550153
56 6 0 6.550516 1.128043 -1.175625
57 6 0 6.548335 -1.217472 -0.566536
58 6 0 7.778501 0.957835 -1.804517
59 1 0 6.069496 2.100681 -1.163200
60 6 0 7.778720 -1.376278 -1.193663

61 1 0 6.064760 -2.061507 -0.085341
62 6 0 8.396065 -0.291548 -1.814128
63 1 0 8.255761 1.805408 -2.286138
64 1 0 8.256287 -2.350875 -1.199415
65 1 0 9.356128 -0.419795 -2.303965
66 27 0 0.589439 0.411947 0.439055
67 7 0 -3.911735 1.038205 0.159070
68 7 0 -3.275712 -1.512128 -1.031600
69 6 0 -5.628949 -1.913991 -1.309188
70 1 0 -5.372122 -2.865596 -1.767302
71 6 0 -6.956890 -1.531986 -1.154914
72 1 0 -7.751452 -2.189238 -1.495964
73 6 0 -7.265521 -0.305993 -0.558872
74 6 0 -6.243668 0.531764 -0.126004
75 1 0 -6.464467 1.489916 0.337148
76 1 0 -8.301806 -0.006279 -0.432954
77 1 0 1.057694 0.956134 -0.834782
78 1 0 1.496328 -0.164413 -0.525379

33

E(UwB97XD/6-311G++(d, p)) = -3472.767362 a. u.

ΔG(RwB97XD/6-31G(d, p)) = 0.58187 a. u.

Standard orientation:

Center Atomic Atomic Coordinates (Angstroms)
Number Number Type X Y Z
----
1 6 0 -2.589687 0.636458 0.045570
2 6 0 -2.345980 -0.560248 -0.620359
3 6 0 -4.584941 -0.786672 -1.079629
4 6 0 -4.841685 0.420140 -0.355059
5 15 0 -1.136385 1.599959 0.572084
6 15 0 -0.631341 -1.163400 -0.635970
7 6 0 -1.592903 2.232997 2.220715
8 1 0 -0.945397 3.066387 2.504312
9 1 0 -2.634310 2.562461 2.223694
10 1 0 -1.469940 1.426036 2.947097
11 6 0 -0.161889 -1.247413 -2.394870
12 6 0 -1.131273 3.093084 -0.568266
13 6 0 -1.240685 2.592727 -2.013293
14 1 0 -0.427064 1.904078 -2.260859
15 1 0 -2.192984 2.087198 -2.198262
16 1 0 -1.175267 3.447215 -2.696247
17 6 0 0.178056 3.871282 -0.397185
18 1 0 0.118174 4.801314 -0.974160

19 1 0 0.360754 4.141613 0.648061
20 1 0 1.035055 3.302424 -0.767726
21 6 0 -2.312601 4.021371 -0.248987
22 1 0 -2.202020 4.490825 0.733412
23 1 0 -2.343384 4.822753 -0.996343
24 1 0 -3.264387 3.485051 -0.272212
25 6 0 -0.704694 -2.952440 -0.057964
26 6 0 0.707990 -3.421715 0.314902
27 1 0 1.417507 -3.277795 -0.507339
28 1 0 1.094716 -2.903098 1.195473
29 1 0 0.677088 -4.493439 0.542534
30 6 0 -1.247964 -3.864704 -1.170265
31 1 0 -1.379103 -4.872796 -0.759501
32 1 0 -2.214092 -3.512506 -1.538925
33 1 0 -0.552718 -3.939250 -2.011896
34 6 0 -1.625870 -3.040284 1.166921
35 1 0 -2.665802 -2.836605 0.896694
36 1 0 -1.576310 -4.055670 1.577306
37 1 0 -1.322777 -2.340122 1.948232
38 1 0 0.791199 -1.771765 -2.502347
39 1 0 -0.932654 -1.760446 -2.974103
40 1 0 -0.047632 -0.232084 -2.778695
41 8 0 0.119520 -0.645238 2.311238
42 6 0 1.133873 -0.922080 2.997391
43 7 0 2.327806 -0.520628 2.568076
44 6 0 1.677334 1.588507 1.533380
45 6 0 2.382469 0.368271 1.427942
46 1 0 3.167847 -0.705218 3.101886
47 6 0 1.018916 -1.695682 4.267253
48 1 0 1.990519 -1.887354 4.723423
49 1 0 0.394394 -1.131711 4.964904
50 1 0 0.516562 -2.643154 4.056711
51 27 0 0.601533 0.213443 0.547589
52 7 0 -3.823963 1.158943 0.211330
53 7 0 -3.311869 -1.295970 -1.216638
54 6 0 -5.668043 -1.470881 -1.659939
55 1 0 -5.456254 -2.384609 -2.209235
56 6 0 -6.970175 -1.001474 -1.530305
57 1 0 -7.790489 -1.547237 -1.987291
58 6 0 -7.221809 0.171417 -0.812901
59 6 0 -6.167997 0.869294 -0.233582
60 1 0 -6.344446 1.787396 0.320797
61 1 0 -8.237815 0.540513 -0.707604
62 1 0 2.007934 2.423480 0.931572
63 6 0 3.595022 0.269282 0.660184
64 6 0 4.628307 0.197355 0.034085
65 6 0 5.855980 0.117667 -0.702093
66 6 0 6.184636 -1.051211 -1.404752
67 6 0 6.736849 1.209168 -0.725602
68 6 0 7.378331 -1.124838 -2.113289

69 1 0 5.500507 -1.893614 -1.389673
70 6 0 7.927134 1.127281 -1.438717
71 1 0 6.482533 2.113991 -0.183138
72 6 0 8.250602 -0.037926 -2.131896
73 1 0 7.627066 -2.032967 -2.653116
74 1 0 8.605872 1.974159 -1.451612
75 1 0 9.182750 -0.099323 -2.684643
76 1 0 1.229301 1.841761 2.489977
77 1 0 1.439947 -0.892195 0.101163
78 1 0 1.025799 0.796059 -0.714554

TS21

E(UwB97XD/6-311G++(d, p)) - 3472.764991 a. u.

ΔG(RwB97XD/6-31G(d, p)) = 0.579478 a. u.

ν = *i* 535.25

Standard orientation:

Center Atomic Atomic Coordinates (Angstroms)
Number Number Type X Y Z
----
1 6 0 -2.636596 0.618763 -0.005625
2 6 0 -2.360306 -0.614057 -0.591543
3 6 0 -4.596623 -0.936120 -1.013549
4 6 0 -4.883935 0.311752 -0.375006
5 15 0 -1.216298 1.650721 0.479595
6 15 0 -0.627269 -1.168234 -0.589765
7 6 0 -1.681529 2.323518 2.108975
8 1 0 -1.018408 3.148093 2.382651
9 1 0 -2.716612 2.671921 2.099160
10 1 0 -1.578039 1.527978 2.850448
11 6 0 -0.178149 -1.319612 -2.351783
12 6 0 -1.241748 3.103838 -0.704483
13 6 0 -1.348553 2.557809 -2.133119
14 1 0 -0.534478 1.861990 -2.359629
15 1 0 -2.300304 2.044837 -2.300597
16 1 0 -1.285783 3.389737 -2.843664
17 6 0 0.060631 3.898112 -0.552740
18 1 0 -0.007621 4.817196 -1.146103
19 1 0 0.243928 4.186706 0.487644
20 1 0 0.921513 3.327856 -0.911036
21 6 0 -2.433913 4.026325 -0.410274
22 1 0 -2.330152 4.521012 0.560412
23 1 0 -2.473749 4.808701 -1.177055

24 1 0 -3.379438 3.478246 -0.421044
25 6 0 -0.630006 -2.927356 0.068379
26 6 0 0.811892 -3.312093 0.429464
27 1 0 1.497200 -3.168608 -0.413277
28 1 0 1.190204 -2.736466 1.278472
29 1 0 0.842857 -4.372482 0.705225
30 6 0 -1.161397 -3.916718 -0.980730
31 1 0 -1.244009 -4.908191 -0.519728
32 1 0 -2.148584 -3.619331 -1.342140
33 1 0 -0.484388 -4.006307 -1.835785
34 6 0 -1.515758 -2.983210 1.320662
35 1 0 -2.568646 -2.826223 1.069718
36 1 0 -1.421734 -3.973312 1.782145
37 1 0 -1.217878 -2.233365 2.057015
38 1 0 0.794784 -1.808663 -2.450258
39 1 0 -0.931647 -1.889613 -2.899956
40 1 0 -0.108912 -0.318296 -2.781629
41 8 0 0.115582 -0.412682 2.350007
42 6 0 1.142814 -0.697254 3.012373
43 7 0 2.338312 -0.396442 2.507376
44 6 0 1.728394 1.674150 1.366661
45 6 0 2.385544 0.401426 1.295519
46 1 0 3.191996 -0.588468 3.015593
47 6 0 1.042463 -1.380784 4.334104
48 1 0 2.017254 -1.530115 4.799264
49 1 0 0.410869 -0.779225 4.992236
50 1 0 0.552975 -2.347706 4.189616
51 27 0 0.550221 0.335319 0.520513
52 7 0 -3.885245 1.117761 0.128997
53 7 0 -3.309517 -1.414684 -1.128681
54 6 0 -5.663292 -1.695750 -1.526098
55 1 0 -5.428383 -2.639953 -2.010596
56 6 0 -6.978741 -1.259505 -1.414089
57 1 0 -7.785644 -1.865254 -1.816117
58 6 0 -7.259818 -0.044998 -0.782062
59 6 0 -6.222749 0.727758 -0.271632
60 1 0 -6.422170 1.676963 0.218845
61 1 0 -8.286243 0.297800 -0.689513
62 1 0 2.097770 2.463814 0.725077
63 6 0 3.630229 0.273169 0.573445
64 6 0 4.681757 0.164791 -0.014040
65 6 0 5.935779 0.048300 -0.699403
66 6 0 6.258648 -1.132280 -1.384392
67 6 0 6.846403 1.115075 -0.689064
68 6 0 7.477342 -1.241754 -2.043896
69 1 0 5.550914 -1.954915 -1.393996
70 6 0 8.062438 0.996523 -1.351794
71 1 0 6.595338 2.028555 -0.159849
72 6 0 8.379942 -0.179797 -2.028749
73 1 0 7.722176 -2.158104 -2.571331

74 1 0 8.765282 1.823416 -1.339192
75 1 0 9.331467 -0.269843 -2.543133
76 1 0 1.368449 1.997288 2.340271
77 1 0 1.604398 -0.660484 0.288300
78 1 0 0.934490 0.846787 -0.784759

35

E(UwB97XD/6-311G++(d, p)) = -3472.773728 a. u.

ΔG(RwB97XD/6-31G(d, p)) = 0.583951 a. u.

Standard orientation:

Center Atomic Atomic Coordinates (Angstroms)
Number Number Type X Y Z
----
1 6 0 -2.724980 0.608165 -0.004870
2 6 0 -2.476069 -0.646807 -0.556749
3 6 0 -4.732250 -0.982641 -0.850639
4 6 0 -4.988198 0.292296 -0.253439
5 15 0 -1.293680 1.657447 0.407898
6 15 0 -0.741156 -1.191814 -0.644858
7 6 0 -1.673729 2.314227 2.064838
8 1 0 -0.984242 3.123674 2.317463
9 1 0 -2.701942 2.680146 2.107102
10 1 0 -1.549856 1.507262 2.790361
11 6 0 -0.419420 -1.373572 -2.434796
12 6 0 -1.371479 3.119205 -0.757857
13 6 0 -1.529620 2.589704 -2.187934
14 1 0 -0.719921 1.903847 -2.456267
15 1 0 -2.483629 2.071080 -2.323580
16 1 0 -1.502348 3.430942 -2.889781
17 6 0 -0.068330 3.918636 -0.637066
18 1 0 -0.163593 4.849157 -1.208303
19 1 0 0.149326 4.185812 0.402671
20 1 0 0.783521 3.360806 -1.033297
21 6 0 -2.558920 4.029535 -0.409181
22 1 0 -2.422443 4.517581 0.560713
23 1 0 -2.633817 4.817704 -1.167400
24 1 0 -3.499801 3.473851 -0.388814
25 6 0 -0.674824 -2.935144 0.043654
26 6 0 0.802724 -3.264385 0.306820
27 1 0 1.412231 -3.142280 -0.595554
28 1 0 1.229767 -2.634912 1.092979

29 1 0 0.894281 -4.307504 0.630728
30 6 0 -1.254493 -3.967808 -0.933142
31 1 0 -1.257819 -4.952009 -0.449340
32 1 0 -2.280442 -3.715098 -1.211955
33 1 0 -0.653175 -4.051186 -1.843635
34 6 0 -1.465533 -2.970473 1.358216
35 1 0 -2.535238 -2.829642 1.177886
36 1 0 -1.329244 -3.947649 1.836785
37 1 0 -1.127114 -2.198758 2.054266
38 1 0 0.564806 -1.822442 -2.595676
39 1 0 -1.183099 -1.988623 -2.916257
40 1 0 -0.426761 -0.379824 -2.888217
41 8 0 0.189606 -0.306403 2.245038
42 6 0 1.227868 -0.613393 2.878287
43 7 0 2.421432 -0.417830 2.317602
44 6 0 1.797834 1.606532 1.055198
45 6 0 2.475479 0.262077 1.023760
46 1 0 3.277492 -0.643412 2.806045
47 6 0 1.141252 -1.230650 4.233985
48 1 0 2.120517 -1.355429 4.697023
49 1 0 0.510233 -0.603496 4.867603
50 1 0 0.657735 -2.206878 4.136757
51 27 0 0.459845 0.406694 0.374635
52 7 0 -3.967012 1.114188 0.170834
53 7 0 -3.451721 -1.467201 -1.009452
54 6 0 -5.822402 -1.761780 -1.276762
55 1 0 -5.611091 -2.726246 -1.731282
56 6 0 -7.131145 -1.318283 -1.120799
57 1 0 -7.956326 -1.939599 -1.456548
58 6 0 -7.381241 -0.076504 -0.530271
59 6 0 -6.320753 0.715553 -0.104473
60 1 0 -6.496448 1.684779 0.354933
61 1 0 -8.402001 0.272477 -0.403909
62 1 0 2.236914 2.338823 0.383230
63 6 0 3.807155 0.208930 0.440331
64 6 0 4.918179 0.133476 -0.028721
65 6 0 6.228029 0.034007 -0.603922
66 6 0 6.568228 -1.091855 -1.368248
67 6 0 7.174306 1.049710 -0.405818
68 6 0 7.836228 -1.195398 -1.927299
69 1 0 5.834179 -1.876805 -1.518665
70 6 0 8.440862 0.936301 -0.967618
71 1 0 6.910285 1.920393 0.185655
72 6 0 8.773722 -0.183420 -1.728077
73 1 0 8.093621 -2.068543 -2.518239
74 1 0 9.170563 1.724402 -0.811969
75 1 0 9.763152 -0.267583 -2.166280
76 1 0 1.686516 1.997053 2.067183
77 1 0 1.888809 -0.472788 0.295152
78 1 0 0.716504 0.915376 -0.967977

TS23

E(UwB97XD/6-311G++(d, p)) -3472.744477 a. u.

ΔG(RwB97XD/6-31G(d, p)) = 0.578658 a. u.

ν = *i*893.79

Standard orientation:

Center Atomic Atomic Coordinates (Angstroms)
Number Number Type X Y Z
----
1 6 0 -2.780614 0.260286 -0.291872
2 6 0 -2.247769 -1.019166 -0.174092
3 6 0 -4.316656 -1.938988 -0.568380
4 6 0 -4.872226 -0.622425 -0.651659
5 15 0 -1.610965 1.652359 -0.167653
6 15 0 -0.485522 -1.123275 0.265712
7 6 0 -2.302117 2.675269 1.178211
8 1 0 -1.795863 3.642989 1.223585
9 1 0 -3.374239 2.827696 1.033509
10 1 0 -2.137909 2.154960 2.124195
11 6 0 0.197664 -2.283186 -0.977371
12 6 0 -1.838234 2.657393 -1.733461
13 6 0 -1.691787 1.699271 -2.922064
14 1 0 -0.739477 1.158264 -2.887912
15 1 0 -2.502908 0.965206 -2.947510
16 1 0 -1.721935 2.268302 -3.858334
17 6 0 -0.750103 3.739607 -1.796791
18 1 0 -0.974004 4.423764 -2.623255
19 1 0 -0.709074 4.332608 -0.876232
20 1 0 0.239921 3.315154 -1.976072
21 6 0 -3.213008 3.341472 -1.778067
22 1 0 -3.300982 4.113950 -1.007367
23 1 0 -3.333650 3.832856 -2.750994
24 1 0 -4.023139 2.621544 -1.643753
25 6 0 -0.421533 -2.064843 1.889095
26 6 0 1.026676 -2.023367 2.401625
27 1 0 1.737050 -2.410100 1.661985
28 1 0 1.331275 -1.007793 2.673118
29 1 0 1.114323 -2.644219 3.300876
30 6 0 -0.865153 -3.526592 1.750908
31 1 0 -0.904033 -3.988882 2.744938
32 1 0 -1.853382 -3.601104 1.290488
33 1 0 -0.160033 -4.106381 1.146543

34 6 0 -1.333515 -1.328478 2.878843
35 1 0 -2.385560 -1.394378 2.583361
36 1 0 -1.235827 -1.782333 3.872204
37 1 0 -1.061032 -0.270935 2.964188
38 1 0 1.233782 -2.535863 -0.734508
39 1 0 -0.395325 -3.198805 -1.044718
40 1 0 0.184718 -1.778325 -1.946921
41 8 0 0.476605 1.623956 2.277355
42 6 0 1.634466 1.811997 2.696525
43 7 0 2.688413 1.509195 1.922718
44 6 0 1.827417 2.066437 -0.353424
45 6 0 2.470819 1.030585 0.564975
46 1 0 3.633953 1.667848 2.242782
47 6 0 1.892158 2.353712 4.068111
48 1 0 1.496816 1.645126 4.800569
49 1 0 2.952964 2.519336 4.260910
50 1 0 1.348232 3.294115 4.182279
51 27 0 0.373475 0.883644 0.266188
52 7 0 -4.092754 0.504610 -0.515263
53 7 0 -2.977616 -2.148416 -0.320201
54 6 0 -5.169034 -3.045018 -0.734353
55 1 0 -4.729429 -4.037017 -0.668758
56 6 0 -6.529653 -2.879876 -0.969263
57 1 0 -7.167175 -3.750464 -1.093114
58 6 0 -7.073323 -1.594529 -1.043997
59 6 0 -6.251740 -0.483357 -0.887196
60 1 0 -6.658488 0.522927 -0.946355
61 1 0 -8.135658 -1.460221 -1.225723
62 1 0 2.371736 2.198631 -1.289728
63 6 0 3.670776 0.409692 0.018746
64 6 0 4.684483 -0.097571 -0.399023
65 6 0 5.880872 -0.687461 -0.925018
66 6 0 6.136902 -2.052965 -0.734989
67 6 0 6.801531 0.100731 -1.630613
68 6 0 7.299081 -2.618261 -1.246730
69 1 0 5.423379 -2.660966 -0.188316
70 6 0 7.961151 -0.473578 -2.137695
71 1 0 6.600752 1.156954 -1.777408
72 6 0 8.211545 -1.831322 -1.947215
73 1 0 7.493074 -3.675522 -1.097544
74 1 0 8.670376 0.140375 -2.683419
75 1 0 9.117657 -2.276489 -2.345881
76 1 0 1.710236 3.040984 0.125319
77 1 0 1.775852 0.087934 0.689785
78 1 0 0.852900 1.351440 -1.056968
